# STAT3 Signaling Pathway in Health and Disease

**DOI:** 10.1002/mco2.70152

**Published:** 2025-03-30

**Authors:** Md Abdus Samad, Iftikhar Ahmad, Aakifah Hasan, Mohammad Hassan Alhashmi, Arusha Ayub, Fahad A. Al‐Abbasi, Ajoy Kumer, Shams Tabrez

**Affiliations:** ^1^ Department of Biochemistry Faculty of Science King Abdulaziz University Jeddah Saudi Arabia; ^2^ King Fahd Medical Research Center King Abdulaziz University Jeddah Saudi Arabia; ^3^ Department of Biochemistry Faculty of Life Science Aligarh Muslim University Aligarh India; ^4^ Department of Medical Laboratory Sciences Faculty of Applied Medical Sciences King Abdulaziz University Jeddah Saudi Arabia; ^5^ Department of Medicine College of Health Sciences University of Georgia Georgia USA; ^6^ Department of Chemistry College of Arts and Sciences International University of Business Agriculture & Technology (IUBAT) Dhaka Bangladesh

**Keywords:** cancer, IL‐6 pathway, JAK/STAT signaling, nanomaterial‐based drug delivery, STAT3, targeted therapy

## Abstract

Signal transducer and activator of transcription 3 (STAT3) is a critical transcription factor involved in multiple physiological and pathological processes. While STAT3 plays an essential role in homeostasis, its persistent activation has been implicated in the pathogenesis of various diseases, particularly cancer, bone‐related diseases, autoimmune disorders, inflammatory diseases, cardiovascular diseases, and neurodegenerative conditions. The interleukin‐6/Janus kinase (JAK)/STAT3 signaling axis is central to STAT3 activation, influencing tumor microenvironment remodeling, angiogenesis, immune evasion, and therapy resistance. Despite extensive research, the precise mechanisms underlying dysregulated STAT3 signaling in disease progression remain incompletely understood, and no United States Food and Drug Administration (USFDA)‐approved direct STAT3 inhibitors currently exist. This review provides a comprehensive evaluation of STAT3's role in health and disease, emphasizing its involvement in cancer stem cell maintenance, metastasis, inflammation, and drug resistance. We systematically discuss therapeutic strategies, including JAK inhibitors (tofacitinib, ruxolitinib), Src Homology 2 domain inhibitors (S3I‐201, STATTIC), antisense oligonucleotides (AZD9150), and nanomedicine‐based drug delivery systems, which enhance specificity and bioavailability while reducing toxicity. By integrating molecular mechanisms, disease pathology, and emerging therapeutic interventions, this review fills a critical knowledge gap in STAT3‐targeted therapy. Our insights into STAT3 signaling crosstalk, epigenetic regulation, and resistance mechanisms offer a foundation for developing next‐generation STAT3 inhibitors with greater clinical efficacy and translational potential.

## Graphical abstract

An image depicting the role of STAT3 in various human diseases

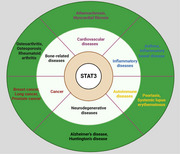

## 1 Introduction

Signal transducer and activator of transcription (STATs) were first identified in 1988 as proteins that bind to interferon (IFN)‐stimulated response elements in DNA sequences and facilitate the transcription of type I IFNs. The Janus kinase/signal transducer and activator of transcription (JAK/STAT) pathway was designated following its discovery in three independent laboratories in 1992. The JAK/STAT signaling pathway function as a crucial regulatory network for various cellular processes. This pathway mediates diverse downstream processes, including apoptosis, tissue repair, inflammation, hematopoiesis, immune regulation, and adipogenesis [1, 2, 3]. STAT3, a member of the STATs family, was identified in 1994 and has been implicated in multiple biological processes, such as wound healing, immune response, tissue regeneration, carcinogenesis, cancer stem cell (CSC) regulation, and cell proliferation and differentiation [4].

STAT3 functions as a central regulator where multiple signaling pathways are activated by various molecules, including specific cytokines, peptide ligands, growth factors, and oncogenes [5, 6]. These proteins undergo activation through tyrosine phosphorylation in response to cytokine signals within the cytoplasm. Once activated, STATs translocate into the cell nucleus, where they bind to specific DNA sequences and function as transcription factors [7]. This transcription factor plays a critical role in numerous biological processes, including angiogenesis, cell proliferation, cell growth, and apoptosis [8, 9]. The activation of STAT proteins enhances the transcription of multiple target genes, leading to processes such as angiogenesis, antiapoptotic responses, and uncontrolled cell division [10]. The human body contains six STAT family members: STAT‐1, STAT‐2, STAT3, STAT‐4, STAT‐5A, STAT‐5B, and STAT‐6, each comprising 750–850 amino acids and playing a vital role in cytokine signaling [10].

Among them, STAT3, particularly through the interleukin‐6 (IL‐6)/JAK/STAT3 axis, has been extensively implicated in both physiological and pathological conditions [7]. Over the past two decades, research has linked persistent STAT3 activation to a wide range of diseases, including bone‐related diseases [11], cardiovascular diseases [12], inflammatory diseases [13], autoimmune disorders [14], neurodegenerative diseases [15], and various cancers [16]. Given its widespread role in disease progression, STAT3 has emerged as a promising therapeutic target for drug development. However, despite significant research efforts, no United States Food and Drug Administration (US FDA)‐approved direct STAT3 inhibitors currently exist, highlighting a critical gap in translating preclinical findings into clinical applications [16].

While several reviews have explored STAT3's role in individual diseases, a comprehensive review integrating its function in both health and disease is still lacking. Most studies focus on either molecular mechanisms or therapeutic strategies yet fail to bridge the gap between fundamental biology and clinical applications. Additionally, STAT3 activation is highly complex, involving crosstalk with multiple signaling pathways, which contributes to immune evasion and drug resistance [9, 17]. With advancements in targeted therapies, immunotherapies, and nanomedicine‐based drug delivery, an updated review consolidating recent discoveries, therapeutic advancements, and future directions is essential for both researchers and clinicians.

This review aims to fill this gap by providing a systematic and in‐depth discussion of STAT3's dual role in health and disease, with a particular focus on oncogenesis and targeted therapy. We explore mechanistic insights into STAT3 signaling, including canonical and noncanonical pathways [18], epigenetic modifications [19, 20], and its role in tumor progression, metastasis, and therapy resistance [16]. Additionally, we analyze emerging therapeutic approaches, such as JAK inhibitors (tofacitinib, ruxolitinib) [21, 22], Src Homology 2 (SH2) domain inhibitors (S3I‐201, STATTIC) [23, 24], monoclonal antibodies (siltuximab, tocilizumab), antisense oligonucleotides (AZD9150) [25], and innovative nanomedicine‐based drug delivery strategies [26]. By evaluating both preclinical and clinical studies, this review seeks to highlight the current challenges and future opportunities in STAT3‐targeted therapies.

To ensure clarity and coherence, this review is structured systematically. It begins with a detailed overview of STAT3's molecular structure, activation mechanisms, and biological functions [27]. We then explore STAT3's involvement in multiple diseases, with a strong focus on cancer development and progression [16]. The latter sections highlight therapeutic approaches, including direct STAT3 inhibitors, immunotherapy‐based interventions, and nanomedicine‐enhanced drug delivery systems (DDSs) [27]. Finally, we discuss current limitations, potential biomarkers for patient stratification, and future research directions. By providing a comprehensive and translational perspective, this review serves as a valuable resource for researchers and clinicians working toward the development of next‐generation STAT3‐targeted therapies with improved clinical efficacy and specificity.

## 2 Overview of STAT3

**FIGURE 1:**
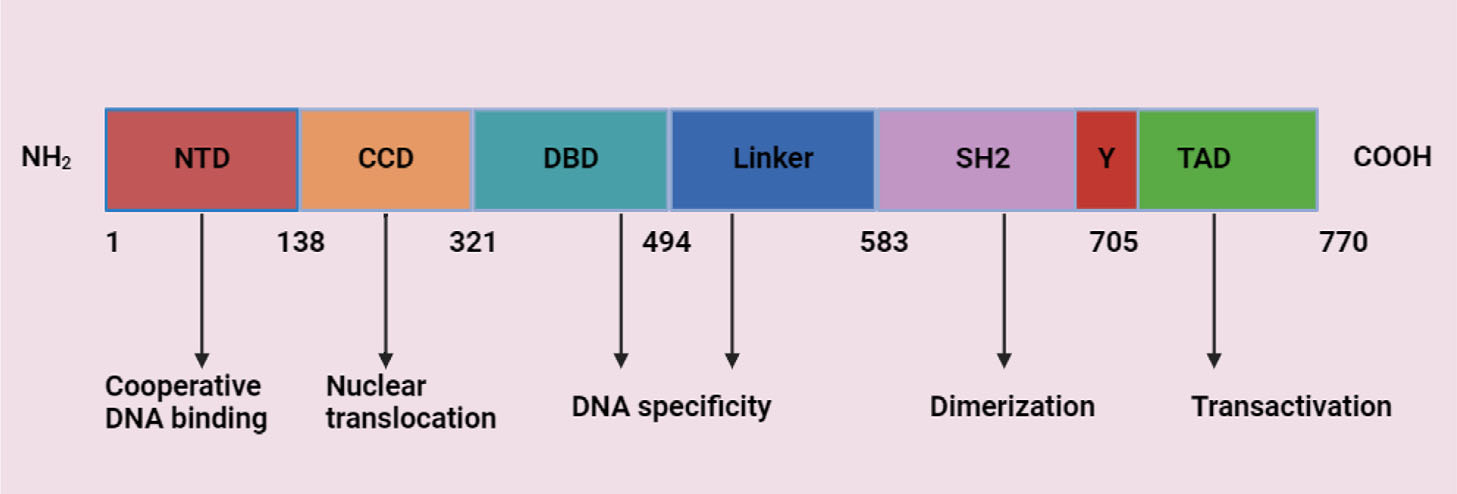
Domain organization and function of STAT3. The STAT3 protein consists of six primary domains, each serving a distinct function. The N‐terminal domain (NTD) contributes to structural stability, while the coiled‐coil domain (CCD) facilitates dimerization between two STAT3 molecules. The DNA‐binding domain (DBD) enables STAT3 to interact with DNA and regulate gene expression. The linker domain connects the DBD to adjacent domains, ensuring functional integrity and flexibility. The Src‐homology 2 (SH2) domain binds to phosphorylated tyrosine residues on other proteins, playing a critical role in STAT3 activation. The C‐terminal or transactivation domain (TAD) promotes the transcription of target genes once STAT3 is bound to DNA. The coordinated interaction of these domains allows STAT3 to regulate various cellular processes, including immune responses and cell proliferation. Created with Biorender.com.

The STAT3 gene is located on chromosome 17 at position 21 on the long arm (17q21). This gene encodes a protein with an approximate molecular mass of 92 kDa, consisting of 770 amino acids. The protein structure comprises several distinct domains, including the N‐terminal domain, the coiled‐coil domain (CCD), the DNA‐binding domain (DBD), the SH2 domain, and the C‐terminal domain, also known as the transactivation domain (TAD). The primary activation sites for STAT3 are the tyrosine and serine residues at positions 705 and 727, respectively, within the C‐terminal region. Additionally, an alpha‐helical linker domain, spanning amino acids 500–575, precedes the SH2 domain. The formation of the STAT3 dimer is dependent on a specific SH2 domain that selectively binds to phosphotyrosine motifs, a process essential for the proper regulation of gene expression [9, 17]. Six isoforms of STAT3 have been identified: STAT3α, STAT3β, STAT3γ, STAT3δ, STAT3ε, and STAT3ζ. While these isoforms perform distinct functions, the classical activities of STAT3 are primarily mediated by STAT3α [28, 29]. The STAT3 gene consists of 24 exons, with alternative splicing of exon 23 leading to the generation of STAT3β, a shorter isoform. This splicing event introduces a frameshift, resulting in the substitution of seven amino acids in the TAD of STAT3α, thereby altering its function [30]. STAT3β exhibits tumor‐suppressive properties due to the absence of a specific activation domain present in STAT3α. Additionally, STAT3β plays a role in stabilizing the ternary complex, inhibiting self‐renewal and proliferation, reducing chemotherapy resistance, attenuating invasion, and promoting apoptosis [31]. The STAT3γ and STAT3δ isoforms arise through proteolytic processing, which is associated with granulocyte and neutrophil development [32, 33]. In contrast, STAT3ε and STAT3ζ are newly identified truncated isoforms of acetylated STAT3α. Furthermore, the N‐terminal region of STAT3ε and the C‐terminal region of STAT3ζ, containing different segments of STAT3α, display structural similarities [33]. The domain organization and function of STAT3 are illustrated in Figure 1.

## 3 Role of STAT3 Signaling in the Development of Various Diseases

**FIGURE 2:**
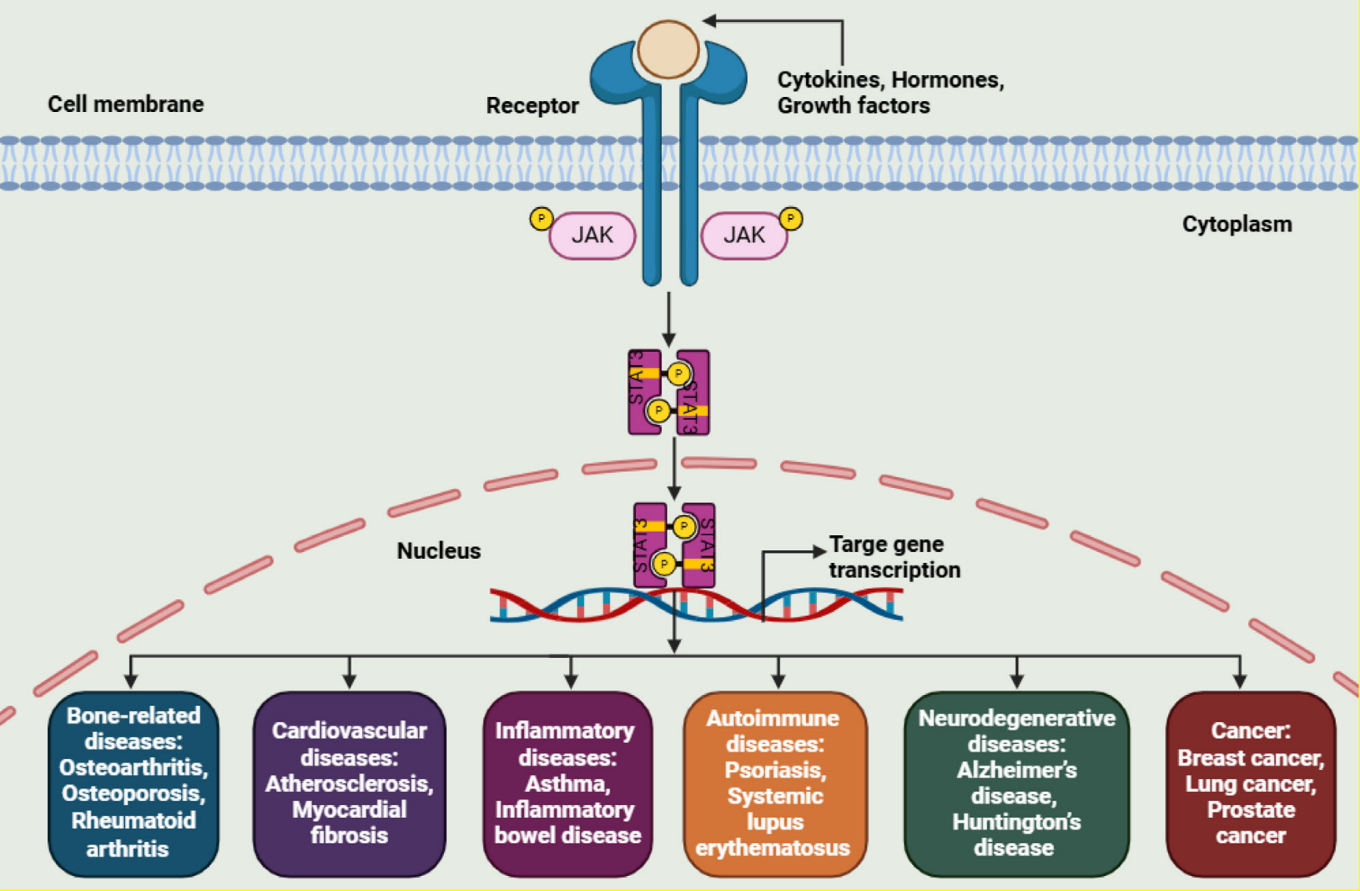
Receptor‐mediated activation of STAT3 signaling contributes to the progression of various diseases, including cancer. Created with Biorender.com.

Aberrant STAT3 signaling has been linked to a broad spectrum of diseases, including bone‐related disorders [11], cardiovascular conditions [12], inflammatory diseases [13], autoimmune disorders, [14] neurodegenerative diseases, [15] and various types of cancer [16]. Figure 2 provides an overview of STAT3 signaling involvement in multiple diseases.

### 3.1 Modulation of STAT3 Signaling in Bone‐Related Diseases

Bone‐related diseases comprise a group of chronic conditions, including osteoarthritis (OA), osteoporosis (OP), rheumatoid arthritis (RA), and various bone abnormalities. These disorders are prevalent among elderly and obese individuals. STAT3 functions as an essential downstream signaling protein for numerous cytokines and plays a vital role in modulating cell proliferation and intercellular interactions within the bone microenvironment [11, 34]. Due to its involvement in immune responses and bone metabolism, STAT3 dysregulation has been associated with several bone‐related disorders [35, 36]. By influencing mesenchymal stem cell development, osteoclast activation, macrophage polarization, angiogenesis, and cartilage degradation, STAT3 directly contributes to the progression of bone‐related diseases [37, 38, 39].

The STAT3 signaling pathway play an essential role in cytogenesis and is implicated in the pathogenesis of OA [40, 41]. Persistent activation of STAT3 can disrupt chondrocyte metabolism, promoting catabolic processes that contribute to joint degradation and the formation of osteophytes or bone spurs, ultimately leading to OA‐associated bone changes [38, 40]. Various in vitro and in vivo investigations have demonstrated that STAT3 activation in chondrocytes, rather than ERK1/2, induces OA, resulting in cartilage degradation and osteophyte formation [42, 43]. Liang et al. [44] identified a positive correlation between retinoic acid receptor‐related orphan receptor α (RORα) expression and OA severity. Furthermore, RORα has been shown to counteract IL‐6‐induced elevations in p‐STAT3, thereby restoring chondrocyte expression of type II collagen (Col‐2) and aggrecan [44]. The synovial membrane secretes substantial amounts of inflammatory cytokines, including IL‐1, IL‐6, TNF‐α, and IL‐8, which diffuse into the cartilage via synovial fluid. This process activates chondrocytes, enhancing the production of additional proinflammatory cytokines, which in turn accelerates degradation of cartilage and progression of OA [45, 46]. The impact of these cytokines contributes to the initiation and advancement of OA through cytokine‐mediated signaling and immune responses [47, 48].

STAT3 plays a crucial role in regulating the inflammatory microenvironment that contributes to OP‐related bone loss. OP is often associated with elevated levels of inflammatory cytokines, particularly IL‐6, which activates STAT3 [49, 50]. In OP, STAT3 modulates osteoclast activity, promoting bone resorption. Excessive osteoclast activation disrupts bone homeostasis, leading to OP, where reduced bone mineral density significantly increases the disability risk and mortality in older individuals [49, 51]. The receptor activator of nuclear factor κB ligand (RANKL) is a crucial mediator in the interactions between osteoclasts and osteoblasts. It plays a vital role in promoting the differentiation and growth of osteoclasts while simultaneously suppressing the osteogenic differentiation of mesenchymal stem cells [52, 53]. RANKL activates the STAT3 pathway, leading to a reduction in tartrate‐resistant acid phosphatase‐positive cells and an increase in the expression of NFATc1, a key osteoclast marker [54, 55]. STAT3 facilitates NFATc1 transcription by directly binding to its promoter. The RANKL–STAT3–NFATc1 axis may play a significant role in RANKL‐induced osteoclast overactivation, further contributing to OP progression [49].

Similarly, STAT3 serves as a key regulator in the development of RA, and inhibiting its activity has been shown to suppress joint inflammation and osteoclast activation [56, 57]. In individuals with RA, inflammatory cytokines such as IL‐6, TNF‐α, and IL‐1 are the primary activators of STAT3, which subsequently promotes IL‐6 expression through a positive feedback loop [58, 59]. Activated STAT3 also upregulates the expression of RANKL, a critical factor involved in osteoclastogenesis [60]. Osteoclasts play a pivotal role in joint destruction associated with RA, contributing to disease progression [61, 62].

### 3.2 Modulation of STAT3 in the Development of Cardiovascular Diseases

Cardiovascular diseases encompass a group of disorders affecting the heart and blood vessels and represent the leading cause of mortality worldwide [63, 64]. STAT3 has been implicated in various cardiovascular conditions, including atherosclerosis [65], myocardial fibrosis [12], and other related disorders [66]. Atherosclerosis serves as the primary pathological foundation for ischemic and cerebrovascular diseases. Activation of the JAK2/STAT3 pathway is strongly linked to the IL‐6 cytokine family, which plays a critical role in endothelial cell dysfunction associated with atherosclerosis [65, 67]. Furthermore, IL‐6 functions as a key proinflammatory cytokine, significantly contributing to STAT3‐mediated inflammation in the progression of atherosclerosis [68].

Upon activation of the JAK2/STAT3 pathway in vascular endothelial cells, IL‐6 has been found to upregulate monocyte chemotactic protein‐1 expression, leading to several proinflammatory effects [69]. In atherosclerotic plaques, IL‐10 is primarily expressed in macrophages. Unlike the IL‐6‐induced STAT3 signaling pathway, which promotes inflammation, the IL‐10/JAK/STAT3 signaling pathway exerts anti‐inflammatory effects in macrophages [70, 71]. Depending on local microenvironmental signals, macrophages differentiate into either a proinflammatory (M1) or anti‐inflammatory (M2) phenotype, playing a role in atherosclerosis progression [72, 73]. The JAK2/STAT3 pathway promotes macrophage polarization toward the M1 phenotype, increasing the production of inflammatory molecules such as tumor necrosis factor‐alpha (TNF‐α), thereby accelerating the development of atherosclerosis [74, 75].

Myocardial fibrosis is characterized by an excessive accumulation of extracellular matrix proteins, primarily collagen, within the myocardium. Research has demonstrated that the JAK/STAT3 pathway plays a crucial role in the cardiac fibrosis process [12, 76]. This pathway can be activated by various profibrotic mediators, including transforming growth factor beta 1 (TGF‐β1), platelet‐derived growth factor (PDGF), vascular endothelial growth factor (VEGF), IL‐6, Ang II, serotonin, and endothelin, ultimately contributing to fibrogenesis [12, 77]. Additionally, the JAK/STAT3 pathway serves as a key integrator of multiple profibrotic signaling cascades, leading to increased fibroblast activation and the upregulation of fibrosis‐associated genes, such as α‐smooth muscle actin, collagens, and fibronectin [78, 79]. Moreover, activated STAT3 can induce epithelial‐to‐mesenchymal transition (EMT), facilitating the transformation of epithelial cells into mesenchymal cells with enhanced migratory and invasive properties, thereby promoting fibrosis progression [80, 81].

### 3.3 Modulation of STAT3 in Inflammatory Diseases

STAT3 is activated by various cytokines, including IL‐6, IL‐10, IL‐17, and TNF‐α, which play a central role in the onset and progression of inflammatory diseases [82, 83, 84]. Persistent or dysregulated activation of STAT3 has been linked to multiple chronic inflammatory conditions, such as asthma [85], inflammatory bowel disease (IBD) [84], and other inflammatory disorders [83].

Asthma is characterized by airway inflammation, leading to increased airway sensitivity and structural remodeling of the airway wall. STAT3 activation, along with elevated Th2 and Th17 cytokine level in the lungs, has been associated with airway inflammation and remodeling [86, 87]. During allergic inflammation, STAT3 regulates the recruitment of immune cells, particularly Th2 cells, and contributes to the production of Th17 cells [88]. Th17 cell activation is mediated by cytokines such as TGF‐1, IL‐1, IL‐6, and IL‐23, resulting in increased expression of transcription factors specific to Th17 differentiation, including RORγ and RORα [85, 89]. Dysregulated production of IL‐17 results in the generation of proinflammatory cytokines and chemokines, which subsequently recruit inflammatory cells to the affected site [90]. Several studies have indicated that prolonged IL‐17 activation contributes to enhanced deposition of collagen, increased mass of airway smooth muscle, and enlarged mucous glands [91, 92].

IBD encompasses a group of chronic disorders affecting the colon and small intestine. STAT3 plays a critical role in inflammation, tissue repair, and immune regulation in IBD [93, 94]. More than 160 genetic loci have been associated with IBD susceptibility, including STAT3‐related genes involved in intestinal mucosal immune responses [95]. The pathophysiology of IBD is characterized by elevated levels of cytokines such as IL‐6, IL‐22, and IL‐23, which serve as ligands for cell surface receptors, leading to STAT3 activation [96, 97]. The effects of STAT3 activation are context‐dependent, influenced by the cellular environment. STAT3 promotes regulatory T cells (Tregs) to modulate excessive immune responses while also facilitating Th17 cell development and survival, thereby contributing to chronic inflammation [98, 99]. Increased STAT3 expression in T‐cells, macrophages, and epithelial cells has been strongly correlated with histological inflammation severity [100]. Additionally, STAT3 activation in T‐cells has been implicated in the pathogenesis of colitis [101, 102].

### 3.4 Modulation of STAT3 in Autoimmune Diseases

STAT3 plays a crucial role in the early development and maturation of B‐cells within the bone marrow. Additionally, it facilitates class‐switch recombination in B‐cells in response to specific cytokines, which is necessary for generating distinct antibody isotypes [103, 104, 105]. STAT3 also regulates multiple aspects of natural killer (NK) cell biology, including NK cell development, activation, cytotoxic function, and modulation of innate and adaptive immune responses [106, 107]. Dysregulated or excessive STAT3 signaling has been associated with the onset and progression of various autoimmune diseases, including psoriasis and systemic lupus erythematosus (SLE) [108, 109, 110]. Psoriasis is a chronic autoimmune disorder characterized by red, dry, itchy, and scaly skin patches. It is primarily driven by Th17 lymphocytes, which differentiate from naive T‐cells upon IL‐6 stimulation [111, 112]. Inflammatory interactions between Th1 and Th17 cells and keratinocytes further contribute to psoriasis pathogenesis [113, 114]. The skin of individuals with psoriasis exhibits elevated STAT3 expression [115, 116]. The topical application of a STAT3 inhibitor has been shown to reduce psoriatic lesions in both transgenic mice and clinical patients [117, 118].

SLE is a chronic autoimmune disorder marked by extensive inflammation and autoantibody production [119, 120]. Hyperactivity of immune cells, including T‐cells, B‐cells, and dendritic cells, has been associated with abnormal STAT3 activation in SLE [121, 122]. This dysregulated activation promotes the production of autoantibodies and immune complexes, contributing to tissue damage in the kidneys, skin, joints, and other organs [123]. STAT3 transactivates IL‐10 in T‐cells of SLE patients through epigenetic remodeling [124, 125]. An imbalance between Th17 and Tregs is believed to play a critical role in SLE progression, leading to an enhanced proinflammatory response, particularly during active disease phases [126, 127]. The use of STAT3 inhibitors in combination with immunosuppressive therapies has been shown to improve SLE by restoring this balance, while agents targeting STAT3 phosphorylation have demonstrated efficacy in treatment [109, 128]. Additionally, increased Th17 proliferation and an active IL‐17/STAT3 axis have been implicated in SLE pathogenesis [129].

### 3.5 Modulation of STAT3 in Neurodegenerative Diseases

STAT3 plays a key role in the response to neurotrophic factors, such as nerve growth factor and brain‐derived neurotrophic factor (BDNF), which support neuronal survival and development [130, 131]. Following injury, STAT3 activation in brain astrocytes contributes to scar formation and tissue repair in the central nervous system [132, 133]. Its activation leads to the upregulation of genes involved in neuroprotection, neuroregeneration, and neurodevelopment [134].

Alzheimer's disease (AD) is the primary contributor of dementia in elderly populations [135, 136]. STAT3 has been implicated in neuroinflammation and the progression of AD [137]. The JAK2–STAT3 signaling pathway influences astrocytes, hippocampal neurons, and microglia, contributing to disease pathology [138, 139]. Additionally, STAT3 has been shown to impact A‐β42, beta‐site APP cleaving enzyme 1 (BACE1), tau tangles, and other key components in the AD brain [140, 141].

Huntington's disease (HD) is a fatal neurodegenerative disorder characterized by striatal neurodegeneration, the accumulation of mutant huntingtin (mHTT), and the presence of reactive astrocytes. STAT3 activation plays a crucial role in regulating inflammatory responses in glial cells, particularly astrocytes, and microglia, which are essential for maintaining brain homeostasis [142, 143]. Persistent STAT3 activation in HD has been associated with increased neuroinflammation, a hallmark of the disease [131, 144]. The JAK2–STAT3 pathway regulates astrocyte reactivity and has been found to be activated in the putamen of individuals with HD [145]. Moreover, inhibition of the JAK2–STAT3 pathway in reactive astrocytes has been shown to reduce their reactive characteristics while increasing the accumulation of mHTT aggregates [146, 147].

### 3.6 Modulation of STAT3 in Different Cancer Types

Cancer remains one of the leading causes of mortality worldwide, with its incidence and prevalence increasing annually as the population ages [148, 149]. Several key signaling pathways, including the mammalian target of rapamycin, Wnt, STAT3, mitogen‐activated protein kinase (MAPK), and phosphoinositide 3‐kinase, play essential roles in cell growth, survival, and proliferation, and their dysregulation is a hallmark of cancer [150]. STAT3, in particular, has been strongly linked to the progression of multiple cancer types, including malignancies of the head and neck, lungs, stomach, liver, colon, prostate, and breast, where it facilitates cancer cell proliferation, invasion, and metastasis [151, 152]. Research indicates that STAT3 influences DNA modification and chromatin remodeling in the nucleus through epigenetic mechanisms [5, 153]. Additionally, STAT3 contributes to the regulation of immune responses within various tumor microenvironments (TMEs). The existing scientific literature underscores the critical role of STAT3 in multiple human diseases, particularly cancer. The following sections will examine the involvement of STAT3 in cancer progression and explore its potential as a therapeutic target, highlighting both the opportunities and challenges associated with these approaches.

## 4 The Canonical and Noncanonical STAT3 Signaling Pathways in Cancer

The cytoplasm contains an inactive monomeric STAT3 unit under normal conditions. Activation of STAT3 requires the phosphorylation of tyrosine 705, a critical step in the canonical STAT3 activation process. Once activated, STAT3 interacts with various receptors stimulated by cytokines and growth factors [18]. Following activation, two monomeric STAT3 units associate via the tyrosine 705 residue, forming either homodimers (composed of two STAT3 molecules) or heterodimers (a STAT3 molecule paired with another STAT protein). Upon nuclear translocation, these dimers regulate the expression of genes involved in multiple cellular functions, including cell division maintenance [154], metastasis promotion [16], angiogenesis facilitation [155], inflammation induction [156], apoptosis inhibition [157], immune response suppression [158], TME modulation [159], CSC maintenance [158], metabolic alterations [154], drug resistance development [160], and the mediation of cancer hallmark activities through exosomes [161]. This regulation occurs upon binding to the SH2 domain. In addition to tyrosine 705, serine 727 represents another critical functional site within STAT3. Serine and threonine kinases facilitate the phosphorylation of serine 727, thereby contributing to STAT3 activity [162]. STAT3 regulation is primarily controlled by protein tyrosine phosphatases (PTPases), which dephosphorylate STAT3, modulating its function. Furthermore, suppressor of cytokine signaling 3 (SOCS3) inhibits STAT3 at the receptor level, whereas the protein inhibitor of activated STATs 3 (PIAS3) suppresses STAT3 function at the gene transcription stage [70].

The canonical signaling pathway involves STAT3 activation through the phosphorylation of tyrosine 705. Research has demonstrated that STAT3 can shuttle between the cytoplasm and nucleus, influencing cellular processes even in its nonphosphorylated state [18]. Kinases such as MAPK, JNK1/2, GSK3α/3β, and CDKs phosphorylate STAT3 at serine 727, enhancing its mitochondrial functions without requiring nuclear localization. GRIM‐19 facilitates the import of phosphorylated serine STAT3 (P‐Ser‐STAT3) into mitochondria, where it regulates electron transport chain complexes I, II, and V. Mitochondrial STAT3 (mtSTAT3) contributes to ATP production, reduces reactive oxygen species release, and enhances mitochondrial calcium uptake and mitochondrial permeability transition pore regulation. In addition to phosphorylation, STAT3 undergoes acetylation at lysine 685 (K685Ac), a modification that influences its stability, activity, and interactions with other proteins, thereby broadening its functional repertoire [33, 134, 163].

**FIGURE 3:**
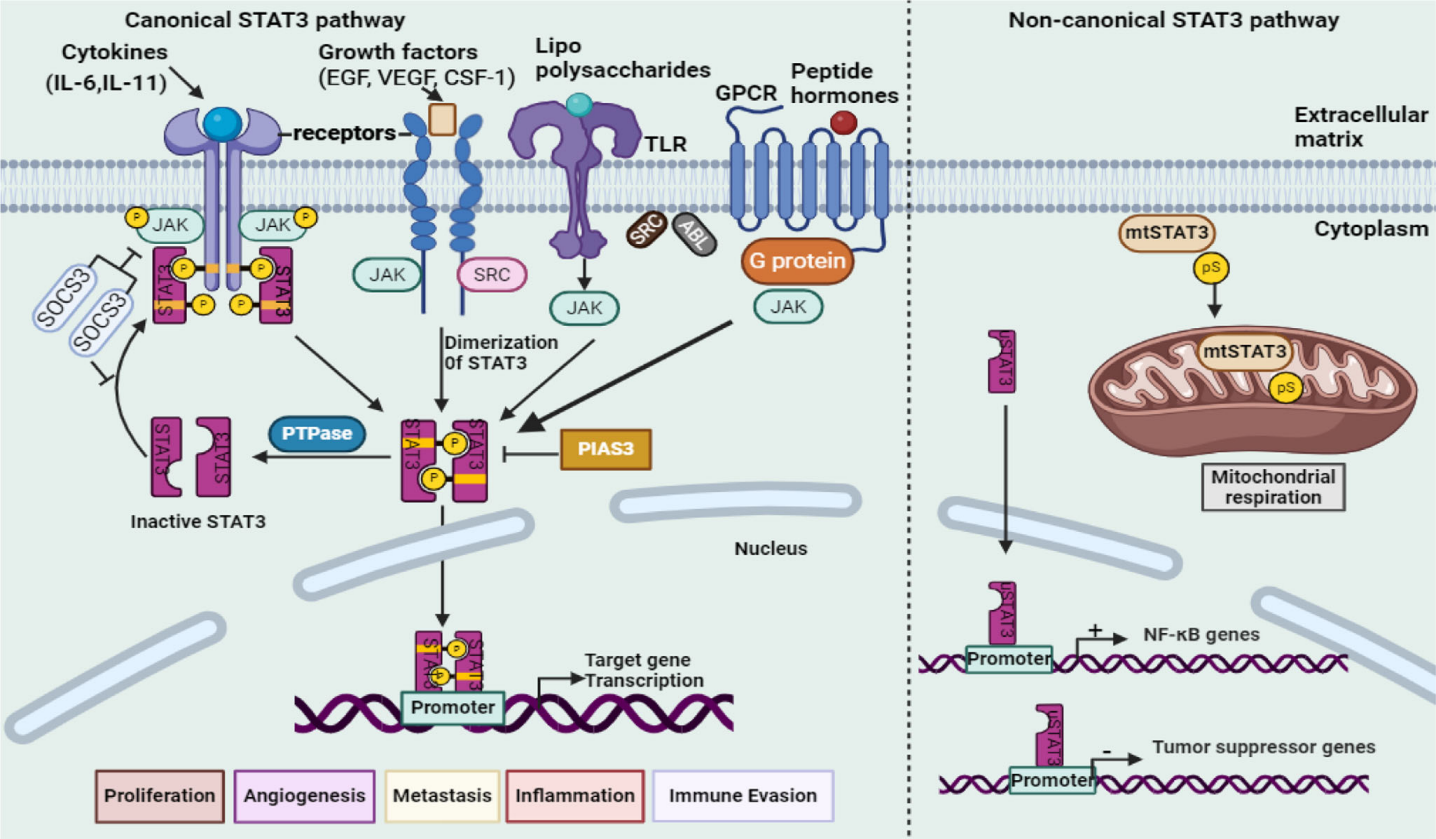
The canonical STAT3 signaling pathway is activated by multiple receptors, including interleukin‐6 (IL‐6) receptors, IL‐6 family cytokine receptors (such as IL‐11 and IL‐23 receptors), growth factor receptors, G protein‐coupled receptors (GPCRs), and Toll‐like receptors (TLRs). These receptors are stimulated by various ligands, including lipopolysaccharide (LPS), cytokines, sphingosine‐1‐phosphate, angiotensin, and hormones. Most of these receptors lack intrinsic kinase activity. Upon ligand binding, conformational changes occur, leading to the activation of Janus kinases (JAKs), which serve as docking sites for STAT3 via the SH2 domain. Additionally, oncoproteins with tyrosine kinase activity, such as SRC and BCR‐ABL, constitutively activate STAT3. Phosphorylated STAT3 molecules form dimers when the phosphorylated tyrosine residue at position 705 on one STAT3 molecule interacts with the SH2 domain of another STAT3 molecule. These dimers translocate into the nucleus, where they bind to DNA response elements within the promoter regions of target genes. These genes regulate various biological processes, including angiogenesis, cancer metastasis, immune suppression within the tumor microenvironment, metabolic alterations, exosome‐mediated signaling, cancer stem cell properties, and drug resistance. The noncanonical STAT3 signaling pathway exists in three distinct forms: Tyr705–phosphorylated STAT3 (alone or in combination with p‐STAT3 Ser727, mtSTAT3, and unphosphorylated STAT3). These variants modulate nuclear factor kappa B (NF‐κB), mitochondrial respiration, and other gene functions, some of which remain to be fully elucidated. Created with Biorender.com. EGF, epidermal growth factor; VEGF, vascular endothelial growth factor; CSF1, colony‐stimulating factor 1; mtSTAT3, mitochondrial signal transducer and activator of transcription 3; NF‐κB, nuclear factor kappa B.

The noncanonical STAT3 pathway is characterized by β‐catenin‐independent mechanisms, encompassing intracellular signaling and target gene regulation. It is implicated in biological processes such as tissue repair, immune regulation, tumor progression, and metabolic adaptation [152, 164]. In the cytoplasm, STAT3 modulates signaling pathways through interactions with proteins such as NF‐κB, thereby influencing survival and inflammatory responses. It also regulates microtubule dynamics and cytoskeletal organization, facilitating metastasis and cell migration [165]. Nonphosphorylated STAT3 dimers may participate in epigenetic regulation by directly or indirectly modulating gene expression through interactions with chromatin modifiers [18]. Additionally, in its unphosphorylated form, where phenylalanine replaces tyrosine 705, STAT3 regulates NF‐κB transcription and genes associated with EMT by interacting with either unphosphorylated NF‐κB or Jun activation domain‐binding protein 1, contributing to tumor suppression [33]. Figure 3 illustrates the mechanism of action of canonical and noncanonical STAT3 signaling pathways.

## 5 Various Roles of STAT3 Signaling in Cancer

### 5.1 Oncogenic Role of STAT3 Signaling

Oncogenes such as K‐ras and src are specialized genes capable of transforming normal cells into cancerous cells upon activation [166, 167]. These oncogenes may be introduced into cells through viral infection or arise from mutations in normal genes. Persistent STAT3 activation has been observed in breast cancer [168], suggesting a direct association with oncogenic signaling. Cells infected with Epstein–Barr virus and human T‐lymphotropic virus 1 exhibit continuous STAT3 activity due to increased tyrosine kinase activity [169]. STAT3 functions as a central regulator in several well‐established oncogenic pathways and plays a key role in activating Toll‐like receptors (TLRs), G protein‐coupled receptors (GPCRs), fibroblast growth factor (FGF), insulin‐like growth factor, IL‐6, IL‐11, IL‐10, and IFN‐α [170]. By activating multiple downstream targets, angiotensin II receptor (AgtR2) and IFN contribute to the regulation of diverse cellular processes. These include cyclin D1, which governs the cell cycle; c‐Myc, which promotes cell proliferation; matrix metalloproteinases (MMP)‐2 and MMP‐9, which facilitate tissue remodeling; and VEGF, which supports angiogenesis. Additionally, STAT3 influences cyclooxygenase‐2 (COX‐2), involved in inflammation; survivin, which inhibits apoptosis; and programmed cell death ligand 1 (PD‐L1), which modulates immune responses [171, 172, 173]. Research has demonstrated that STAT3 remains persistently activated in v‐Src‐transformed cells, suggesting its potential as a therapeutic target in cancer. The introduction of a constitutively active STAT3 variant has been shown to be necessary for transforming immortalized fibroblasts and normal epithelial cell lines derived from the breast or prostate [7, 174]. These findings indicate that aberrant STAT3 activation may lead to persistent alterations in gene expression patterns, ultimately contributing to malignant transformation.

Inflammatory cytokines such as IL‐6 play a key role in immune responses and can be produced following STAT3 activation. Activated STAT3 induces the expression of angiogenic proteins, including VEGF and hypoxia‐inducible factor 1‐alpha (HIF‐1α), which are critical for new blood vessel formation. These processes contribute to a tumor‐supportive microenvironment that facilitates cancer progression and metastasis [175]. Constitutive activation of STAT3 has been observed in both solid tumors and hematological malignancies, such as leukemia and lymphoma, and serves as a prognostic marker for disease progression [16, 33]. In gastric cancer, elevated levels of phosphorylated STAT3 (pY705 STAT3) have been associated with reduced overall survival [176, 177]. Similarly, ovarian and prostate cancers exhibit increased STAT3 activity [160, 178]. In colorectal cancer, heightened STAT3 expression correlates with tumor invasion, lymph node metastasis, and tumor progression [179, 180]. Additionally, elevated STAT3 levels have been linked to poor clinical outcomes in several malignancies, including cervical cancer [181], esophageal squamous cell carcinoma [182], and squamous cell carcinoma of the head and neck (HNSCC) [183]. These findings highlight the critical role of activated STAT3 in oncogenesis, supporting the potential of STAT3 suppression as a therapeutic strategy for specific cancer types.

The STAT3C construct has shown to enhance tumor growth in various cell types by upregulating key factors such as MMP‐9, VEGF, and C‐terminal Tensin‐like [184, 185]. Elevated STAT3 expression contributes to tumor development and progression by inhibiting apoptosis in cancer cells [6, 186]. A study analyzing tissue samples from breast cancer patients, both with and without lymph node metastasis, reported a correlation between increased levels of phosphorylated STAT3 (phospho‐STAT3) and improved short‐ and long‐term survival rates [187]. According to Pascal et al. [188], STAT3 regulates the ARF–MDM2–p53 pathway, where ARF inhibits MDM2, a protein responsible for targeting p53 for ubiquitination and proteasome‐mediated degradation. This suggests that STAT3 may influence the tumor‐suppressive function of p53. A reduction in STAT3 signaling in a prostate cancer mouse model has been associated with an increased likelihood of metastasis and disease recurrence. These findings indicate that inhibiting the IL‐6/STAT3 pathway may not be a viable therapeutic approach for prostate cancer and could potentially result in poorer clinical outcomes [188].

### 5.2 | Activation and Regulation of STAT3

**FIGURE 4:**
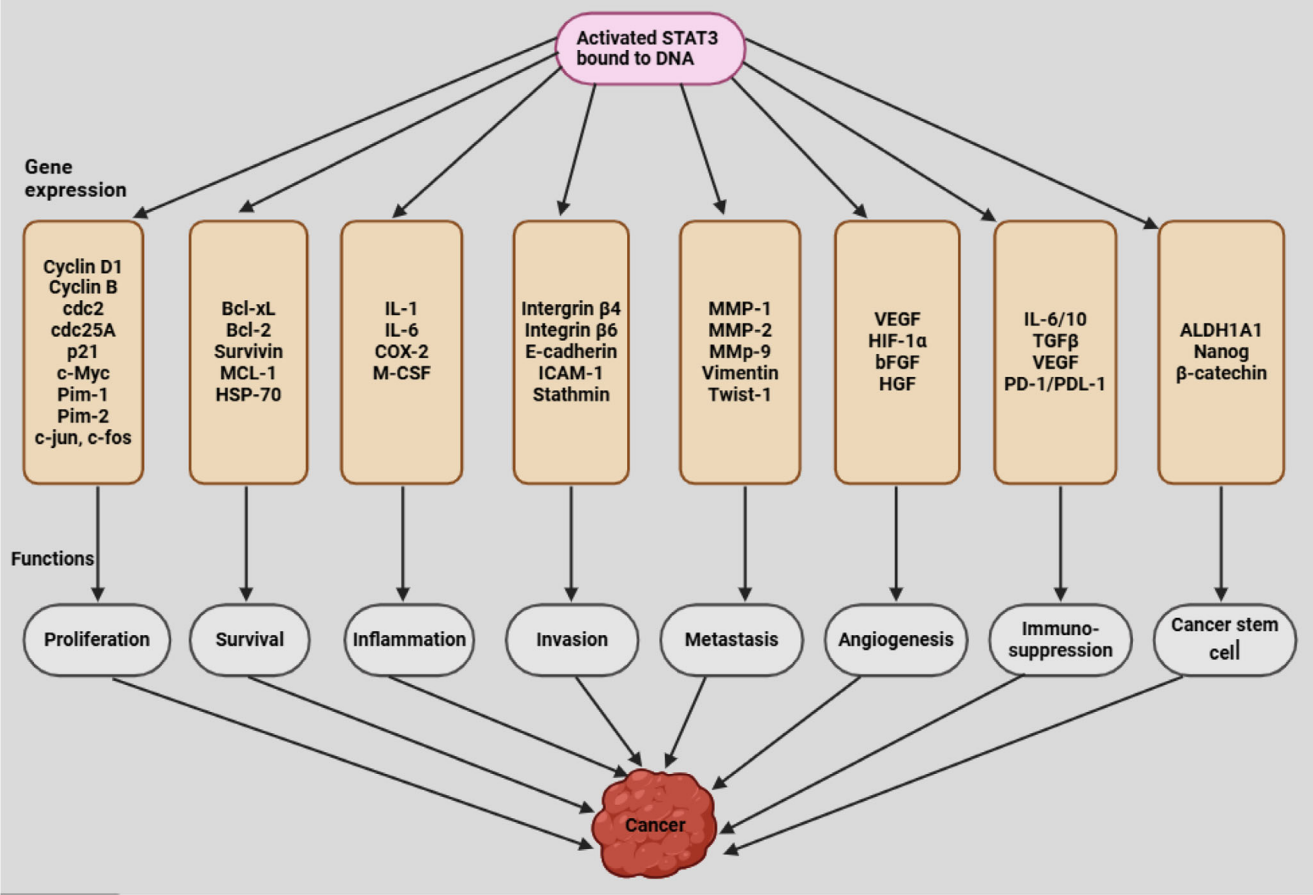
The binding of activated STAT3 to DNA leads to alterations in the expression of genes associated with proliferation, inflammation, survival, angiogenesis, invasion, and immune suppression, ultimately contributing to cancer development. Created with Biorender.com. cdc2, cell division control 2; c‐myc, cellular myelocytomatosis oncogene; Pim‐1/2, proto‐oncogene serine/threonine‐protein kinase 1/2; Bcl‐xL, B‐cell lymphoma‐extra‐large; MCL‐1, myeloid cell leukemia sequence‐1; HSP‐70, heat shock protein 70; COX‐2, cyclooxygenase‐2; M‐CSF, macrophage colony‐stimulating factor; ICAM‐1, intercellular adhesion molecule 1; VEGF, vascular endothelial growth factor; HIF‐1α, hypoxia‐inducible factor‐1; bFGF, basic fibroblast growth factor; HGF, hepatocyte growth factor; TGFβ, transforming growth factor beta; ALDH1A1, aldehyde dehydrogenase 1A1.

STAT3 regulates the initiation and progression of human cancer through multiple mechanisms [153, 160]. Activation of STAT3 by tyrosine kinase inhibitors, a class of cancer therapeutics, has been associated with the development of resistance to these treatments [189, 190]. Persistent STAT3 activation enhances the production of antiapoptotic proteins such as survivin, MCL‐1, Bcl‐2, and Bcl‐xL while simultaneously downregulating the Fas signaling pathway, which plays a role in programmed cell death [9, 172, 191]. STAT3 directly interacts with the promoters of MMPs, including MMP‐1, MMP‐2, MMP‐7, and MMP‐9, leading to increased expression of these proteins in various aggressive malignancies [16]. Additionally, STAT3 promotes the generation of CSCs and facilitates EMT by upregulating transcription factors associated with EMT, such as N‐cadherin, TWIST, ZEB1/2, Snail, and Vimentin, while suppressing E‐cadherin, a key protein involved in cell‐cell adhesion [192]. Emerging evidence suggests that STAT3 plays a crucial role in modulating immune responses related to tumor development and immune suppression [9, 165]. In lymphoma‐associated macrophages, STAT3 regulates the expression of immune checkpoint proteins, including programmed death‐1 (PD‐1) and programmed death‐ligand 1 (PD‐L1), contributing to immune evasion in cancer [171]. Figure 4 demonstrates STAT3 activation leading to cancer development.

#### 5.2.1 Regulation of STAT3 Signaling

Under normal physiological conditions, STAT3 is tightly regulated and functions as both an oncogene and a transcription activator. Scientific studies have indicated that STAT3 remains persistently activated in various cancers and plays a crucial role in tumor initiation and progression [164, 186]. STAT3 influences gene expression through epigenetic modulation. Acetylated STAT3 has been shown to facilitate DNA methylation‐mediated silencing of tumor suppressor genes, while unphosphorylated STAT3 contributes to chromatin organization [19, 20]. Despite its association with multiple regulatory mechanisms and its significance in numerous biological processes, effective therapeutic strategies for STAT3 inhibition in clinical applications have not yet been fully developed. Further investigation into the complex roles of STAT3 in different cancer types is essential for the development of successful therapies targeting the STAT3 signaling pathway.

##### 5.2.1.1 Positive Regulators of STAT3

Phosphorylation of the pY705 residue occurs through multiple pathways that activate the STAT3 signaling cascade. These pathways include nonreceptor tyrosine kinases (nRTKs), such as Src and Abl kinases, cytokine receptors that activate JAKs, and receptor tyrosine kinases (RTKs), including epidermal growth factor receptor (EGFR) and PDGF receptor [193, 194]. STAT3 undergoes phosphorylation at both S727 and Y705 residues, with kinases such as cyclin‐dependent kinase 5 (CDK5) and MAPK mediating S727 phosphorylation. Additionally, phosphorylation at both pY705 and pS727 is required for complete STAT3 activation [195, 196]. Acetylation of STAT3 at the K685 residue enhances its dimerization capacity and increases transcriptional activity, thereby strengthening its role in gene regulation [19, 197]. A key mechanism through which STAT3 exerts its effects involves the secretion of growth factors and cytokines by the TME. This persistent stimulation of STAT3 occurs through paracrine (between adjacent cells) or autocrine (within the same cell) signaling mechanisms [198] Continuous exposure to regulatory molecules within the TME leads to sustained STAT3 activation. Activators such as IL‐6, IFN‐γ, and EGF stimulate the JAK/STAT pathway, which is critical for tumor progression and metastasis [199]. IL‐6, a cytokine widely distributed in the TME, functions as a mediator of both pro‐ and anti‐inflammatory responses. Upon binding to the IL‐6 receptor (IL‐6R) on cell membranes, IL‐6 forms a complex with gp130 or IL‐6Rβ, triggering STAT3 activation and promoting tumor development [200]. In the trans‐signaling pathway, the soluble form of IL‐6 receptor (sIL‐6R) binds to IL‐6, and the IL‐6/sIL‐6R complex subsequently interacts with gp130 to facilitate signaling [201]. Furthermore, activated STAT3 can enhance IL‐6 expression, establishing a positive feedback loop that sustains STAT3 hyperactivation. In prostate cancer, IL‐6‐induced STAT3 activation accelerates tumor progression, a condition clinically identified as neuroendocrine‐differentiated prostate cancer [202].

JAK1, JAK2, JAK3, and TYK2 are nRTKs belonging to the JAK family. These enzymes play a crucial role in transmitting signals from various cytokine receptors to the intracellular environment, where they regulate cell development and immune responses. TYK2, JAK1, and JAK2 are expressed in multiple cell types, whereas JAK3 expression is predominantly restricted to hematopoietic cells. Upon interaction with gp130, JAK activation occurs, leading to the phosphorylation of STAT3 [203]. Additionally, mutations in nRTKs contribute to the expression of the oncoprotein BCR–ABL, which has been associated with the development of hematological malignancies, including leukemia, lymphoma, and myeloma, through the STAT3 signaling pathway [204, 205].

Multiple signaling pathways can activate STAT3 through JAK/STAT3 activation, extending beyond the IL‐6/JAK/STAT3 pathway. Receptors such as EGFR, GPCRs, CXCR, FGF receptor, and B7‐H3 initiate these signaling cascades. Exposure to carcinogens that phosphorylate STAT3 can also trigger these pathways [206, 207]. Protein tyrosine kinases (PTKs), including Lyn, Fyn, Hck, Src, Lck, and Fgr, contribute to STAT3 activation. Furthermore, in the absence of JAK, viral Src induces constitutive STAT3 activation. Cellular Src tyrosine kinase, activated by ligands of the human EGFR family and PDGF, positively regulates STAT3 activity, enhancing its responsiveness to growth factors [27]. EGFR, frequently overexpressed in epithelial malignancies, promotes cancer cell survival and progression. It directly interacts with active STAT3 and phosphorylates it, thereby increasing its activity. Targeting both STAT3 and EGFR has been shown to disrupt the feedback loop between these proteins and inhibit pancreatic cancer progression [190]. Additionally, two well‐characterized GPCRs, sphingosine‐1‐phosphate receptor and AgtR2, activate STAT3 via JAKs [20]. TLRs, including TLR2, TLR3, TLR4, TLR7, and TLR9, are expressed in various immune and epithelial cells, as well as in stromal compartments. These receptors play a critical role in regulating immune responses and influencing cancer progression [33]. TLR stimulation directly activates STAT3 during human B‐cell IgG production and is crucial for both antibody and IL‐10 production [208]. The classical TLR4 activator, lipopolysaccharide, significantly increases phosphorylated STAT3 levels in human bladder cell line T24, highlighting the involvement of TLR4 signaling in STAT3 activation [209].

Noncoding RNAs, including microRNAs (miRNAs) and long noncoding RNAs (lncRNAs), also regulate STAT3 activity [210]. By interacting with specific mRNA targets, these ncRNAs either promote mRNA degradation or inhibit translation, thereby modulating STAT3 expression [211]. Several lncRNAs, such as HOTAIR, ITIH4‐AS1, GACAT3, NEAT1, and FOXD2‐AS1, have been identified as positive regulators of the STAT3 pathway. Additionally, miRNAs that enhance STAT3 activation include miR‐629, miR‐34a, miR‐149, miR‐495‐3p, and miR‐24 [27, 153]. Conversely, STAT3 can also be indirectly activated by miRNAs such as miR‐182‐5p, miR‐203, miR‐221‐3p, and miR‐4449. This activation occurs through the suppression of members of the SOCS family and PIAS [27, 33].

##### 5.2.1.2 Negative Regulators of STAT3

To prevent excessive activation, several regulators tightly control STAT3 signaling in normal tissues, maintaining a balanced state. However, tumor cells often suppress these negative regulators, allowing sustained STAT3 activation. Targeting these regulators, either by directly inhibiting STAT3 or by disrupting the STAT3 signaling pathway, presents potential therapeutic strategies for cancer treatment.

Tyrosine phosphatases play a crucial role in the negative regulation of STAT3 through dephosphorylation [212]. The PTP family, which includes T‐cell PTP (TC‐PTP), SH2‐domain‐containing PTP1 (SHP1), SHP2, PTP‐nonreceptor type 9 (PTPN9), PTP receptor‐type D (PTPRD), PTP receptor‐type T (PTPRT), and PTP receptor‐type K (PTPRK), is essential for regulating the JAK–STAT3 pathway by dephosphorylating STAT3 [18, 32]. Reduced PTPRD expression has been reported in nasopharyngeal carcinoma (NPC), whereas increased PTPRD levels enhance the sensitivity of NPC cells to radiotherapy by decreasing STAT3 phosphorylation [213]. Similarly, reduced PTPRT expression is associated with elevated phosphorylated STAT3 levels and increased susceptibility to STAT3 inhibition in HNSCC [213]. PTPRK has been shown to suppress tumor growth by inhibiting EGFR signaling [214]. Additionally, in triple‐negative primary breast cancer, TC‐PTP deficiency enhances cell proliferation by strengthening Src family kinase (SFK) and STAT3 signaling pathways [215, 216].

The SOCS protein family consists of cytokine‐inducible SH2‐containing protein and SOCS1‐7, which inhibit STAT3 activation through distinct mechanisms. These proteins can directly bind to specific regions of the JAK protein or interact with JAK‐activated cytokine receptors, thereby preventing STAT3 activation [217]. Yoshikawa et al. [218] reported that silencing SOCS1 led to persistent activation of the JAK2/STAT3 pathway in liver cancer cells. Restoring SOCS1 function suppressed cell proliferation in a manner similar to AG490, a JAK2 inhibitor, indicating that SOCS1 plays a crucial role in the negative regulation of the JAK/STAT3 pathway [218]. Additionally, SOCS1 suppresses the expression of CDK2 and 4, along with cell cycle regulatory proteins such as cyclin D1 and cyclin E, thereby inhibiting prostate cancer growth and metastasis [27]. A study demonstrated that SOCS1 and SOCS3 facilitate myogenic differentiation by inhibiting JAK1 and gp130 signaling [219]. Reduced expression or mutations in SOCS1 and SOCS3 have been associated with sustained STAT3 activation, accelerating the progression of pancreatic ductal adenocarcinoma [220], prostate cancer [221], and glioblastoma [222].

A distinct class of proteins, known as PIAS, regulates STAT3 activity within the nucleus. Four PIAS genes—PIAS1, PIAS2, PIAS3, and PIAS4—have been identified in mammals. These proteins inhibit STAT3 function by binding to activated STAT dimers, thereby preventing their interaction with DNA and subsequent gene expression modulation [223]. Among the PIAS family, PIAS3 serves as a key negative regulator. Several studies have demonstrated that elevated PIAS3 expression can suppress cell proliferation and enhance tumor sensitivity to specific therapeutic agents [224]. Jiang et al. [225] reported that excessive activation of the JAK/STAT pathway, due to SOCS3 and PIAS3 abnormalities, contributes to the formation of early‐stage breast cancer myeloid‐derived suppressor cells. These cells suppress immune responses, facilitating cancer progression. PIAS1, which is overexpressed in human prostate cancer, downregulates p21 expression, thereby promoting cancer cell survival [223]. Conversely, PIAS3 overexpression has been shown to inhibit lung cancer cell proliferation and restore sensitivity to chemotherapeutic drugs [226]. Furthermore, increased PIAS3 expression induces apoptosis in cancer cells, highlighting its potential role in cancer suppression [227].

Several lncRNAs have been identified as enhancers of STAT3 expression. A correlation has been observed between tumor progression, poor patient prognosis, and a consistent reduction in the expression of these lncRNAs in cancerous tissues. Certain lncRNAs exhibit an inverse relationship with STAT3, suggesting their role as negative regulators of STAT3 signaling. Research has shown that specific miRNAs, including members of the miR‐548d‐3p and miR‐17 cluster families, as well as lncRNAs such as PTCSC3, MEG3, and lncRNA‐p21, can directly target STAT3 [27]. Given the diverse functions of STAT3, the development of novel small molecules capable of directly targeting STAT3 presents a promising approach for cancer therapy [8, 148]. Over the past three decades, multiple inhibitors targeting STAT3, either directly or indirectly, have been identified. Encouragingly, most of these inhibitors have demonstrated strong tumor‐suppressive effects in both preclinical and clinical studies. However, a specific STAT3 inhibitor has yet to receive approval for clinical use.

## 6 STAT3: Therapeutic Target

### 6.1 Small Molecule Inhibitor‐Based Therapies

Various small‐molecule inhibitors that directly target STAT3 have shown promising results in different in vitro models. However, no STAT3‐specific small‐molecule inhibitors employed in clinical trials due to limitations in conventional drug design methods and inefficiencies in screening processes [228]. The testing of thousands to millions of compounds remains impractical due to high failure rates, extensive time requirements, and significant development costs [229]. Therefore, identifying STAT3 inhibitors from naturally occurring compounds or repurposing existing drugs presents a more efficient and feasible approach. The pharmacokinetics and safety profiles of clinical drugs and natural products have already been established through absorption, distribution, metabolism, and elimination (ADME) studies [230]. Tables 1, 2, and 3 provide a comprehensive list of commonly used drugs that inhibit STAT3 in preclinical and clinical trials, offering potential avenues for accelerating the development of STAT3‐targeted therapies in clinical applications.

**TABLE 1:** STAT3 inhibitors currently in the pre‐clinical stage.

Names of inhibitors	Target	Mechanism of action	Preclinical outcomes	References
BP‐1–102	Direct inhibitor, SH2	Prevents nuclear translocation and STAT3 dimerization	Triggers apoptosis and suppresses development of tumor against glioma cells	[231]
Stattic	Direct inhibitor, SH2	Stops dimerization process	Promotes apoptosis and inhibits DNA‐binding capacity	[232, 233]
LLL12	Direct inhibitor, SH2	Inhibits phosphorylation at Tyr705	Enhances the suppression of tumor growth, inhibits cell migration, triggers apoptosis	[234]
SG‐1709	Direct inhibitor, DBD	Reduces STAT3 phosphorylation	Inhibits breast cancer growth	[235]
Periplogenin	Direct inhibitor, SH2	Suppresses Tyr705 phosphorylation of STAT3	Induces apoptosis and inhibits the growth of prostate cancer and esophageal squamous cell carcinoma	[236]
HP590, HJC0152	Direct inhibitor, DBD	Inhibits phosphorylation of STAT3	Prevents the growth of gastric cancer	[177, 237]
MS3‐6	Direct inhibitor, CCD	Decreases nuclear translocation and DNA binding	Interferes STAT3's interaction with the IL‐22 receptor and prevents STAT3‐dependent transcriptional activation	[23]
TG101209	Indirect inhibitor, JAK2	Inhibits signaling axis of JAK2/STAT3/c‐MYB	Prevents Burkitt lymphoma cell proliferation and trigger apoptosis	[238]
Cirsiliol	Indirect inhibitor, TYK2	Reduces STAT3 nucleus localization and dimer formation	Prevents the proliferation of esophageal squamous cell carcinoma	[239]
SC‐78	Indirect inhibitor, SHP1	Prevents STAT3 phosphorylation	Inhibits human colorectal cancer cells' stemness	[240]

*Abbreviations*: TYK2, tyrosine kinase 2; SHP1, Src homology region 2 (SH2) domain‐containing phosphatase 1.

**TABLE 2:** Novel small molecules as effective STAT3 inhibitors.

Name of compound	Mechanism of action	Indication	Phases	Clinical trial identifier	References
Napabucasin/BBI608	Phosphorylation inhibitor	NSCLC	3	NCT02826161	[241]
		Advanced malignancies	1/2	NCT01775423	[242]
		CRC	3	NCT01830621	[243]
		Metastatic colorectal cancer	3	NCT03522649	[195]
		Metastatic pancreatic ductal adenocarcinoma	3	NCT02993731	[244]
		Metastatic colorectal cancer	2	NCT03647839	[245]
Celecoxib	Phosphorylation inhibitor	CRC	3	NCT00087256	[246, 247]
C188‐9	Phosphorylation inhibitor	NSCLC, CRC, HNSCC, BC, HCC, melanoma, GAC, advanced cancer	1	NCT03195699	[248, 249]
OPB‐51602	Phosphorylation inhibitor	Advanced solid tumors	2	NCT01423903	[250]
		Nasopharyngeal carcinoma	1	NCT02058017	[251]
		Hematological malignancies	1	NCT01344876	[252]
OPB‐111077	Phosphorylation inhibitor	Solid tumors	1	NCT01711034	[253]
		Acute myeloid leukemia	1	NCT03197714	[254]
		Advanced HCC	1	NCT01942083	[255]
Disulfiram	Phosphorylation inhibitor	Metastatic pancreatic cancer and refractory solid tumors	1	NCT02671890	[250]
WP1220	Phosphorylation inhibitor	Cutaneous T‐cell lymphoma	1	NCT04702503	[256]
TTI‐101/C188‐9	Phosphorylation inhibitor	HCC, NSCLC, HNC, NSCLC, breast, gastric, colorectal melanoma	1	NCT03195699	[257]
Pyrimethamine	Phosphorylation inhibitor	Small lymphocytic lymphoma, CLL	1/2	NCT01066663	[258, 259]
AZD9150	Reduces the STAT3 protein's expression by attaching to its mRNA	Lymphoma	1/2	NCT01563302	[260]
Saracatinib	Targets SRC	Osteosarcoma	2	NCT00752206	[33]
Momelotinib	Targets JAK1/2	Non‐small‐cell lung cancer	1	NCT02258607	[261]

*Abbreviations*: NSCLC, non‐small cell lung cancer; CRC, colorectal cancer; HNSCC, head and neck squamous cell carcinomas; HCC, hepatocellular carcinoma; BC, breast cancer; GAC, gastric adenocarcinoma; HNC, head and neck cancer; CLL, chronic lymphocytic leukemia.

**TABLE 3:** Different drug delivery platforms supporting STAT3 inhibitors in cancer therapy.

Drugs	Name of material	Delivery routes	Cell/tissue specificity	References
Curcumin‐loaded liposomes‐STAT3 siRNA	Liposomes	Intratumorally administration	Skin cancer	[347]
DOX/CALP	Liposomes	Intratumorally administration	Ovarian cancer	[348]
Hyaluronic acid/TN‐CCLP	Liposomes	Intravenous administration	Breast cancer	[27]
LP‐R/C@AC NPs	Liposomes	—	Gastric cancer	[349]
Stattic	Liposomes	—	Melanoma cells	[350]
NP‐Stattic‐IL20RA	Liposomes	—	Breast cancer	[241]
HA/siSTAT3 PPLPTX	Polymer	Intravenous administration	Breast cancer	[351]
CSA/Gef‐NPs	Polymeric micelles	—	Lung cancer	[352]
SVMAV	Polymeric micelles	—	Melanoma	[353]
Gel‐NSC74859‐ICG	Polymer	Intravenous administration	Head and neck squamous cell carcinomas (HNSCCs)	[27]
Ritonavir derivative	Polymer	Intravenous administration	HNSCC	[354]
Cucurbitacin‐D; doxorubicin	Polymer	Intravenous administration	Breast cancer	[355]
siRNA‐SS‐PNIPAM	Polymeric micelles	—	Glioblastoma tumor	[356, 357]
Chol–DsiRNA Polyplexes	Polymeric micelles	Intravenous administration	Breast cancer	[358]
AuNP‐NUAP–STAT3d	Inorganic material	—	HNSCC cells	[27, 260]
LbL‐AuNP	Inorganic material	Intratumorally administration	Melanoma cells	[350]
AIRISE‐02 siRNA–CpG–mesoporous silica nanoparticle	Inorganic material	Intratumorally administration	Breast cancer	[359, 360]
CaP@LDL	Inorganic material	—	Hepatocellular carcinoma	[241]
CaP@HA	Inorganic material	—	Breast cancer	[361]
SPION–TMC–ChT–TAT–H NPs	Inorganic material	Intratumorally administration	Colorectal cancer	[362]
ZnAs@SiO2	Inorganic material	—	Hepatocellular carcinoma (HCC)	[363]
NPs‐αIL6R Ab–CD44	Biomimetic material	Intravenous administration	Breast cancer	[364]
CaP‐cored low‐density lipoprotein nanovehicle‐STAT3 decoy ODNs	Biomimetic material	Intravenous administration	HCC cells	[27]
Exo‐JSI124	Biomimetic material	Intranasal delivery	Glioblastoma tumor	[365]
EVs‐L‐PGDS	Exosome	—	Gastric cancer	[366]
Exo‐An2‐siRNA	Exosome	—	Glioblastoma tumor	[367]
CDNVs	Nanovesicles	—	Lung cancer	[368]

### 6.2 Targeting JAK

The activation of STAT3, primarily induced by various cytokines, is largely dependent on JAKs [262]. Multiple JAK inhibitors are currently under investigation and clinical evaluation for the treatment of cytokine release syndrome and chronic inflammatory diseases [263, 264]. Additionally, certain orally administered small‐molecule JAK inhibitors, which target ATP‐binding sites, are being explored for potential use in treating solid tumors [265, 266]. However, recent applications of JAK inhibitors have been predominantly directed toward hematological malignancies and inflammatory disorders [265, 267, 268].

Baricitinib and Tofacitinib are orally administered JAK inhibitors approved by the US FDA for the treatment of autoimmune diseases [21, 22]. Among the most extensively studied US FDA‐approved JAK inhibitors are Paclitaxel, Ruxolitinib, and Tofacitinib. Several related compounds are currently in early‐stage laboratory development before progressing to clinical evaluation. Tofacitinib effectively inhibits JAK1 and JAK3 but has a weaker inhibitory effect on JAK2 [269, 270]. Initially developed as an inhibitor of FMS‐like tyrosine kinase 3 (FLT3), lestaurtinib (CEP‐701) has also demonstrated JAK2 inhibition. It exhibits the potential to suppress cancer cell proliferation, limiting metastasis, and preventing colony formation in malignancies such as acute myeloid leukemia (AML), human neuroblastomas, and anaplastic thyroid carcinoma [271, 272]. Similarly, AZD1480 has shown antitumor activity in an HPV‐associated HNSCC animal model and inhibits IL‐6‐induced STAT3 phosphorylation [273, 274]. However, AZD1480 treatment in patients with solid tumors led to dose‐limiting toxicities in phase 1 clinical trials [27, 275].

WP1066 inhibits JAK2 phosphorylation and has demonstrated effectiveness in the treatment of AML, melanoma, and bladder cancer [196]. This inhibition enhances tumor sensitivity to chemotherapy across various cancer models. WP1066 specifically targets the STAT3/miR‐21 axis, increasing the susceptibility of oral squamous cell carcinoma cells to cisplatin [196]. The combination of WP1066 with a dexamethasone derivative (DX10) has shown potential in melanoma treatment [276, 277]. In a xenograft tumor model using Tca8113/DDP cells, the combined administration of WP1066 and cisplatin significantly reduced tumor growth [27]. Additionally, studies have reported that AG490, a JAK2 inhibitor, suppresses angiogenesis and reduces MDSCs within the HNSCC TME by inhibiting the JAK2/STAT3 pathway [27, 278].

Rufolitinib (INC424), a JAK1/2 inhibitor, has been approved by the US FDA for the treatment of inflammatory diseases [279]. As a repurposed drug, it has demonstrated good tolerance and an overall hematologic response rate of about 32% in patients with abnormal chronic myeloid leukemia and chronic neutrophilic leukemia [280]. Similarly, pacritinib (SB1518) is an orally administered inhibitor that competes with ATP to block both JAK2 and FLT3, thereby suppressing the growth of various cancer types. Currently, pacritinib is in the first phase of clinical trials for potential lymphoma treatment [281].

Several natural compounds have been identified as inhibitors of the JAK/STAT3 pathway. Arctiin has been shown to deactivate JAK and Src while inhibiting excessive pSTAT3 expression [18]. Additionally, 8α‐tigloyloxyhirsutinolide‐13‐O‐acetate, a bioactive compound derived from Vernonia cinerea, inhibits the JAK/STAT3 pathway and exhibits antitumor effects in an HNSCC mouse model [27]. The indirubin derivative E738 competes with ATP to inhibit JAKs and SFKs, effectively reducing STAT3 expression in malignant cells [282].

### 6.3 Targeting IL‐6 and IL‐6R

The initial step in STAT3 activation involves the interaction between cytokines and their respective receptors. Three primary strategies are employed to inhibit IL‐6‐mediated signaling: the use of fusion proteins, such as sgp130, to target the IL‐6‐soluble IL‐6R complex; the direct neutralization of IL‐6 with antibodies like siltuximab; and the blockade of the IL‐6R with antibodies such as tocilizumab [283, 284]. Targeting the IL‐6–sIL‐6R complex with sgp130 fusion proteins selectively inhibits trans‐signaling, while direct inhibition of IL‐6 or IL‐6R effectively suppresses both classical and trans‐signaling pathways [201].

Siltuximab (CNTO‐328), a chimeric mouse‐human monoclonal antibody, selectively binds to IL‐6, preventing its interaction with IL‐6R and thereby disrupting the IL‐6/STAT3 signaling pathway. This inhibition has demonstrated efficacy in reducing tumor growth and invasion in non‐small cell lung cancer (NSCLC) and cholangiocarcinoma [285, 286]. Following multiple clinical trials, siltuximab received US FDA approval in 2014 for the treatment of multicentric Castleman disease [287]. Additionally, it has shown antitumor activity in ovarian [288], prostate [289], and lung cancer [290]. In a phase I–II clinical trial, siltuximab treatment led to a reduction in active STAT3 and MAPK levels in prostate cancer patients [291]. Similarly, in a separate phase I–II trial, over 50% of individuals with metastatic renal carcinoma exhibited disease stabilization following siltuximab administration [292]. However, no significant clinical efficacy was observed in advanced‐stage cancers, including head and neck, lung (NSCLC), colorectal, pancreatic, or ovarian cancers [293]. While preclinical studies have shown promising outcomes, clinical trials have yielded limited success in solid tumors. These findings suggest that targeting IL‐6 alone may not be sufficient for solid tumor treatment, highlighting the need for combination therapies and the identification of reliable predictive biomarkers.

Clazakizumab, olokizumab, MEDI5117, and sirukumab are anti‐IL‐6 antibodies currently under evaluation for cancer treatment; however, these agents remain in the early stages of development [82, 294]. These antibodies inhibit IL‐6‐mediated signaling cascades involving JAK and STAT3 across various cancer types. Olokizumab specifically interferes with the interaction between the gp130 signal‐transducing component and IL‐6–IL‐6R and IL‐6 dimers, thereby preventing the formation of hexamers [295].

Tocilizumab binds to IL‐6R and inhibits both trans‐signaling and classical signaling pathways [201]. The US FDA has approved its use for managing cytokine‐release syndrome in patients with B‐cell acute lymphoblastic leukemia undergoing chimeric antigen receptor T‐cell therapy, as well as for the treatment of RA and systemic juvenile idiopathic arthritis [296]. Tocilizumab has also demonstrated efficacy in ovarian [288], pancreatic [297], and colorectal cancers associated with colitis [298]. In a phase I clinical study, the combination of tocilizumab with carboplatin and/or doxorubicin (Dox) in ovarian cancer patients showed promising results [299, 300]. Additionally, early‐phase trials are being conducted to evaluate the efficacy and safety of tocilizumab in pancreatic cancer, breast cancer, and B‐cell chronic lymphocytic leukemia [301, 302].

Selective inhibition of the trans‐signaling pathway may be beneficial for tumor patients with limited or no IL‐6R expression. Proteins containing the sgp130 sequence selectively inhibit trans‐signaling by binding to and blocking the IL‐6R and IL‐6 complex [201]. Preclinical studies have demonstrated that the sgp130‐Fc fusion protein suppresses the growth and metastasis of pancreatic cancer, colitis‐associated premalignant colorectal cancer, and KRAS‐driven NSCLC [27]. IBD and RA patients are currently being treated with olamkicept, an sgp130‐Fc fusion protein, in phase I–Ib clinical trials. However, targeted inhibition of IL‐6 signaling may reduce STAT1 activity, which possesses tumor‐suppressive properties, presenting a potential challenge for cancer treatment [294].

Bazedoxifene, a third‐generation selective estrogen receptor modulator, inhibits GP130, IL‐6, and IL‐6R complexes, thereby preventing subsequent STAT3 activation [303]. It has shown promising effects against pancreatic cancer by reducing cancer cell proliferation and migration. Additionally, its therapeutic efficacy is enhanced when combined with other chemotherapeutic agents [304, 305].

### 6.4 SH2 Domain Inhibitors

The SH2 domain of STAT3 enables its binding to tyrosine‐phosphorylated residues on cell surface receptors, which is crucial for its activation. Additionally, the SH2 domain is essential for the formation of STAT3 dimers, where one SH2 domain binds to a phosphorylated tyrosine residue on another STAT3 molecule. Inhibiting the SH2 domain effectively blocks STAT3 activation and phosphorylation. Targeting the SH2 domain allows direct STAT3 inhibition through two primary mechanisms: first, by preventing Tyr705 phosphorylation on STAT3 at the cell membrane through RTKs or nonreceptor kinases, and second, by interfering with the formation of functional STAT3 dimers. Two classes of inhibitors, peptides, and small molecules, target the SH2 domain and have demonstrated the ability to limit tumor cell proliferation [24, 195]. Peptidomimetics mimic a specific protein sequence, pTyr–Xaa–Yaa–Gln, and bind to the STAT3 SH2 domain, competing with its natural binding partners to prevent dimerization [306]. A notable example is the phosphopeptide inhibitor derived from the PY*LKTK sequence, where Y* represents phosphorylated tyrosine. This small molecule directly binds to STAT3, interfering with dimerization and inhibiting its activity. Other compounds, including C‐pTyr–Leu–Pro–Gln–Thr–Val–NH2, BP–PM6, BP–PM7, and PMM‐172, have been shown to suppress STAT3 by reducing its persistent phosphorylation in HNSCC and breast cancer through SH2 domain inhibition [27].

Phosphorylation of the SH2 domain at Tyr‐705 is essential for STAT3 dimerization and DNA binding. Therefore, inhibitors targeting the STAT3 SH2 domain also disrupt its interaction with DNA [16]. An oxazole‐based peptidomimetic, S3I‐M2001, has been reported to specifically inhibit STAT3 dimerization, thereby suppressing transcription, transformation, survival, and migration in both mouse and human cells [196, 307]. Another peptidomimetic, S3I‐1757, derived from benzoic acid, directly interacts with the Tyr‐705 binding region within the STAT3 SH2 domain, limiting hyperactivation and reducing malignant transformation. By preventing STAT3 dimerization and DNA binding, this inhibition leads to apoptosis and reduced cell proliferation through the suppression of key STAT3 target genes, including cyclin D1, Bcl‐xL, MMP‐9, and survivin [308]. Similarly, S3I‐201, a salicylic acid‐derived compound, and its analogs inhibit STAT3 DNA binding by interacting with the SH2 domain. This inhibition induces apoptosis in cancer cells by downregulating proteins essential for cell survival and proliferation, such as survivin, Bcl‐xL, and cyclin D [23, 24]. Additionally, S3I‐201 has been shown to inhibit STAT3 activation in a mouse model of anal squamous cancer negative for HPV, suppressing cancer cell growth and reducing their ability to evade immune responses [27].

Phosphopeptides exhibit limited cell permeability, prompting the investigation of a new class of small molecules capable of inhibiting the STAT3 SH2 domain. One such compound, STA‐21, a naturally occurring deoxytetrangomycin, selectively binds to the SH2 domain, preventing STAT3 dimerization and nuclear translocation, thereby significantly reducing the proliferation and progression of breast cancer cells [260]. Another small molecule, STATTIC, specifically disrupts STAT3 dimerization and DNA binding while also inhibiting enzymes responsible for STAT3 activation, leading to apoptosis in breast cancer cells [309]. Similarly, BP‐1–102 targets the STAT3 SH2 domain, effectively suppressing cell survival, growth, migration, and invasion in lung and breast cancer [310].

Several nonpeptide small molecules have also been identified as STAT3 inhibitors [196, 311]. OPB‐31121 has demonstrated strong antitumor activity, particularly in multiple liver cancer models [312]. Likewise, OPB‐51602 interacts with the SH2 domain, interfering with intradomain interactions and causing STAT3 aggregation. This disruption affects mitochondrial function, ultimately inducing cancer cell death [24].

Curcumin, a natural compound‐derived inhibitor, targets the SH2 domain of STAT3. When modified with proline, curcumin effectively inhibits STAT3 dimerization [23]. Similarly, cryptotanshinone, a naturally occurring compound, interacts with the SH2 domain to suppress STAT3 phosphorylation and prevent dimer formation, thereby reducing the expression of cell survival genes such as Bcl‐xL, survivin, and cyclin D1 [313]. Additionally, several other natural compounds, including cucurbitacin E [191], alantolactone [314], piperlongumine [315], and silibinin [316], have demonstrated the ability to inhibit STAT3 by binding to the SH2 domain, highlighting their potential as therapeutic agents targeting STAT3 signaling.

Celecoxib, a COX‐2 inhibitor, also binds to the SH2 domain of STAT3. By competitively inhibiting native peptide binding, celecoxib reduces tyrosine phosphorylation, leading to decreased cell motility and viability [317, 318]. A separate class of STAT3 dimerization antagonists, derived from salicylic acid, exhibits enhanced membrane permeability, offering an advantage over peptidomimetics. These compounds effectively disrupt STAT3–phosphopeptide interactions, thereby preventing STAT3 dimerization. Additionally, they induce apoptosis and inhibit intracellular STAT3 phosphorylation [319]. The SH2 peptide inhibitor (SPI), a 28‐amino acid peptide, blocks the interaction between the STAT3 SH2 domain and phosphorylated tyrosine on IL‐6R. SPI suppresses STAT3 activation and promotes apoptosis, demonstrating potential as a therapeutic agent for STAT3‐driven malignancies [320]. Furthermore, ODZ10117, another SH2 domain inhibitor, has been shown to prevent tyrosine phosphorylation and STAT3 dimer formation, ultimately reducing tumor progression [321, 322].

### 6.5 STAT3 DBD Targeting

**FIGURE 5:**
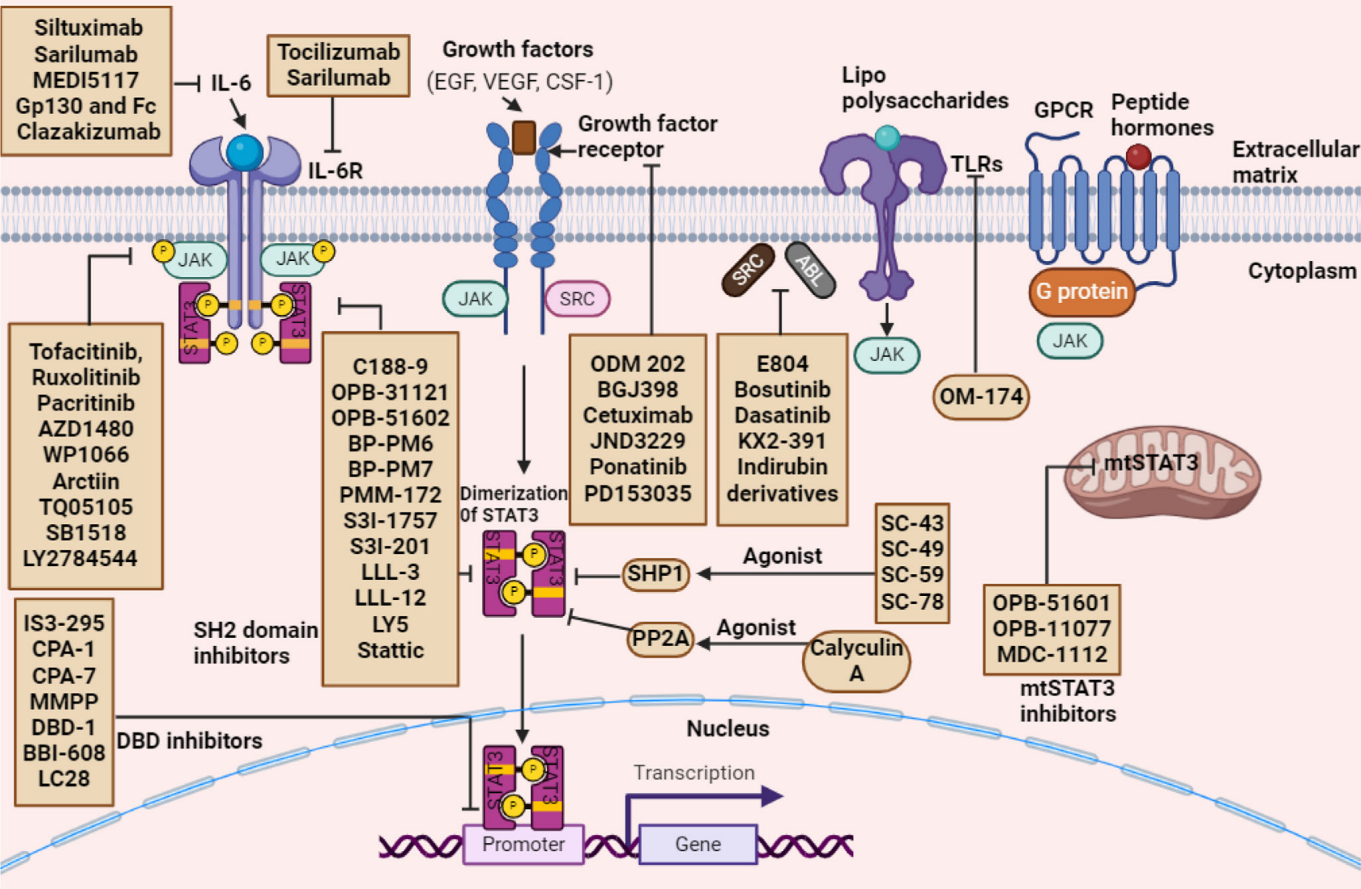
The STAT3 signaling pathway can be targeted using various therapeutic agents. Monoclonal antibodies that inhibit IL‐6 include MEDI5117, clazakizumab, olokizumab, siltuximab, and sarilumab. Additionally, monoclonal antibodies such as sarilumab and tocilizumab block both classical and trans‐signaling pathways of IL‐6R. Olamkicept, a fusion protein of gp130 and Fc, selectively inhibits IL‐6 trans‐signaling while modulating classical signaling. Tyrosine kinase inhibitors, including tofacitinib, ruxolitinib, pacritinib, and AZD1480, are small molecules that target JAKs, preventing STAT3 phosphorylation. Other inhibitors, such as those targeting the Src homology 2 (SH2) domain—C188‐9, OPB‐51602, and OPB‐31121—disrupt STAT3 dimerization. AZD9150, an antisense oligonucleotide, binds to and degrades STAT3 mRNA, thereby reducing its expression. Additionally, cyclic STAT3 decoy, derived from the FOS target gene promoter, competitively inhibits STAT3 binding to genomic response elements in target gene promoter regions, thereby suppressing STAT3‐mediated transcription. Created with Biorender.com. SHP1, Src homology region 2 (SH2) domain‐containing phosphatase 1; PP2A, protein phosphatase 2A.

Targeted gene promoter sites interact with the DBD of STAT3, which exhibits relatively high specificity. STAT3 plays a crucial role in cell proliferation, migration, and invasion by binding to DNA within the cell nucleus. Its activity can be reduced by inhibitors that target the STAT3 DBD, thereby preventing its interaction with DNA [18]. C468 is the first identified small‐molecule inhibitor of the STAT3 DBD, binding to cysteine (C468) on glutathione sulfhydryl within the DBD region. This interaction prevents activated STAT3 from accumulating in tumor cell nuclei, leading to a significant reduction in tumor growth [323]. The platinum (IV) compound IS3‐295 disrupts the DNA‐binding ability of STAT3 through a noncompetitive mechanism [308]. Additionally, apoptosis in human cancer cells is induced by various platinum (IV) compounds, including CPA‐1, CPA‐7, and platinum (IV) tetrachloride, which inhibit STAT3 DNA binding [16]. A study synthesized (E)‐2‐methoxy‐4‐(3‐(4‐methoxyphenyl) prop‐1‐en‐1‐yl) phenol (MMPP), a novel small molecule that effectively inhibits cancer progression by targeting the STAT3 DBD. MMPP exhibits selective binding, reducing the likelihood of nonspecific interactions and associated side effects [324]. Using a STAT3 decoy to target activated STAT3 has been shown to inhibit cancer cell growth, induce apoptosis, and suppress STAT3‐mediated gene expression in head and HNSCC cells [325]. One study reported about a 7.4‐fold increase in programmed cell death in an HNSCC xenograft model treated with a combination of cisplatin and a STAT3 decoy. Additionally, a series of G‐quartet oligodeoxynucleotides (GQ‐ODNs) have been synthesized to inhibit the DNA‐binding activity of STAT3. Treatment of xenograft HNSCC tumors with GQ‐ODN in combination with paclitaxel for 21 days resulted in a 35% reduction in average tumor size [27, 326]. The STAT3 DBD can be selectively targeted using DBD‐1, a small peptide aptamer. In a murine model, the interaction between the DBD of STAT3 and DBD‐1 was weak. However, in murine carcinoma B16 cells, DBD‐1 significantly induced apoptosis [327]. Figure 5 depicts the targeting of STAT3 by several substances.

### 6.6 Challenges and Limitations of Different STAT3 Inhibitors

The therapeutic targeting of STAT3 presents several challenges and limitations across different classes of inhibitors. Small‐molecule inhibitors often exhibit poor selectivity and specificity, leading to systemic toxicity, off‐target effects, and the development of resistance through the activation of alternative signaling pathways. Additionally, these inhibitors face issues related to cell permeability and bioavailability, which limit their overall therapeutic potential [196, 308]. Similarly, JAK inhibitors, which target upstream kinases, exert broad effects on multiple STAT proteins. Their inhibition of the JAK–STAT pathway can lead to immune suppression and hematological toxicity, along with the activation of compensatory pathways that may reduce their effectiveness [328, 329].

IL‐6 and IL‐6R inhibitors also encounter significant challenges due to the pleiotropic functions of IL‐6 in metabolism, inflammation, and immune regulation [68, 330]. These inhibitors may cause systemic adverse effects and only partially suppress STAT3, as other pathways, such as those activated by growth factors, can also contribute to STAT3 activation [82, 331]. Similarly, SH2 domain inhibitors may only partially block STAT3 and often struggle with achieving high affinity and selectivity due to structural similarities with other STAT proteins. Mutations in the SH2 domain and suboptimal pharmacokinetics further compromise their efficacy [195, 230]. Inhibitors targeting the DBD of STAT3 face additional challenges, including limited accessibility due to the nuclear localization of the target and the complexity of designing inhibitors that can selectively bind to the large, flat surface of the DBD. Furthermore, inhibiting the DBD may disrupt the transcription of genes essential for normal cellular function, increasing the risk of off‐target effects [332, 333]. To address limitations related to specificity, efficacy, and safety, novel approaches such as combination therapies and advanced DDSs, including nanoparticle (NP)‐based platforms, may enhance the effectiveness of STAT3‐targeted treatments.

## 7 Combination Strategies With STAT3 Inhibitors

The combination of STAT3 inhibitors has the potential to enhance tumor suppression and counteract resistance mechanisms through a synergistic approach. Modulating the TME using this strategy may improve the efficacy of immune checkpoint inhibitors and facilitate the elimination of resistant CSCs. Additionally, combination therapies could allow for reduced drug dosages, thereby minimizing systemic toxicity and adverse effects [190, 334].

The combination of immune checkpoint inhibitors, such as anti‐PD‐1 or anti‐PD‐L1 antibodies, with STAT3 inhibitors represents a promising strategy for cancer therapy. Preclinical studies in lung cancer and melanoma models have shown that STAT3 inhibition enhances antitumor immunity by modulating the TME and reducing PD‐L1 expression [335, 336]. Chemotherapeutic agents, such as gemcitabine and Dox, also demonstrate synergistic effects when combined with STAT3 inhibitors. For instance, apabucasin enhances the efficacy of gemcitabine in pancreatic cancer, while FLLL32, a curcumin derivative, increases apoptosis in triple‐negative breast cancer (TNBC) when used in combination with Dox [235]. STAT3 inhibitors have also been evaluated in combination with targeted therapies, such as EGFR inhibitors (e.g., gefitinib) or JAK inhibitors (e.g., ruxolitinib), for malignancies driven by these pathways [308]. In glioblastoma models, STAT3 inhibition has been found to enhance the effects of radiation therapy by disrupting DNA repair and survival pathways. Additionally, the combination of cyclophosphamide with natural compounds such as arctigenin has exhibited enhanced anticancer activity, particularly against TNBC [315]. Similarly, combining STAT3 inhibitors with epigenetic modulators, such as HDAC inhibitors or DNA methyltransferase inhibitors, has demonstrated efficacy against both solid tumors and hematological malignancies [337]. The STAT3 SH2 domain inhibitor YHO‐1701 has shown significant synergy with alectinib in NCI‐H2228 xenografts, reducing body weight loss while avoiding systemic toxicity. Furthermore, YHO‐1701 has improved the anticancer effects of sorafenib in an SAS xenograft model that secretes IL‐6 [307]. In medulloblastoma xenografts, the combination of cisplatin and LLL12B has effectively suppressed tumor growth in D283 and D425 models [338].

While these strategies highlight the versatility of STAT3 inhibitors, several challenges remain, including overlapping toxicities, potential resistance mechanisms, and tumor heterogeneity, which necessitate personalized treatment approaches. Clinical trials are essential for optimizing these combination therapies, with a focus on establishing their efficacy and safety in diverse patient populations.

## 8 Decreasing STAT3 Expression

Antisense oligonucleotides (ASOs) inhibit STAT3 mRNA translation by binding to complementary single‐stranded RNA sequences [260]. A specific type of ASO, modified with 2‐O‐methoxyethyl, has been shown to reduce circulating VEGF levels, suppress neovascularization, and inhibit cancer cell proliferation and metastasis [339]. Oweida et al. [340] reported that the combination of STAT3 ASOs with radiotherapy enhanced antitumor effects and reduced radiation resistance. In LY2 and MOC2 tumor‐bearing mice, the average tumor volumes following combined radiation and STAT3 ASO treatment were 53.0 ±5.6 and 254.8 ±81.6 mm^3^, respectively. In contrast, tumor volumes in mice treated only with STAT3 ASOs were 277.4 ±53.8 and 1042.9 ±326.8 mm^3^, respectively [340]. Posttranscriptional inhibition of STAT3 is commonly achieved using RNA interference, such as siRNA. The STAT3 inhibitor STX‐0119 has exhibited cytotoxic effects against various pancreatic cancer cell types, particularly those with low PD‐L1 expression, highlighting its potential therapeutic relevance [341].

AZD9150 is a specialized ASO designed to bind to the 3′‐untranslated region of the STAT3 gene. In lung cancer and lymphoma models, AZD9150 has been shown to effectively reduce STAT3 activity and its downstream targets by decreasing STAT3 mRNA levels [25]. When combined with cisplatin, AZD9150 significantly enhanced tumor sensitivity and improved survival rates compared with monotherapy with either drug alone [342]. In addition to ASOs, miR‐124‐3p has been identified as a regulatory molecule that interacts with the 3′ untranslated region of STAT3, leading to its transcriptional downregulation. This mechanism induces apoptosis in NPC cells and inhibits their proliferation, migration, and invasion [343]. A distinct approach to inhibiting STAT3 activity involves the use of ODNs, which capture active STAT3 dimers in the cytoplasm and prevent their interaction with importin, thereby blocking nuclear translocation [260, 344]. Another effective strategy for suppressing STAT3 involves siRNA, which degrades STAT3 mRNA. By targeting STAT3 mRNA, siRNA reduces the levels of antiapoptotic proteins such as Bcl‐xL and Bcl‐2, ultimately triggering cell death [260].

## 9 Targeted Delivery of STAT3 Inhibitors

The clinical application of STAT3 inhibitors presents several challenges, including low oral bioavailability, nonspecific targeting, and potential toxicity to healthy cells [308, 345]. To address these limitations, research efforts have focused on developing advanced DDSs that encapsulate STAT3 inhibitors using nanomaterials such as polymers, liposomes, inorganic materials, and biomimetic carriers. Nanomaterial‐based drug delivery approaches offer several advantages over conventional STAT3 inhibitors. These systems exhibit high biocompatibility and improved tumor‐targeting capabilities, thereby minimizing damage to healthy tissues. Additionally, their high drug‐loading capacity allows for the simultaneous delivery of multiple therapeutic agents. Furthermore, nanomaterials protect encapsulated drugs from rapid clearance in the bloodstream, thereby enhancing their stability and prolonging therapeutic efficacy [241, 346].

Liposomes are nanoscale, spherical vesicles composed of lipid bilayers, typically ranging from 50 to 500 nm in diameter. They form when natural or synthetic lipids are dispersed in water, creating one or more lipid layers surrounding an aqueous core [135, 369]. Due to their structural similarity to cell membranes, high drug‐loading capacity, and biocompatibility, liposomes effectively encapsulate hydrophobic drugs within their lipid bilayers [370, 371]. A calcium phosphate‐core low‐density lipoprotein nanocarrier was utilized by Shi et al. [372] to develop a Trojan horse strategy for delivering STAT3 decoy ODNs, effectively overcoming resistance to TNF‐related apoptosis‐inducing ligand. Additionally, PEGylated liposomal FLLL32, a specialized STAT3 inhibitor targeting the SH2 domain, has demonstrated enhanced antitumor activity while reducing systemic toxicity [373, 374]. Recent advancements in liposomal drug delivery have led to the development of a dual‐drug‐loaded liposome for gastric cancer treatment, where a hybrid membrane (R/C) (LP‐R/C@AC NPs) is coated with cinobufagin and apatinib. By inhibiting the VEGFR2/STAT3 pathway, LP‐R/C@AC NPs effectively suppress cancer cell proliferation and reverse immune suppression by downregulating MMP‐9 and PD‐L1 expression. This approach has shown a two‐fold increase in tumor targeting and immune system modulation [241]. Exosomes, a type of liposome‐like vesicle containing antigens, mRNAs, and miRNAs, have emerged as promising drug delivery vehicles capable of overcoming therapeutic barriers in cancer treatment. Intranasal administration of exosome‐packaged STAT3 inhibitors has been explored as a noninvasive therapeutic strategy. Studies have shown that microglial cells can absorb these inhibitors, leading to a 44.5‐day increase in survival in GL26 tumor‐bearing animals compared with control groups. Exosomes secreted by brain endothelial cells can penetrate the blood–brain barrier, allowing targeted drug delivery for lung cancer‐associated brain metastases. This capability presents a promising approach for effective cancer therapy [365, 375].

To achieve sustained drug release, Zheng et al. [376] utilized polylactic‐co‐glycolic acid (PLGA) nanomaterials for the co‐delivery of the STAT3 inhibitor nifurate and Dox. Another study employed HPMA‐based copolymers containing Dox and the STAT3 inhibitor cucurbitacin D for gradual drug release, demonstrating effectiveness in breast cancer treatment [355]. Additionally, DDSs such as PLGA‐JSI‐124 conjugation, PEO‐b‐P(CL–JSI‐124) conjugates, and PEO‐b‐PBCL micelles have exhibited improved therapeutic efficacy in cancer models [27]. A novel drug delivery approach has been proposed for direct pulmonary administration using fine droplets (FM@PFC/siRNA) composed of perfluorocarbon (PFC). This system delivers anti‐STAT3 siRNA alongside a C‐X‐C motif chemokine receptor 4 (FM) antagonist (FM). The FM@PFC/siRNA nanoemulsions effectively inhibit STAT3 and CXCR4 signaling pathways, leading to increased apoptosis and reduced cancer cell invasion. Furthermore, in a lung metastatic tumor model, this method successfully counteracted the immunosuppressive TME, highlighting its potential in targeted lung cancer therapy [377, 378].

Over the past two decades, inorganic NPs, including magnetic, mesoporous silica, and gold/silver NPs, have been extensively studied as potential cancer therapeutics. These NPs offer several advantages, including ease of synthesis, high biocompatibility, and the ability to functionalize their surfaces for targeted drug delivery [379, 380, 381]. In one study, imatinib mesylate and anti‐STAT3 siRNA were encapsulated within layer‐by‐layer assembled gold NPs (LbL‐AuNPs) for melanoma treatment. These positively charged NPs were constructed through the sequential adsorption of sodium alginate, chitosan, and natural polyelectrolytes. The positive charge facilitated the use of iontophoresis therapy, which enhances skin penetration for localized melanoma treatment. When applied topically by iontophoresis, these dual‐drug‐loaded LbL‐AuNPs significantly suppressed tumor growth and reduced STAT3 expression in melanoma mouse models [350, 382]. Additionally, ZnAs@SiO2 NPs have been shown to downregulate stemness markers (Oct‐4, Sox‐2, and CD133) and EMT markers (slug, vimentin, and E‐cadherin). The inhibition of the STAT3 signaling pathway by these NPs also contributed to a reduction in tumor spheroid formation in a three‐dimensional model [363].

The combination of STAT3 inhibitors with other therapeutic agents enhances both the safety and efficacy of cancer treatment while improving the therapeutic potential of STAT3 blockade through advanced drug delivery modalities. Immunotherapy targeting immune checkpoint proteins has emerged as a promising strategy for cancer management. However, NSCLC has demonstrated resistance to tremelimumab (anti‐CTLA4 antibody) and durvalumab (anti‐PD‐L1 antibody) due to STK11 gene alterations, which impair immune system efficacy. STAT3 ASOs can counteract this inhibitory effect, significantly improving the antitumor response when administered in combination with immune checkpoint inhibitors [383]. Additionally, DDSs integrated with anti‐STAT3 inhibitors have demonstrated superior outcomes in cancer treatment compared with the use of inhibitors alone, highlighting the potential of NP‐based approaches to enhance drug targeting, reduce systemic toxicity, and improve overall therapeutic efficacy [308].

The development of DDSs for STAT3 represents an advanced strategy aimed at addressing the limitations of conventional therapies. These systems enhance therapeutic efficacy by reducing systemic toxicity, improving bioavailability, and optimizing targeted drug delivery. Various DDSs, including NPs, liposomes, micelles, dendrimers, and hydrogels, have been explored to increase the accumulation of STAT3 inhibitors at tumor sites. Recent advancements in stimuli‐responsive DDSs, such as pH‐, temperature‐, or enzyme‐sensitive systems, have facilitated the controlled release of STAT3 inhibitors within the TME. These approaches enhance local drug concentrations while minimizing damage to healthy tissues [384, 385, 386]. Additionally, DDSs improve drug transport and targeting, maximizing tumor site concentration while reducing systemic exposure and associated toxicity. Furthermore, these systems can effectively penetrate physiological barriers, including intracellular compartments and the TME. DDSs can also be engineered to respond to specific signals within the TME, further enhancing the precision of drug delivery [387, 388].

Despite promising results, the development of DDSs for STAT3 inhibitors still presents several challenges. One of the primary difficulties is designing systems that can selectively target and penetrate tumor tissues while minimizing off‐target effects on healthy cells. Although functionalization enhances targeting, tumor heterogeneity and the dynamic nature of the TME complicate the consistent and specific delivery of drugs to STAT3‐overexpressing cells. Additionally, the scalability and stability of these delivery platforms for clinical applications require further investigation. The potential toxicity and immunogenicity of certain DDS components, along with the capacity of cancer cells to develop resistance mechanisms, may reduce long‐term therapeutic efficacy [389, 390]. While DDSs offer significant advantages for STAT3‐targeted cancer therapy, further research is necessary to address these limitations and ensure clinical feasibility.

## 10 Conclusion and Future Prospective

STAT3 is an essential protein present in all tissues, playing a crucial role in cellular processes and influencing the growth and function of multiple physiological systems. Upon activation by growth factors or cytokines, STAT3 enhances the expression of genes that regulate the cell cycle and promote cell proliferation. Dysregulated STAT3 activity, whether through hyperactivation or inactivation, has been implicated in various human diseases, including atherosclerosis, bone‐related disorders, neurological conditions, autoimmune diseases, and cancer. Despite its potential as a therapeutic target in cancer, existing STAT3 inhibitors exhibit limited potency and provide only modest clinical benefits. Therefore, exploring novel strategies for targeting STAT3 in cancer therapy remains a priority. Since STAT3 signaling is regulated by molecules such as miRNAs, TLRs, and GPCRs, small‐molecule inhibitors that selectively target these pathways may effectively suppress STAT3 activity, thereby reducing tumor progression and inflammation. Furthermore, targeting STAT3 epigenetic modifications presents an emerging and promising approach for cancer treatment.

Despite advancements in STAT3‐targeted therapies, direct inhibition of STAT3 remains challenging due to its ubiquitous expression in the body. To date, only a limited number of STAT3 inhibitors have been approved by the USFDA for cancer treatment, and several concerns persist. One major issue is the need for specificity, as these inhibitors must selectively target STAT3 without affecting structurally similar proteins, such as STAT1. Additionally, adverse effects, including hematological toxicity, remain a significant challenge. Further research should focus on improving the specificity and safety profile of STAT3 inhibitors to minimize off‐target effects. Current inhibitors primarily target STAT3 dimerization, but assessing their interactions with other proteins remains difficult. Therefore, beyond the SH2 domain, additional regions of STAT3 should be investigated as potential therapeutic targets. Moreover, reducing the dosage of STAT3 inhibitors and combining them with immunotherapy, chemotherapy, or personalized treatment approaches may enhance therapeutic efficacy while mitigating adverse effects. Additionally, the development of advanced DDSs is essential to overcome the limitations of conventional therapies. DDSs offer the potential to enhance the therapeutic profile of STAT3 inhibitors by improving bioavailability, reducing systemic toxicity, and ensuring targeted drug delivery. The integration of STAT3 inhibitors with other anticancer agents and optimizing DDSs could significantly improve treatment outcomes in cancer therapy [27].

Advancements in understanding the STAT3 signaling pathway and the development of STAT3 inhibitors have underscored their potential in cancer therapy. Future research may focus on integrating STAT3‐targeted therapies with conventional treatments in a multimodal approach to enhance therapeutic outcomes. Although various strategies have been explored to identify small molecules capable of inhibiting STAT3 signaling, further investigations are required to optimize their efficacy and clinical applicability.

## Author Contributions

M. A. S., I. A., A. A., and A. H. conceived, designed, and performed the literature survey, and drafted the article's first draft. F. A., M. H. A., A. K., and S. T. provided the critical feedback, finalized the content, and made the final draft. All authors have accepted responsibility for the entire content of this manuscript and approved its submission.

## Ethics Statement

The authors have nothing to report.

## Conflicts of Interest

The authors declare no conflicts of interest.

## Data Availability

The authors have nothing to report.

## References

[1] S. Chan , Z. Liu , Y. Chen , et al., “The JAK‐STAT Signaling‐Related Signature Serves as a Prognostic and Predictive Biomarker for Renal Cell Carcinoma Immunotherapy,” Gene 927 (2024): 148719. 38917875 10.1016/j.gene.2024.148719

[2] X. H. Xu , J. X. Zhang , H. X. Liu , Z. Zhao , and J. Y. Jiang , “Intervention of inflammation associated With ankylosing spondylitis by triptolide promotes histone H3 Iys‐27 trimethylation,” Immunopharmacology and Immunotoxicology 46, no. 6 (2024): 785–792. 39307916 10.1080/08923973.2024.2402911

[3] Q. Hu , Q. Bian , D. Rong , et al., “JAK/STAT pathway: Extracellular signals, diseases, immunity, and therapeutic regimens,” Frontiers in Bioengineering and Biotechnology 11 (2023): 1110765. 36911202 10.3389/fbioe.2023.1110765 PMC9995824

[4] S. Wei , J. Li , M. Tang , et al., “STAT3 and p63 in the Regulation of Cancer Stemness,” Frontiers in Genetics 13 (2022): 909251. 36061200 10.3389/fgene.2022.909251 PMC9428145

[5] W. Wang , M. C. Lopez McDonald , C. Kim , et al., “The complementary roles of STAT3 and STAT1 in cancer biology: Insights Into tumor pathogenesis and therapeutic strategies,” Frontiers in Immunology 14 (2023): 1265818. 38022653 10.3389/fimmu.2023.1265818 PMC10663227

[6] M. Hashemi , S. Abbaszadeh , M. Rashidi , et al., “STAT3 as a newly emerging target in colorectal cancer therapy: Tumorigenesis, therapy response, and pharmacological/nanoplatform strategies,” Environmental Research 233 (2023): 116458. 37348629 10.1016/j.envres.2023.116458

[7] N. Jill , S. Bhootra , S. Kannanthodi , et al., “Interplay Between signal transducers and activators of transcription (STAT) proteins and cancer: Involvement, therapeutic and prognostic perspective,” Clinical and Experimental Medicine 23, no. 8 (2023): 4323–4339. 37775649 10.1007/s10238-023-01198-8

[8] C. D. Mohan , S. Rangappa , H. D. Preetham , et al., “Targeting STAT3 signaling pathway in cancer by agents derived From Mother Nature,” Seminars in Cancer Biology 80 (2022): 157–182. 32325172 10.1016/j.semcancer.2020.03.016

[9] M. Tolomeo and A. Cascio , “The Multifaced Role of STAT3 in Cancer and Its Implication for Anticancer Therapy,” International Journal of Molecular Sciences 22, no. 2 (2021): 603. 33435349 10.3390/ijms22020603 PMC7826746

[10] P. Faida , M. K. I. Attiogbe , U. Majeed , J. Zhao , L. Qu , and D. Fan , “Lung cancer treatment potential and limits associated With the STAT family of transcription factors,” Cell Signalling 109 (2023): 110797. 37423343 10.1016/j.cellsig.2023.110797

[11] J. Li , Z. Yin , B. Huang , K. Xu , and J. Su , “Stat3 Signaling Pathway: A Future Therapeutic Target for Bone‐Related Diseases,” Frontiers in Pharmacology 13 (2022): 897539. 35548357 10.3389/fphar.2022.897539 PMC9081430

[12] H. Jiang , J. Yang , T. Li , et al., “JAK/STAT3 signaling in cardiac fibrosis: A promising therapeutic target,” Frontiers in Pharmacology 15 (2024): 1336102. 38495094 10.3389/fphar.2024.1336102 PMC10940489

[13] H. Li , Q. Bi , H. Cui , C. Lv , and M. Wang , “Suppression of autophagy Through JAK2/STAT3 contributes to the therapeutic action of rhynchophylline on asthma,” BMC Complementary Medicine and Therapies 21, no. 1 (2021): 1–12. 33413331 10.1186/s12906-020-03187-w PMC7792286

[14] K. Saito , M. Fujimoto , E. Funajima , et al., “Novel germline STAT3 gain‐of‐function mutation causes autoimmune diseases and severe growth failure,” Journal of Allergy and Clinical Immunology: Global 3, no. 4 (2024): 100312. 39253104 10.1016/j.jacig.2024.100312 PMC11381862

[15] G. N. Zyuz'kov , V. V. Zhdanov , L. A. Miroshnichenko , et al., “The Role of JAK and STAT3 in Regulation of Secretory Function of Neuroglial Cells of Different Types in Ethanol‐Induced Neurodegenerationt,” Bulletin of Experimental Biology and Medicine 172, no. 6 (2022): 686–690. 35501646 10.1007/s10517-022-05457-8

[16] M. El‐Tanani , A. O. Al Khatib , S. M. Aladwan , A. Abuelhana , P. A. McCarron , and M. M. Tambuwala , “Importance of STAT3 signalling in cancer, metastasis and therapeutic interventions,” Cell Signalling 92 (2022): 110275. 35122990 10.1016/j.cellsig.2022.110275

[17] M. M. Kasembeli , E. Kaparos , U. Bharadwaj , et al., “Aberrant function of pathogenic STAT3 mutant proteins is linked to altered stability of monomers and homodimers,” Blood 141, no. 12 (2023): 1411–1424. 36240433 10.1182/blood.2021015330 PMC10651785

[18] S. Manoharan and E. Perumal , “A strategic review of STAT3 signaling inhibition by phytochemicals for cancer prevention and treatment: Advances and insights,” Fitoterapia 179 (2024): 106265. 39437855 10.1016/j.fitote.2024.106265

[19] A. Tesoriere , A. Dinarello , and F. Argenton , “The Roles of Post‐Translational Modifications in STAT3 Biological Activities and Functions,” Biomed 9, no. 8 (2021): 956. 10.3390/biomedicines9080956 PMC8393524 34440160

[20] M. Garg , M. K. Shanmugam , V. Bhardwaj , et al., “The pleiotropic role of transcription factor STAT3 in oncogenesis and its targeting Through natural products for cancer prevention and therapy,” Medicinal Research Reviews 41, no. 3 (2021): 1291–1336. 10.1002/med.21761 33289118

[21] C. E. Heron , L. C. Strowd , and S. R. Feldman , “Janus Kinase (JAK) Inhibitors,” Handbook of Systemic Drug Treatment in Dermatology 170–176, published online February 13, 2023, 10.1201/9781003016786-25.

[22] K. Sardana , S. Bathula , and A. Khurana , “Which is the ideal JAK inhibitor for alopecia areata—Baricitinib, tofacitinib, ritlecitinib or ifidancitinib—Revisiting the immunomechanisms of the JAK pathway,” Indian Dermatology Online Journal 14, no. 4 (2023): 465–474. 37521227 10.4103/idoj.idoj_452_22 PMC10373824

[23] J. Song , J. Wang , S. Tian , and H. Li , “Discovery of STAT3 Inhibitors: Recent Advances and Future Perspectives,” Current Medicinal Chemistry 30, no. 16 (2022): 1824–1847. 10.2174/0929867329666220819093117 35986534

[24] Y. Hua , X. Yuan , Y. H. Shen , et al., “Novel STAT3 Inhibitors Targeting STAT3 Dimerization by Binding to the STAT3 SH2 Domain,” Frontiers in Pharmacology 13 (2022): 836724. 35712699 10.3389/fphar.2022.836724 PMC9196127

[25] O. Kovecses , F. E. Mercier , and M. McKeague , “Nucleic acid therapeutics as differentiation agents for myeloid leukemias,” Leukemia 38, no. 7 (2024): 1441–1454. 38424137 10.1038/s41375-024-02191-0 PMC11216999

[26] Q. Feng and K. Xiao , “Nanoparticle‐Mediated Delivery of STAT3 Inhibitors in the Treatment of Lung Cancer,” Pharmaceutics 14, no. 12 (2022): 2787. 36559280 10.3390/pharmaceutics14122787 PMC9781630

[27] H. Q. Wang , Q. W. Man , F. Y. Huo , et al., “STAT3 pathway in cancers: Past, present, and future,” MedComm 3, no. 2 (2022): e124. 35356799 10.1002/mco2.124 PMC8942302

[28] S. Comità , S. Femmino , C. Thairi , et al., “Regulation of STAT3 and its role in cardioprotection by conditioning: Focus on non‐genomic roles targeting mitochondrial function,” Basic Research in Cardiology 116, no. 1 (2021): 1–31. 34642818 10.1007/s00395-021-00898-0 PMC8510947

[29] K. Wu , Q. Sun , D. Liu , et al., “Alternative Splicing Landscape of Head and Neck Squamous Cell Carcinoma,” Technology in Cancer Research & Treatment 23 (2024). 10.1177/15330338241272051 PMC11307358 39113534

[30] M. Kise , S. Masaki , N. Kataoka , and K. Suzuki , “Identification of Entinostat as a Novel Modifier of STAT3 Pre‐mRNA Alternative Splicing,” Biological & Pharmaceutical Bulletin 47, no. 9 (2024): 1504–1510. 39284734 10.1248/bpb.b24-00404

[31] S. Edtmayer , A. Witalisz‐Siepracka , B. Zdársky , et al., “A novel function of STAT3β in suppressing interferon response improves outcome in acute myeloid leukemia,” Cell Death & Disease 15, no. 5 (2024): 1–12. 38806478 10.1038/s41419-024-06749-9 PMC11133483

[32] J. T. Yu , S. Fan , X. Y. Li , et al., “Novel insights Into STAT3 in renal diseases,” Biomedicine & Pharmacotherapy 165 (2023): 115166. 37473682 10.1016/j.biopha.2023.115166

[33] Y. Hu , Z. Dong , and K. Liu , “Unraveling the complexity of STAT3 in cancer: Molecular understanding and drug discovery,” Journal of Experimental & Clinical Cancer Research 43, no. 1 (2024): 1–29. 38245798 10.1186/s13046-024-02949-5 PMC10799433

[34] X. Hou and F. Tian , “STAT3‐mediated osteogenesis and osteoclastogenesis in osteoporosis,” Cell Communication and Signaling 20, no. 1 (2022): 1–17. 35879773 10.1186/s12964-022-00924-1 PMC9310501

[35] Z. Wu , W. Li , K. Jiang , et al., “Regulation of bone homeostasis: Signaling pathways and therapeutic targets,” MedComm 5, no. 8 (2024): e657. 39049966 10.1002/mco2.657 PMC11266958

[36] M. L. Sobah , C. Liongue , and A. C. Ward , “Contribution of Signal Transducer and Activator of Transcription 3 (STAT3) to Bone Development and Repair,” International Journal of Molecular Sciences 25, no. 1 (2024): 389. 10.3390/ijms25010389 PMC10778865 38203559

[37] X. Yin , Q. Wang , Y. Tang , T. Wang , Y. Zhang , and T. Yu , “Research progress on macrophage polarization During osteoarthritis disease progression: A review,” Journal of Orthopaedic Surgery and Research 19, no. 1 (2024): 584. 39342341 10.1186/s13018-024-05052-9 PMC11437810

[38] J. Li , W. Zhang , X. Liu , et al., “Endothelial Stat3 activation promotes osteoarthritis development,” Cell Proliferation 56, no. 12 (2023): e13518. 37309689 10.1111/cpr.13518 PMC10693181

[39] V. Molnar , E. Pavelić , K. Vrdoljak , et al., “Mesenchymal Stem Cell Mechanisms of Action and Clinical Effects in Osteoarthritis: A Narrative Review,” Genes 13, no. 6 (2022): 949. 35741711 10.3390/genes13060949 PMC9222975

[40] B. Chen , K. Ning , M. L. Sun , and X. A. Zhang , “Regulation and therapy, the role of JAK2/STAT3 signaling pathway in OA: A systematic review,” Cell Communication and Signaling 21, no. 1 (2023): 1–14. 37013568 10.1186/s12964-023-01094-4 PMC10071628

[41] O. Kuryata , O. Akimov , S. Denisenko , et al., “Chondroitin sulfate in osteoarthritis management Among diabetic patients: Molecular mechanisms and clinical potential,” Romanian Journal of Diabetes Nutrition and Metabolic Diseases 30, no. 4 (2023): 481–493.

[42] A. I. S. Jrad , M. Trad , W. Bzeih , G. El Hasbani , and I. Uthman , “Role of pro‐inflammatory interleukins in osteoarthritis: A narrative review,” Connective Tissue Research 64, no. 3 (2023): 238–247. 36541851 10.1080/03008207.2022.2157270

[43] Q. Zhou , Q. Ren , L. Jiao , et al., “The potential roles of JAK/STAT signaling in the progression of osteoarthritis,” Front Endocrinol (Lausanne) 13 (2022): 1069057. 36506076 10.3389/fendo.2022.1069057 PMC9729341

[44] T. Liang , T. Chen , J. Qiu , et al., “Inhibition of nuclear receptor RORα attenuates cartilage damage in osteoarthritis by modulating IL‐6/STAT3 pathway,” Cell Death & Disease 12, no. 10 (2021): 1–13. 34584074 10.1038/s41419-021-04170-0 PMC8478978

[45] G. Dwivedi , L. Flaman , B. Alaybeyoglu , et al., “Inflammatory cytokines and mechanical injury induce post‐traumatic osteoarthritis‐Like changes in a human cartilage‐bone‐synovium microphysiological system,” Arthritis Research & Therapy 24, no. 1 (2022): 1–18. 35982461 10.1186/s13075-022-02881-z PMC9386988

[46] Z. Zou , H. Li , K. Yu , et al., “The potential role of synovial cells in the progression and treatment of osteoarthritis,” Exploration 3, no. 5 (2023): 20220132. 37933282 10.1002/EXP.20220132 PMC10582617

[47] A. Wayupat , P. Kongtawelert , P. Pothacharoen , T. H. Shwe , and T. Phitak , Leptin augments the inflammatory effect of Interleukin 1 Beta on synoviocytes mainly Through NF‐kB and STAT3 prompting its possible implementation in OA pathogenesis, published online October 8, 2024, 10.21203/RS.3.RS-4878390/V1.

[48] J. Zhu , G. Ruan , H. Cen , et al., “Association of serum levels of inflammatory markers and adipokines With joint symptoms and structures in participants With knee osteoarthritis,” Rheumatology 61, no. 3 (2022): 1044–1052. 34114615 10.1093/rheumatology/keab479

[49] J. Xu , L. Yu , F. Liu , L. Wan , and Z. Deng , “The effect of cytokines on osteoblasts and osteoclasts in bone remodeling in osteoporosis: A review,” Frontiers in Immunology 14 (2023): 1222129. 37475866 10.3389/fimmu.2023.1222129 PMC10355373

[50] E. Umur , S. B. Bulut , P. Yiğit , et al., “Exploring the Role of Hormones and Cytokines in Osteoporosis Development,” Biomed 12, no. 8 (2024): 1830. 10.3390/biomedicines12081830 PMC11351445 39200293

[51] W. Da , L. Tao , and Y. Zhu , “The Role of Osteoclast Energy Metabolism in the Occurrence and Development of Osteoporosis,” Frontiers in Endocrinology (Lausanne) 12 (2021): 675385. 10.3389/fendo.2021.675385 PMC8150001 34054735

[52] X. Liu , Z. Zhou , W. N. Zeng , Q. Zeng , and X. Zhang , “The role of toll‐Like receptors in orchestrating osteogenic differentiation of mesenchymal stromal cells and osteoimmunology,” Frontiers in Cell and Developmental Biology 11 (2023): 1277686. 37941898 10.3389/fcell.2023.1277686 PMC10629627

[53] T. Jiang , T. Xia , F. Qiao , N. Wang , Y. Jiang , and H. Xin , “Role and Regulation of Transcription Factors in Osteoclastogenesis,” International Journal of Molecular Sciences 24, no. 22 (2023): 16175. 38003376 10.3390/ijms242216175 PMC10671247

[54] C. H. Li , Z. R. Lü , Z. D. Zhao , et al., “Nitazoxanide, an Antiprotozoal Drug, Reduces Bone Loss in Ovariectomized Mice by Inhibition of RANKL‐Induced Osteoclastogenesis,” Frontiers in Pharmacology 12 (2021): 781640. 34955850 10.3389/fphar.2021.781640 PMC8696474

[55] P. Ethiraj , I. A. Haque , A. K. Alford , et al., “Inhibition of NFAM1 suppresses phospho‐SAPK/JNK signaling During osteoclast differentiation and bone resorption,” Journal of Cellular Biochemistry 122, no. 10 (2021): 1534–1543. 34228377 10.1002/jcb.30076 PMC8479865

[56] J. Xu , W. Jiao , D. B. Wu , et al., “Yishen Tongbi decoction attenuates inflammation and bone destruction in rheumatoid arthritis by regulating JAK/STAT3/SOCS3 pathway,” Frontiers in Immunology 15 (2024): 1381802. 38966637 10.3389/fimmu.2024.1381802 PMC11222394

[57] G. Kour , R. Choudhary , S. Anjum , A. Bhagat , B. K. Bajaj , and Z. Ahmed , “Phytochemicals targeting JAK/STAT pathway in the treatment of rheumatoid arthritis: Is there a future?,” Biochemical Pharmacology 197 (2022): 114929. 35065024 10.1016/j.bcp.2022.114929

[58] N. Kondo , T. Kuroda , and D. Kobayashi , “Cytokine Networks in the Pathogenesis of Rheumatoid Arthritis,” International Journal of Molecular Sciences 22, no. 20 (2021): 10922. 34681582 10.3390/ijms222010922 PMC8539723

[59] S. Liu , H. Ma , H. Zhang , C. Deng , and P. Xin , “Recent advances on signaling pathways and their inhibitors in rheumatoid arthritis,” Clinical Immunology 230 (2021): 108793. 34242749 10.1016/j.clim.2021.108793

[60] D. Ilchovska and D. M. Barrow , “An Overview of the NF‐kB mechanism of pathophysiology in rheumatoid arthritis, investigation of the NF‐kB ligand RANKL and related nutritional interventions,” Autoimmunity Reviews 20, no. 2 (2021): 102741. 33340772 10.1016/j.autrev.2020.102741

[61] Q. Niu , J. Gao , L. Wang , J. Liu , and L. Zhang , “Regulation of differentiation and generation of osteoclasts in rheumatoid arthritis,” Frontiers in Immunology 13 (2022): 1034050. 36466887 10.3389/fimmu.2022.1034050 PMC9716075

[62] G. M. Kim , H. Park , and S. Y. Lee , “Roles of osteoclast‐associated receptor in rheumatoid arthritis and osteoarthritis,” Joint Bone Spine 89, no. 5 (2022): 105400. 35504517 10.1016/j.jbspin.2022.105400

[63] S. Tabrez , M. R. S. Mohammed , N. R. Jabir , and M. I. Khan , “Identification of novel cardiovascular disease associated metabolites using untargeted metabolomics,” Biological Chemistry 402, no. 6 (2021): 749–757. 33951765 10.1515/hsz-2020-0331

[64] S. Tabrez , N. R. Jabir , T. A. Zughaibi , and M. Suhail , “Association of interleukin‐18 promoter polymorphism With comorbid conditions of cardiovascular disease,” Journal of King Saud University 35, no. 1 (2023): 102440.

[65] X. Zhang , S. Chen , G. Yin , et al., “The Role of JAK/STAT Signaling Pathway and Its Downstream Influencing Factors in the Treatment of Atherosclerosis,” Journal of Cardiovascular Pharmacology and Therapeutics 29 (2024). 10.1177/10742484241248046 38656132

[66] Y. Feng , D. Ye , Z. Wang , et al., “The Role of Interleukin‐6 Family Members in Cardiovascular Diseases,” Frontiers in Cardiovascular Medicine 9 (2022): 818890. 35402550 10.3389/fcvm.2022.818890 PMC8983865

[67] A. M. Markin , Y. V. Markina , A. I. Bogatyreva , et al., “The Role of Cytokines in Cholesterol Accumulation in Cells and Atherosclerosis Progression,” International Journal of Molecular Sciences 24, no. 7 (2023): 6426. 37047399 10.3390/ijms24076426 PMC10094347

[68] S. Tabrez , N. R. Jabir , T. A. Zughaibi , and M. Suhail , “Association of IL‐6 promoter polymorphism hotspots (− 174G/C and − 572G/C) With cardiovascular disease risk factors,” Molecular Biology Reports 49, no. 3 (2022): 2265–2272. 35023009 10.1007/s11033-021-07048-8

[69] S. Singh , D. Anshita , and V. Ravichandiran , “MCP‐1: Function, regulation, and involvement in disease,” International Immunopharmacology 101 (2021): 107598. 34233864 10.1016/j.intimp.2021.107598 PMC8135227

[70] T. Xia , M. Zhang , W. Lei , et al., “Advances in the role of STAT3 in macrophage polarization,” Frontiers in Immunology 14 (2023): 1160719. 37081874 10.3389/fimmu.2023.1160719 PMC10110879

[71] X. Liu , J. Liu , Y. Li , and H. Zhang , “The Correlation Between the Inflammatory Effects of Activated Macrophages in Atherosclerosis and Aortic Dissection,” Annals of Vascular Surgery 85 (2022): 341–346. 35395377 10.1016/j.avsg.2022.03.027

[72] X. Sun , J. Gao , X. Meng , X. Lu , L. Zhang , and R. Chen , “Polarized Macrophages in Periodontitis: Characteristics, Function, and Molecular Signaling,” Frontiers in Immunology 12 (2021): 763334. 34950140 10.3389/fimmu.2021.763334 PMC8688840

[73] P. Lin , H. H. Ji , Y. J. Li , and S. D. Guo , “Macrophage Plasticity and Atherosclerosis Therapy,” Frontiers in Molecular Biosciences 8 (2021): 679797. 34026849 10.3389/fmolb.2021.679797 PMC8138136

[74] Y. Wan , L. Mo , H. Huang , et al., “Cadmium contributes to atherosclerosis by affecting macrophage polarization,” Food and Chemical Toxicology 173 (2023): 113603. 36639048 10.1016/j.fct.2023.113603

[75] B. Guo , Y. Yu , M. Wang , et al., “Targeting the JAK2/STAT3 signaling pathway With natural plants and phytochemical ingredients: A novel therapeutic method for combatting cardiovascular diseases,” Biomedicine & Pharmacotherapy 172 (2024): 116313. 38377736 10.1016/j.biopha.2024.116313

[76] W. Li , J. Liu , R. Jiao , et al., “Baricitinib alleviates cardiac fibrosis and inflammation induced by chronic sympathetic activation,” International Immunopharmacology 140 (2024): 112894. 39126736 10.1016/j.intimp.2024.112894

[77] N. V. Dwivedi , S. Datta , K. El‐Kersh , et al., “GPCRs and fibroblast heterogeneity in fibroblast‐associated diseases,” Faseb Journal 37, no. 8 (2023): e23101. 37486603 10.1096/fj.202301091 PMC10916681

[78] S. Perveen , R. Vanni , M. Lo Iacono , R. Rastaldo , and C. Giachino , “Direct Reprogramming of Resident Non‐Myocyte Cells and Its Potential for In Vivo Cardiac Regeneration,” Cells 12, no. 8 (2023): 1166. 37190075 10.3390/cells12081166 PMC10136631

[79] N. J. Patel , D. M. Nassal , D. Gratz , and T. J. Hund , “Emerging therapeutic targets for cardiac arrhythmias: Role of STAT3 in regulating cardiac fibroblast function,” Expert Opinion on Therapeutic Targets 25, no. 1 (2021): 63–73. 33170045 10.1080/14728222.2021.1849145 PMC7856297

[80] S. Lovisa , “Epithelial‐to‐Mesenchymal Transition in Fibrosis: Concepts and Targeting Strategies,” Frontiers in Pharmacology 12 (2021): 737570. 34557100 10.3389/fphar.2021.737570 PMC8454779

[81] Y. Liu , M. Hu , G. Fan , N. Xing , and R. Zhang , “Effect of Baricitinib on the epithelial‐mesenchymal transition of alveolar epithelial cells induced by IL‐6,” International Immunopharmacology 110 (2022): 109044. 35850052 10.1016/j.intimp.2022.109044

[82] M. Aliyu , F. T. Zohora , A. U. Anka , et al., “Interleukin‐6 cytokine: An overview of the immune regulation, immune dysregulation, and therapeutic approach,” International Immunopharmacology 111 (2022): 109130. 35969896 10.1016/j.intimp.2022.109130

[83] J. Zhao , Y. F. Qi , and Y. R. Yu , “STAT3: A key regulator in liver fibrosis,” Annals of Hepatology 21 (2021): 100224. 32702499 10.1016/j.aohep.2020.06.010

[84] A. Shahini and A. Shahini , “Role of interleukin‐6‐mediated inflammation in the pathogenesis of inflammatory bowel disease: Focus on the available therapeutic approaches and gut microbiome,” Journal of Cell Communication and Signaling 17, no. 1 (2022): 55–74. 36112307 10.1007/s12079-022-00695-x PMC10030733

[85] A. A. Nikolskii , I. P. Shilovskiy , E. D. Barvinskaia , A. V. Korneev , M. S. Sundukova , and M. R. Khaitov , “Role of STAT3 Transcription Factor in Pathogenesis of Bronchial Asthma,” Biochemistry 86, no. 11 (2021): 1489–1501. 34906042 10.1134/S0006297921110122

[86] Y. Zhou , X. Huang , H. Yu , et al., “TMT‐based quantitative proteomics revealed protective efficacy of Icariside II Against airway inflammation and remodeling via inhibiting LAMP2, CTSD and CTSS expression in OVA‐induced chronic asthma mice,” Phytomedicine 118 (2023): 154941. 37451150 10.1016/j.phymed.2023.154941

[87] V. Margelidon‐Cozzolino , A. Tsicopoulos , C. Chenivesse , and P. de Nadai , “Role of Th17 Cytokines in Airway Remodeling in Asthma and Therapy Perspectives,” Frontiers in Allergy 3 (2022): 806391. 35386663 10.3389/falgy.2022.806391 PMC8974749

[88] A. Jafarzadeh , P. Chauhan , M. Nemati , S. Jafarzadeh , and A. Yoshimura , “Aberrant expression of suppressor of cytokine signaling (SOCS) molecules contributes to the development of allergic diseases,” Clinical and Experimental Allergy 53, no. 11 (2023): 1147–1161. 37641429 10.1111/cea.14385

[89] A. M. Jetten , J. Y. Beak , A. T. Slominski , and B. Jensen , “Retinoic acid‐related orphan receptor (ror) inverse agonists: Potential therapeutic strategies for multiple inflammatory diseases?,” Nucl Recept Art Sci Modul Des Discov 349–377, published online September 28, 2021, 10.1007/978-3-030-78315-0_14/FIGURES/7.

[90] S. Berry , C. Dossou , A. Kashif , et al., “The role of IL‐17 and anti‐IL‐17 agents in the immunopathogenesis and management of autoimmune and inflammatory diseases,” International Immunopharmacology 102 (2022): 108402. 34863654 10.1016/j.intimp.2021.108402

[91] L. Cui , X. Qin , T. Fu , et al., “Attenuated airways inflammation and remodeling in IL‐37a and IL‐37b transgenic mice With an ovalbumin‐induced chronic asthma,” Cellular Immunology 391‐392 (2023): 104759. 10.1016/j.cellimm.2023.104759 37689011

[92] A. Chetty and H. C. Nielsen , “Targeting airway smooth muscle hypertrophy in asthma: An approach whose time has come,” Journal of Asthma and Allergy 14 (2021): 539–556. 34079293 10.2147/JAA.S280247 PMC8164696

[93] Q. Lu , M. F. Yang , Y. J. Liang , et al., “Immunology of Inflammatory Bowel Disease: Molecular Mechanisms and Therapeutics,” Journal of Inflammation Research 15 (2022): 1825–1844. 35310454 10.2147/JIR.S353038 PMC8928114

[94] L. Wang , Y. Hu , B. Song , Y. Xiong , J. Wang , and D. Chen , “Targeting JAK/STAT signaling pathways in treatment of inflammatory bowel disease,” Inflammation Research 70, no. 7 (2021): 753–764. 34212215 10.1007/s00011-021-01482-x

[95] U. Tripathi , Y. Stern , I. Dagan , et al., “Genetic Overlap Between Inflammatory Bowel Disease and Neurological Disorders: Insights From GWAS and Gene Expression Analysis,” Medrxiv, published online September 26, 2024:2024.07.29.24311160, 10.1101/2024.07.29.24311160.

[96] M. Vebr , R. Pomahačová , J. Sýkora , and J. Schwarz , “A Narrative Review of Cytokine Networks: Pathophysiological and Therapeutic Implications for Inflammatory Bowel Disease Pathogenesis,” Biomed 11, no. 12 (2023): 3229. 10.3390/biomedicines11123229 PMC10740682 38137450

[97] D. Aebisher , D. Bartusik‐Aebisher , A. Przygórzewska , P. Oleś , P. Woźnicki , and A. Kawczyk‐Krupka , “Key Interleukins in Inflammatory Bowel Disease—A Review of Recent Studies,” International Journal of Molecular Sciences 26, no. 1 (2024): 121. 39795980 10.3390/ijms26010121 PMC11719876

[98] W. Zhang , X. Liu , Y. Zhu , et al., “Transcriptional and posttranslational regulation of Th17/Treg balance in health and disease,” European Journal of Immunology 51, no. 9 (2021): 2137–2150. 34322865 10.1002/eji.202048794

[99] J. Mackie , C. S. Ma , S. G. Tangye , and A. Guerin , “The ups and downs of STAT3 function: Too much, too little and human immune dysregulation,” Clinical and Experimental Immunology 212, no. 2 (2023): 107–116. 36652220 10.1093/cei/uxad007 PMC10128169

[100] A. Kondo , S. Ma , M. Y. Y. Lee , et al., “Highly Multiplexed Image Analysis of Intestinal Tissue Sections in Patients With Inflammatory Bowel Disease,” Gastroenterology 161, no. 6 (2021): 1940–1952. 34529988 10.1053/j.gastro.2021.08.055 PMC8606000

[101] P. Robinson , K. Montoya , E. Magness , et al., “Therapeutic Potential of a Small‐Molecule STAT3 Inhibitor in a Mouse Model of Colitis,” Cancers 15, no. 11 (2023): 2977. 37296943 10.3390/cancers15112977 PMC10251885

[102] F. Cordes , E. Lenker , T. Weinhage , et al., “Impaired IFN‐γ‐dependent STAT3 Activation Is Associated With Dysregulation of Regulatory and Inflammatory Signaling in Monocytes of Ulcerative Colitis Patients,” Inflammatory Bowel Diseases 27, no. 6 (2021): 887–901. 33165509 10.1093/ibd/izaa280

[103] Z. Du , A. Chen , L. Huang , et al., “STAT3 couples With 14‐3‐3σ to regulate BCR signaling, B‐cell differentiation, and IgE production,” Journal of Allergy and Clinical Immunology 147, no. 5 (2021): 1907–1923. e6. 33045280 10.1016/j.jaci.2020.09.033

[104] M. Michée‐Cospolite , M. Boudigou , A. Grasseau , et al., “Molecular Mechanisms Driving IL‐10‐ Producing B Cells Functions: STAT3 and c‐MAF as Underestimated Central Key Regulators?,” Frontiers in Immunology 13 (2022): 818814. 35359922 10.3389/fimmu.2022.818814 PMC8961445

[105] S. Vlachiotis and H. Abolhassani , “Transcriptional regulation of B cell class‐switch recombination: The role in development of noninfectious complications,” Expert Review of Clinical Immunology 18, no. 11 (2022): 1145–1154. 36102157 10.1080/1744666X.2022.2123795

[106] A. Witalisz‐Siepracka , K. Klein , B. Zdársky , and D. Stoiber , “The Multifaceted Role of STAT3 in NK‐Cell Tumor Surveillance,” Frontiers in Immunology 13 (2022): 947568. 35865518 10.3389/fimmu.2022.947568 PMC9294167

[107] C. Zalfa and S. Paust , “Natural Killer Cell Interactions With Myeloid Derived Suppressor Cells in the Tumor Microenvironment and Implications for Cancer Immunotherapy,” Frontiers in Immunology 12 (2021): 633205. 34025641 10.3389/fimmu.2021.633205 PMC8133367

[108] R. Pandey , M. Bakay , and H. Hakonarson , “SOCS‐JAK‐STAT inhibitors and SOCS mimetics as treatment options for autoimmune uveitis, psoriasis, lupus, and autoimmune encephalitis,” Frontiers in Immunology 14 (2023): 1271102. 38022642 10.3389/fimmu.2023.1271102 PMC10643230

[109] P. Kotyla , O. Gumkowska‐Sroka , B. Wnuk , and K. Kotyla , “Jak Inhibitors for Treatment of Autoimmune Diseases: Lessons From Systemic Sclerosis and Systemic Lupus Erythematosus,” Pharmaceutics 15, no. 8 (2022): 936. 10.3390/ph15080936 PMC9413112 36015084

[110] T. P. Vogel , J. W. Leiding , M. A. Cooper , and L. R. Forbes Satter , “STAT3 gain‐of‐function syndrome,” Frontiers in Pediatrics 10 (2023): 770077. 36843887 10.3389/fped.2022.770077 PMC9948021

[111] L. Nussbaum , Y. L. Chen , and G. S. Ogg , “Role of regulatory T cells in psoriasis pathogenesis and treatment,” British Journal of Dermatology 184, no. 1 (2021): 14–24. 32628773 10.1111/bjd.19380

[112] P. Hu , M. Wang , H. Gao , et al., “The Role of Helper T Cells in Psoriasis,” Frontiers in Immunology 12 (2021): 788940. 34975883 10.3389/fimmu.2021.788940 PMC8714744

[113] M. Kamata and Y. Tada , “Crosstalk: Keratinocytes and immune cells in psoriasis,” Frontiers in Immunology 14 (2023): 1286344. 38022549 10.3389/fimmu.2023.1286344 PMC10665858

[114] A. Orsmond , L. Bereza‐Malcolm , T. Lynch , L. March , and M. Xue , “Skin Barrier Dysregulation in Psoriasis,” International Journal of Molecular Sciences 22, no. 19 (2021): 10841. 34639182 10.3390/ijms221910841 PMC8509518

[115] M. Kishimoto , M. Komine , M. Sashikawa‐Kimura , et al., “STAT3 Activation in Psoriasis and Cancers,” Diagnostics 11, no. 10 (2021): 1903. 34679602 10.3390/diagnostics11101903 PMC8534757

[116] M. Zhang , N. Li , R. Cai , et al., “Rosmarinic acid protects mice From imiquimod induced psoriasis‐Like skin lesions by inhibiting the IL‐23/Th17 axis via regulating Jak2/Stat3 signaling pathway,” Phytotherapy Research 35, no. 8 (2021): 4526–4537. 34008239 10.1002/ptr.7155

[117] L. I. Ortiz‐Lopez , V. Choudhary , and W. B. Bollag , “Updated Perspectives on Keratinocytes and Psoriasis: Keratinocytes are More Than Innocent Bystanders,” Psoriasis: Targets and Therapy 12 (2022): 73–87. 35529056 10.2147/PTT.S327310 PMC9075909

[118] S. Parab and G. Doshi , “The Experimental Animal Models in Psoriasis Research: A Comprehensive Review,” International Immunopharmacology 117 (2023): 109897. 36822099 10.1016/j.intimp.2023.109897

[119] M. Ramaswamy , R. Tummala , K. Streicher , A. Nogueira da Costa , and P. Z. Brohawn , “The Pathogenesis, Molecular Mechanisms, and Therapeutic Potential of the Interferon Pathway in Systemic Lupus Erythematosus and Other Autoimmune Diseases,” International Journal of Molecular Sciences 22, no. 20 (2021): 11286. 34681945 10.3390/ijms222011286 PMC8540355

[120] M. A. Ameer , H. Chaudhry , J. Mushtaq , et al., “An Overview of Systemic Lupus Erythematosus (SLE) Pathogenesis, Classification, and Management,” Cureus 14, no. 10 (2022): e30330. 36407159 10.7759/cureus.30330 PMC9662848

[121] N. Bolouri , M. Akhtari , E. Farhadi , et al., “Role of the innate and adaptive immune responses in the pathogenesis of systemic lupus erythematosus,” Inflammation Research 71, no. 5 (2022): 537–554. 35298669 10.1007/s00011-022-01554-6

[122] E. Moysidou , M. Christodoulou , G. Lioulios , et al., “Lymphocytes Change Their Phenotype and Function in Systemic Lupus Erythematosus and Lupus Nephritis,” International Journal of Molecular Sciences 25, no. 20 (2024): 10905. 39456692 10.3390/ijms252010905 PMC11508046

[123] S. P. Pandey , R. Bhaskar , S. S. Han , and K. B. Narayanan , “Autoimmune Responses and Therapeutic Interventions for Systemic Lupus Erythematosus: A Comprehensive Review,” Endocrine, Metabolic & Immune Disorders ‐ Drug Targets 24, no. 5 (2023): 499–518. 10.2174/1871530323666230915112642 37718519

[124] T. Montoya , M. L. Castejón , R. Muñoz‐García , and C. Alarcón‐De‐La‐Lastra , “Epigenetic linkage of systemic lupus erythematosus and nutrition,” Nutrition Research Reviews 36, no. 1 (2023): 39–59. 34392862 10.1017/S0954422421000287

[125] C. M. Hedrich , “Epigenetics,” Systemic Lupus Erythematosus: Basic, Applied and Clinical Aspects 277–292, published online January 1, 2021, 10.1016/B978-0-12-814551-7.00032-5.

[126] J. Huang , X. Li , Q. Zhu , M. Wang , Z. Xie , and T. Zhao , “Imbalance of Th17 cells, Treg cells and associated cytokines in patients With systemic lupus erythematosus: A meta‐analysis,” Frontiers in Immunology 15 (2024): 1425847. 39086480 10.3389/fimmu.2024.1425847 PMC11288813

[127] F. C. Paquissi and H. Abensur , “The Th17/IL‐17 Axis and Kidney Diseases, With Focus on Lupus Nephritis,” Frontiers of Medicine 8 (2021): 654912. 10.3389/fmed.2021.654912 PMC8446428 34540858

[128] J. Zhou , B. Lei , F. Shi , et al., “CAR T‐cell therapy for systemic lupus erythematosus: Current status and future perspectives,” Frontiers in Immunology 15 (2024): 1476859. 39749335 10.3389/fimmu.2024.1476859 PMC11694027

[129] T. Zhou , Y. Liu , Z. Yang , et al., “IL‐17 signaling induces iNOS+ microglia activation in retinal vascular diseases,” Glia 69, no. 11 (2021): 2644–2657. 34288126 10.1002/glia.24063

[130] K. F. Azman and R. Zakaria , “Recent Advances on the Role of Brain‐Derived Neurotrophic Factor (BDNF) in Neurodegenerative Diseases,” International Journal of Molecular Sciences 23, no. 12 (2022): 6827. 35743271 10.3390/ijms23126827 PMC9224343

[131] M. Jain , M. K. Singh , H. Shyam , et al., “Role of JAK/STAT in the Neuroinflammation and its Association With Neurological Disorders,” Annals of Neurosciences 28, no. 3‐4 (2021): 191–200. 35341232 10.1177/09727531211070532 PMC8948319

[132] C. G. Hart and S. Karimi‐Abdolrezaee , “Recent insights on astrocyte mechanisms in CNS homeostasis, pathology, and repair,” Journal of Neuroscience Research 99, no. 10 (2021): 2427–2462. 34259342 10.1002/jnr.24922

[133] M. Bhatt , M. Sharma , and B. D. C. Neurobiology , “The Role of Inflammatory Cascade and Reactive Astrogliosis in Glial Scar Formation Post‐spinal Cord Injury,” Springer 44, no. 1 (2024): 78. and M, 2024 undefined. 10.1007/s10571-024-01519-9 PMC11585509 39579235

[134] S. P. Panda , A. Kesharwani , S. Datta , D. Prasanth , S. K. Panda , and A. Guru , “JAK2/STAT3 as a new potential target to manage neurodegenerative diseases: An interactive review,” European Journal of Pharmacology 970 (2024): 176490. 38492876 10.1016/j.ejphar.2024.176490

[135] M. Hoque , A. Samanta , S. S. M. Alam , T. A. Zughaibi , M. A. Kamal , and S. Tabrez , “Nanomedicine‐based immunotherapy for Alzheimer's disease,” Neuroscience and Biobehavioral Reviews 144 (2023): 104973. 36435391 10.1016/j.neubiorev.2022.104973

[136] M. S. Khan , Z. Khan , N. R. Jabir , et al., “Synthesis and Neurobehavioral Evaluation of a Potent Multitargeted Inhibitor for the Treatment of Alzheimer's Disease,” Molecular Neurobiology 62, no. 2 (2024): 1558–1576. 39009798 10.1007/s12035-024-04351-w

[137] S. Thakur , R. Dhapola , P. Sarma , B. Medhi , and D. H. K. Reddy , “Neuroinflammation in Alzheimer's Disease: Current Progress in Molecular Signaling and Therapeutics,” Inflammation 46, no. 1 (2023): 1–17. 35986874 10.1007/s10753-022-01721-1

[138] Z. Fan , W. Zhang , Q. Cao , et al., “JAK2/STAT3 pathway regulates microglia polarization involved in hippocampal inflammatory damage due to acute paraquat exposure,” Ecotoxicology and Environmental Safety 234 (2022): 113372. 35248926 10.1016/j.ecoenv.2022.113372

[139] Y. Zhong , B. Yin , Y. Ye , et al., “The bidirectional role of the JAK2/STAT3 signaling pathway and related mechanisms in cerebral ischemia‐reperfusion injury,” Experimental Neurology 341 (2021): 113690. 33798563 10.1016/j.expneurol.2021.113690

[140] S. Thangwaritorn , C. Lee , E. Metchikoff , et al., “A Review of Recent Advances in the Management of Alzheimer's Disease,” Cureus 16, no. 4 (2024): e58416. 38756263 10.7759/cureus.58416 PMC11098549

[141] W. Wu , Y. Ji , Z. Wang , et al., “The FDA‐approved anti‐amyloid‐β monoclonal antibodies for the treatment of Alzheimer's disease: A systematic review and meta‐analysis of randomized controlled trials,” European Journal of Medical Research 28, no. 1 (2023): 1–13. 38017568 10.1186/s40001-023-01512-w PMC10683264

[142] C. P. Chang , C. W. Wu , and Y. Chern , “Metabolic dysregulation in Huntington's disease: Neuronal and glial perspectives,” Neurobiology of Disease 201 (2024): 106672. 39306013 10.1016/j.nbd.2024.106672

[143] X. Li , H. Tong , S. Xu , et al., “Neuroinflammatory Proteins in Huntington's Disease: Insights Into Mechanisms, Diagnosis, and Therapeutic Implications,” International Journal of Molecular Sciences 25, no. 21 (2024): 11787. 39519337 10.3390/ijms252111787 PMC11546928

[144] Q. Jia , S. Li , X. J. Li , and P. Yin , “Neuroinflammation in Huntington's disease: From animal models to clinical therapeutics,” Frontiers in immunology 13 (2022): 1088124. 36618375 10.3389/fimmu.2022.1088124 PMC9815700

[145] L. Abjean , H. L. Ben , M. Riquelme‐Perez , et al., “Reactive astrocytes promote proteostasis in Huntington's disease Through the JAK2‐STAT3 pathway,” Brain 146, no. 1 (2023): 149–166. 35298632 10.1093/brain/awac068

[146] K. Vaibhav , M. Ahluwalia , P. Gaur , and J. Luo , “TGF‐β as a Key Modulator of Astrocyte Reactivity: Disease Relevance and Therapeutic Implications,” Biomed 10, no. 5 (2022): 1206. 10.3390/biomedicines10051206 PMC9138510 35625943

[147] S. A. Liddelow , M. L. Olsen , and M. V. Sofroniew , “Reactive Astrocytes and Emerging Roles in Central Nervous System (CNS) Disorders,” Cold Spring Harbor Perspectives in Biology 16, no. 7 (2024): a041356. 38316554 10.1101/cshperspect.a041356 PMC11216178

[148] Z. He , B. Song , M. Zhu , and J. Liu , “Comprehensive pan‐cancer analysis of STAT3 as a prognostic and immunological biomarker,” Scientific Reports 13, no. 1 (2023): 1–18. 36977736 10.1038/s41598-023-31226-2 PMC10050087

[149] B. Panda , A. Tripathy , S. Patra , B. Kullu , S. Tabrez , and M. Jena , “Imperative connotation of SODs in cancer: Emerging targets and multifactorial role of action,” Iubmb Life, published online 2024, 10.1002/IUB.2821. 38600696

[150] M. You , Z. Xie , N. Zhang , et al., “Signaling pathways in cancer metabolism: Mechanisms and therapeutic targets,” Signal Transduction and Targeted Therapy 8, no. 1 (2023): 1–27. 37164974 10.1038/s41392-023-01442-3 PMC10172373

[151] Y. Wang , Z. Wang , S. Li , J. Ma , X. Dai , and J. Lu , “Deciphering JAK/STAT signaling pathway: A multifaceted approach to tumorigenesis, progression and therapeutic interventions,” International Immunopharmacology 131 (2024): 111846. 38520787 10.1016/j.intimp.2024.111846

[152] T. M. Ayele , Z. T. Muche , A. B. Teklemariam , A. B. Kassie , and E. C. Abebe , “Role of JAK2/STAT3 Signaling Pathway in the Tumorigenesis, Chemotherapy Resistance, and Treatment of Solid Tumors: A Systemic Review,” Journal of Inflammation Research 15 (2022): 1349–1364. 35241923 10.2147/JIR.S353489 PMC8887966

[153] O. Rahbar Farzam , S. Najafi , M. Amini , et al., “Interplay of miRNAs and lncRNAs in STAT3 signaling pathway in colorectal cancer progression,” Cancer Cell International 24, no. 1 (2024): 1–13. 38185635 10.1186/s12935-023-03202-3 PMC10771635

[154] C. Xue , Q. Yao , X. Gu , et al., “Evolving cognition of the JAK‐STAT signaling pathway: Autoimmune disorders and cancer,” Signal Transduction and Targeted Therapy 8, no. 1 (2023): 1–24. 37208335 10.1038/s41392-023-01468-7 PMC10196327

[155] Y. X. Liu , B. W. Xu , X. D. Niu , et al., “Inhibition of Src/STAT3 signaling‐mediated angiogenesis is involved in the anti‐melanoma effects of dioscin,” Pharmacological Research 175 (2022): 105983. 34822972 10.1016/j.phrs.2021.105983

[156] W. Zhang , D. Li , B. Li , X. Chu , and B. Kong , “STAT3 as a therapeutic target in the metformin‐related treatment,” International Immunopharmacology 116 (2023): 109770. 36746021 10.1016/j.intimp.2023.109770

[157] M. Hashemi , E. Sabouni , P. Rahmanian , et al., “Deciphering STAT3 signaling potential in hepatocellular carcinoma: Tumorigenesis, treatment resistance, and pharmacological significance,” Cellular & Molecular Biology Letters 28, no. 1 (2023): 1–32. 37085753 10.1186/s11658-023-00438-9 PMC10122325

[158] S. Guo , V. Ramar , A. A. Guo , et al., “TRPM7 transactivates the FOSL1 gene Through STAT3 and enhances glioma stemness,” Cellular and Molecular Life Sciences 80, no. 9 (2023): 1–18. 37642779 10.1007/s00018-023-04921-6 PMC10465393

[159] J. E. Lefler , C. B. MarElia‐Bennett , K. A. Thies , et al., “STAT3 in tumor fibroblasts promotes an immunosuppressive microenvironment in pancreatic cancer,” Life Science Alliance 5, no. 11 (2022). 10.26508/lsa.202201460 PMC9270499 35803738

[160] M. Sadrkhanloo , M. D. A. Paskeh , M. Hashemi , et al., “STAT3 signaling in prostate cancer progression and therapy resistance: An oncogenic pathway With diverse functions,” Biomedicine & Pharmacotherapy 158 (2023): 114168. 36916439 10.1016/j.biopha.2022.114168

[161] L. M. Channon , V. M. Tyma , Z. Xu , et al., “Small extracellular vesicles (exosomes) and their cargo in pancreatic cancer: Key roles in the hallmarks of cancer,” Biochimica et Biophysica Acta—Review Cancer 1877, no. 3 (2022): 188728. 10.1016/j.bbcan.2022.188728 35385773

[162] M. Golmohammadi , M. Y. Zamanian , A. M. Al‐Ani , et al., “Targeting STAT3 signaling pathway by curcumin and its analogues for breast cancer: A narrative review,” Animal Models and Experimental Medicine 7, no. 6 (2024): 853–867. 39219410 10.1002/ame2.12491 PMC11680487

[163] S. Dimri , R. Malhotra , T. Shet , S. Mokal , S. Gupta , and A. De , “Noncanonical pS727 post translational modification dictates major STAT3 activation and downstream functions in breast cancer,” Experimental Cell Research 396, no. 2 (2020): 112313. 33002501 10.1016/j.yexcr.2020.112313

[164] Y. J. Li , C. Zhang , A. Martincuks , A. Herrmann , and H. Yu , “STAT proteins in cancer: Orchestration of metabolism,” Nature Reviews Cancer 23, no. 3 (2023): 115–134. 36596870 10.1038/s41568-022-00537-3

[165] S. G. Manore , D. L. Doheny , G. L. Wong , and H. W. Lo , “IL‐6/JAK/STAT3 Signaling in Breast Cancer Metastasis: Biology and Treatment,” Frontiers in Oncology 12 (2022): 866014. 35371975 10.3389/fonc.2022.866014 PMC8964978

[166] X. Yang and H. Wu , “RAS signaling in carcinogenesis, cancer therapy and resistance mechanisms,” Journal of Hematology & Oncology 17, no. 1 (2024): 1–31. 39522047 10.1186/s13045-024-01631-9 PMC11550559

[167] L. Raji , A. Tetteh , and A. Amin , “Role of c‐Src in Carcinogenesis and Drug Resistance,” Cancers 16, no. 1 (2023): 32. 38201459 10.3390/cancers16010032 PMC10778207

[168] Y. Ma , Y. Zhu , L. Shang , et al., “LncRNA XIST regulates breast cancer stem cells by activating proinflammatory IL‐6/STAT3 signaling,” Oncogene 42, no. 18 (2023): 1419–1437. 36922677 10.1038/s41388-023-02652-3 PMC10154203

[169] R. A. Eladwy , H. T. Vu , R. Shah , C. G. Li , D. Chang , and D. J. Bhuyan , “The Fight Against the Carcinogenic Epstein‐Barr Virus: Gut Microbiota, Natural Medicines, and Beyond,” International Journal of Molecular Sciences 24, no. 2 (2023): 1716. 36675232 10.3390/ijms24021716 PMC9862477

[170] J. Schwestermann , A. Besse , C. Driessen , and L. Besse , “Contribution of the Tumor Microenvironment to Metabolic Changes Triggering Resistance of Multiple Myeloma to Proteasome Inhibitors,” Frontiers in Oncology 12 (2022): 899272. 35692781 10.3389/fonc.2022.899272 PMC9178120

[171] B. Gutic , T. Bozanovic , A. Mandic , et al., “Programmed cell death‐1 and its ligands: Current knowledge and possibilities in immunotherapy,” Clinics 78 (2023): 100177. 36931099 10.1016/j.clinsp.2023.100177 PMC10025950

[172] O. Aksoy , J. Lind , V. Sunder‐Plaßmann , S. Vallet , and K. Podar , “Bone marrow microenvironment‐ induced regulation of Bcl‐2 family members in multiple myeloma (MM): Therapeutic implications,” Cytokine 161 (2023): 156062. 36332463 10.1016/j.cyto.2022.156062

[173] L. Cai , Y. Wang , H. Chen , et al., “Platinum(IV) Complexes as Inhibitors of STAT3 and Regulators of the Tumor Microenvironment To Control Breast Cancer,” Journal of Medicinal Chemistry 66, no. 16 (2023): 11351–11364. 37578941 10.1021/acs.jmedchem.3c00836

[174] K. A. F. Pennel , P. Hatthakarnkul , C. S. Wood , et al., “JAK/STAT3 represents a therapeutic target for colorectal cancer patients With stromal‐rich tumors,” Journal of Experimental & Clinical Cancer Research 43, no. 1 (2024): 1–20. 38424636 10.1186/s13046-024-02958-4 PMC10905886

[175] H. S. Tuli , K. Sak , A. Iqubal , et al., “STAT signaling as a target for intervention: From cancer inflammation and angiogenesis to non‐coding RNAs modulation,” Molecular Biology Reports 49, no. 9 (2022): 8987–8999. *2022 499*. 35474053 10.1007/s11033-022-07399-w

[176] Y. Zhong , L. Deng , S. Shi , et al., “The novel STAT3 inhibitor WZ‐2‐033 causes regression of human triple‐negative breast cancer and gastric cancer xenografts,” Acta Pharmacologica Sinica no. 4 (2021): 1013–1023. *2021 434*. 34267347 10.1038/s41401-021-00718-0 PMC8976066

[177] M. Chen , T. Wang , D. Tian , C. Hai , and Z. Qiu , “Induction, growth, drug resistance, and metastasis: A comprehensive summary of the relationship Between STAT3 and gastric cancer,” Heliyon 10, no. 18 (2024). 10.1016/j.heliyon.2024.e37263 PMC11416542 39309860

[178] D. Standing , E. Feess , S. Kodiyalam , et al., “The Role of STATs in Ovarian Cancer: Exploring Their Potential for Therapy,” Cancers 2023, no. 9: 2485. 10.3390/cancers15092485 PMC10177275 37173951

[179] Y. Wang , J. Wang , C. Yang , et al., “A study of the correlation Between M2 macrophages and lymph node metastasis of colorectal carcinoma,” World Journal of Surgical Oncology 19, no. 1 (2021): 1–8. 33781288 10.1186/s12957-021-02195-5 PMC8008636

[180] A. N. Gargalionis , K. A. Papavassiliou , and A. G. Papavassiliou , “Targeting STAT3 Signaling Pathway in Colorectal Cancer,” Biomed 9, no. 8 (2021): 1016. 10.3390/biomedicines9081016 PMC8392110 34440220

[181] S. Shi , H. Y. Ma , and Z. G. Zhang , “Clinicopathological and prognostic value of STAT3/p‐STAT3 in cervical cancer: A meta and bioinformatics analysis,” Pathology ‐ Research and Practice 227 (2021): 153624. 34571355 10.1016/j.prp.2021.153624

[182] Y. Zhang , W. Lu , Y. Chen , et al., “The miR‐19b‐3p‐MAP2K3‐STAT3 feedback loop regulates cell proliferation and invasion in esophageal squamous cell carcinoma,” Molecular Oncology 15, no. 5 (2021): 1566–1583. 33660414 10.1002/1878-0261.12934 PMC8096789

[183] Z. Qureshy , H. Li , Y. Zeng , et al., “STAT3 Activation as a Predictive Biomarker for Ruxolitinib Response in Head and Neck Cancer,” Clinical Cancer Research 28, no. 21 (2022): 4737–4746. 35929989 10.1158/1078-0432.CCR-22-0744 PMC10024606

[184] H. Y. Lim , P. S. Ong , L. Wang , et al., “Celastrol in cancer therapy: Recent developments, challenges and prospects,” Cancer Letters 521 (2021): 252–267. 34508794 10.1016/j.canlet.2021.08.030

[185] Y. Kang , H. Li , Y. Liu , and Z. Li , “Regulation of VEGF‐A expression and VEGF‐A‐targeted therapy in malignant tumors,” Journal of Cancer Research and Clinical Oncology 150, no. 5 (2024): 1–10. 10.1007/s00432-024-05714-5 PMC11061008 38687357

[186] M. Ashrafizadeh , C. D. Mohan , S. Rangappa , et al., “Noncoding RNAs as regulators of STAT3 pathway in gastrointestinal cancers: Roles in cancer progression and therapeutic response,” Medicinal Research Reviews 43, no. 5 (2023): 1263–1321. 36951271 10.1002/med.21950

[187] B. Kumar , A. S. Khatpe , J. Guanglong , et al., “Stromal heterogeneity may explain increased incidence of metaplastic breast cancer in women of African descent,” Nature Communications 14, no. 1 (2023): 1–22. 10.1038/s41467-023-41473-6 PMC10502140 37709737

[188] L. E. Pascal , Y. Wang , M. Zhong , et al., “EAF2 and p53 Co‐Regulate STAT3 Activation in Prostate Cancer,” Neoplasia 20, no. 4 (2018): 351–363. 29518696 10.1016/j.neo.2018.01.011 PMC5909677

[189] W. Peng , H. Zhang , M. Yin , et al., “Combined Inhibition of PI3K and STAT3 signaling effectively inhibits bladder cancer growth,” Oncogenesis 13, no. 1 (2024): 1–12. 39068158 10.1038/s41389-024-00529-y PMC11283499

[190] S. Singh , H. J. Gomez , S. Thakkar , S. P. Singh , and A. S. Parihar , “Overcoming Acquired Drug Resistance to Cancer Therapies Through Targeted STAT3 Inhibition,” International Journal of Molecular Sciences 24, no. 5 (2023): 4722. 36902166 10.3390/ijms24054722 PMC10002572

[191] F. Khan , P. Pandey , M. Verma , and T. K. Upadhyay , “Terpenoid‐Mediated Targeting of STAT3 Signaling in Cancer: An Overview of Preclinical Studies,” Biomol 14, no. 2 (2024): 200. 10.3390/biom14020200 PMC10886526 38397437

[192] M. Sadrkhanloo , M. Entezari , S. Orouei , et al., “STAT3‐EMT axis in tumors: Modulation of cancer metastasis, stemness and therapy response,” Pharmacological Research 182 (2022): 106311. 35716914 10.1016/j.phrs.2022.106311

[193] Z. Ma , F. Zhou , H. Jin , and X. Wu , “Crosstalk Between CXCL12/CXCR4/ACKR3 and the STAT3 Pathway,” Cells 13, no. 12 (2024): 1027. 38920657 10.3390/cells13121027 PMC11201928

[194] R. Y. Jiang , J. Y. Zhu , H. P. Zhang , et al., “STAT3: Key targets of growth‐promoting receptor positive breast cancer,” Cancer Cell International 24, no. 1 (2024): 1–41. 39468521 10.1186/s12935-024-03541-9 PMC11520424

[195] Z. Kang , S. Li , Y. Li , J. Song , Y. Peng , and Y. Chen , “Small molecular inhibitors and degraders targeting STAT3 for cancer therapy: An updated review (From 2022 to 2024),” Chinese Chemical Letters 110447, published online September 11, 2024, 10.1016/J.CCLET.2024.110447.

[196] T. Adesoye , D. Tripathy , K. K. Hunt , and K. Keyomarsi , “Exploring Novel Frontiers: Leveraging STAT3 Signaling for Advanced Cancer Therapeutics,” Cancers 16, no. 3 (2024): 492. 38339245 10.3390/cancers16030492 PMC10854592

[197] S. S. Yu , R. C. Tang , A. Zhang , et al., “Deacetylase Sirtuin 1 mitigates type I IFN‐ and type II IFN‐induced signaling and antiviral immunity,” Journal of Virology 98, no. 3 (2024). 10.1128/jvi.00088-24 PMC10949466 38386781

[198] S. Hashimoto , A. Hashimoto , R. Muromoto , Y. Kitai , K. Oritani , and T. Matsuda , “Central Roles of STAT3‐Mediated Signals in Onset and Development of Cancers: Tumorigenesis and Immunosurveillance,” Cells 11, no. 16 (2022): 2618. 36010693 10.3390/cells11162618 PMC9406645

[199] X. Hu , J. li , M. Fu , X. Zhao , and W. Wang , “The JAK/STAT signaling pathway: From bench to clinic,” Signal Transduction and Targeted Therapy 6, no. 1 (2021): 1–33. 34824210 10.1038/s41392-021-00791-1 PMC8617206

[200] J. Xu , H. Lin , G. Wu , M. Zhu , and M. Li , “IL‐6/STAT3 Is a Promising Therapeutic Target for Hepatocellular Carcinoma,” Frontiers in Oncology 11 (2021): 760971. 34976809 10.3389/fonc.2021.760971 PMC8714735

[201] S. Rose‐John , B. J. Jenkins , C. Garbers , J. M. Moll , and J. Scheller , “Targeting IL‐6 trans‐signalling: Past, present and future prospects,” Nature Reviews Immunology 23, no. 10 (2023): 666–681. 10.1038/s41577-023-00856-y PMC10108826 37069261

[202] S. Natani , V. M. Dhople , A. Parveen , et al., “AMPK/SIRT1 signaling Through p38MAPK mediates Interleukin‐6 induced neuroendocrine differentiation of LNCaP prostate cancer cells,” Biochimica et Biophysica Acta—Molecular Cell Research 1868, no. 10 (2021): 119085. 34171447 10.1016/j.bbamcr.2021.119085

[203] L. Puigdevall , C. Michiels , C. Stewardson , and L. Dumoutier , “JAK/STAT: Why choose a classical or an alternative pathway when you can have both?,” Journal of Cellular and Molecular Medicine 26, no. 7 (2022): 1865–1875. 35238133 10.1111/jcmm.17168 PMC8980962

[204] S. Sudhesh Dev , S. A. Zainal Abidin , R. Farghadani , I. Othman , and R. Naidu , “Receptor Tyrosine Kinases and Their Signaling Pathways as Therapeutic Targets of Curcumin in Cancer,” Frontiers in Pharmacology 12 (2021): 772510. 34867402 10.3389/fphar.2021.772510 PMC8634471

[205] S. Luo , S. Du , M. Tao , J. Cao , and P. Cheng , “Insights on hematopoietic cell kinase: An oncogenic player in human cancer,” Biomedicine & Pharmacotherapy 160 (2023): 114339. 36736283 10.1016/j.biopha.2023.114339

[206] M. Cao , Y. Wang , G. Lu , et al., “Classical Angiogenic Signaling Pathways and Novel Anti‐Angiogenic Strategies for Colorectal Cancer,” Current Issues in Molecular Biology 44, no. 10 (2022): 4447–4471. 36286020 10.3390/cimb44100305 PMC9601273

[207] Z. Yang , X. Zhang , X. Bai , X. Xi , W. Liu , and W. Zhong , “Anti‐angiogenesis in colorectal cancer therapy,” Cancer Science 115, no. 3 (2024): 734–751. 38233340 10.1111/cas.16063 PMC10921012

[208] Q. B. Liu , R. H. Zhou , and C. M. Liu , “TLR9/FCRL3 regulates B cell viability, apoptosis, and antibody and IL‐10 production Through ERK1/2, p38, and STAT3 signaling pathways,” In Vitro Cellular & Developmental Biology—Animal 58, no. 8 (2022): 702–711. 36121575 10.1007/s11626-022-00720-8

[209] A. H. Rahmani , A. Almatroudi , K. S. Allemailem , et al., “Myricetin: A Significant Emphasis on Its Anticancer Potential via the Modulation of Inflammation and Signal Transduction Pathways,” International Journal of Molecular Sciences 24, no. 11 (2023): 9665. 37298616 10.3390/ijms24119665 PMC10253333

[210] M. Ashrafizadeh , M. H. Gholami , S. Mirzaei , et al., “Dual relationship Between long non‐coding RNAs and STAT3 signaling in different cancers: New insight to proliferation and metastasis,” Life Sciences 270 (2021): 119006. 33421521 10.1016/j.lfs.2020.119006

[211] M. Sajjadi‐Dokht , T. A. Merza Mohamad , H. Sulaiman Rahman , et al., “MicroRNAs and JAK/STAT3 signaling: A new promising therapeutic axis in blood cancers,” Genes & Diseases 9, no. 4 (2022): 849–867. 35685482 10.1016/j.gendis.2021.10.009 PMC9170603

[212] Y. L. Chen , C. C. Hsieh , P. M. Chu , J. Y. Chen , Y. C. Huang , and C. Y. Chen , “Roles of protein tyrosine phosphatases in hepatocellular carcinoma progression (Review),” Oncology Reports 49, no. 3 (2023). 10.3892/or.2023.8485 PMC9887465 36660927

[213] Y. Lin , Z. Xiaohan , K. Yang , et al., “Protein tyrosine phosphatase receptor type D gene promotes radiosensitivity via STAT3 dephosphorylation in nasopharyngeal carcinoma,” Oncogene 40 (2021): 3101–3117. 33824475 10.1038/s41388-021-01768-8 PMC8084736

[214] K. A. Young , K. Wojdyla , T. Lai , et al., “The receptor protein tyrosine phosphatase PTPRK promotes intestinal repair and catalysis‐independent tumor suppression,” Journal of Cell Science 137, no. 14 (2024). 10.1242/jcs.261914 PMC11298714 38904097

[215] X. Tang , X. Sui , and Y. Liu , “Immune checkpoint PTPN2 predicts prognosis and immunotherapy response in human cancers,” Heliyon 9, no. 1 (2023). 10.1016/j.heliyon.2023.e12873 PMC9852697 36685446

[216] J. Song , J. Lan , J. Tang , and N. Luo , “PTPN2 in the Immunity and Tumor Immunotherapy: A Concise Review,” International Journal of Molecular Sciences 23, no. 17 (2022): 10025. 36077422 10.3390/ijms231710025 PMC9456094

[217] L. Dai , Z. Li , W. Liang , et al., “SOCS proteins and their roles in the development of glioblastoma,” Oncology Letters 23, no. 1 (2022). 10.3892/ol.2021.13123 PMC8607235 34820004

[218] H. Yoshikawa , K. Matsubara , G. S. Qian , et al., “SOCS‐1, a negative regulator of the JAK/STAT pathway, is silenced by methylation in human hepatocellular carcinoma and shows growth‐suppression activity,” Nature Genetics 28 (2001): 29–35, Accessed May 4, 2024, http://genetics.nature.com. 11326271 10.1038/ng0501-29

[219] M. Morelli , S. Madonna , and C. Albanesi , “SOCS1 and SOCS3 as key checkpoint molecules in the immune responses associated to skin inflammation and malignant transformation,” Frontiers in Immunology 15 (2024): 1393799. 38975347 10.3389/fimmu.2024.1393799 PMC11224294

[220] W. Blaszczak , B. White , S. Monterisi , and P. Swietach , “Dynamic IL‐6R/STAT3 signaling leads to heterogeneity of metabolic phenotype in pancreatic ductal adenocarcinoma cells,” Cell Reports 43, no. 1 (2024). 10.1016/j.celrep.2023.113612 PMC11149489 38141171

[221] C. Yu , Y. Fan , Y. Zhang , L. Liu , and G. Guo , “LINC00893 inhibits the progression of prostate cancer Through miR‐3173‐5p/SOCS3/JAK2/STAT3 pathway,” Cancer Cell International 22, no. 1 (2022): 1–17. 35818076 10.1186/s12935-022-02637-4 PMC9275192

[222] L. Dai , Y. Han , Z. Yang , et al., “Identification and validation of SOCS1/2/3/4 as potential prognostic biomarkers and correlate With immune infiltration in glioblastoma,” Journal of Cellular and Molecular Medicine 27, no. 15 (2023): 2194–2214. 37315184 10.1111/jcmm.17807 PMC10399539

[223] X. Li , A. Rasul , F. Sharif , and M. Hassan , “PIAS family in cancer: From basic mechanisms to clinical applications,” Frontiers in Oncology 14 (2024): 1376633. 38590645 10.3389/fonc.2024.1376633 PMC10999569

[224] Q. Ju , Q. Shi , C. Liu , G. Fu , and H. Shi , “Bufalin suppresses esophageal squamous cell carcinoma progression by activating the PIAS3/STAT3 signaling pathway,” Journal of Thoracic Disease 15, no. 4 (2023): 2141–2160. 37197494 10.21037/jtd-23-486 PMC10183519

[225] M. Jiang , W. Zhang , R. Zhang , et al., “Cancer exosome‐derived miR‐9 and miR‐181a promote the development of early‐stage MDSCs via interfering With SOCS3 and PIAS3 respectively in breast cancer,” Oncogene 39 (2020): 4681–4694. 32398867 10.1038/s41388-020-1322-4

[226] W. Bao , J. Wang , K. Fan , Y. Gao , and J. Chen , “PIAS3 promotes ferroptosis by regulating TXNIP via TGF‐β signaling pathway in hepatocellular carcinoma,” Pharmacological Research 196 (2023): 106915. 37689128 10.1016/j.phrs.2023.106915

[227] Y. Fei , X. Zhang , X. Wang , et al., “Upregulation of tumor suppressor PIAS3 by Honokiol promotes tumor cell apoptosis via selective inhibition of STAT3 tyrosine 705 phosphorylation,” Journal of Natural Medicines 78, no. 2 (2024): 285–295. 38082192 10.1007/s11418-023-01757-z

[228] H. Chen , W. Zhou , A. Bian , et al., “Selectively Targeting STAT3 Using a Small Molecule Inhibitor is a Potential Therapeutic Strategy for Pancreatic Cancer,” Clinical Cancer Research 29, no. 4 (2023): 815–830. 36374556 10.1158/1078-0432.CCR-22-0997

[229] M. A. Kamal , Y. M. Mandour , M. K. Abd El‐Aziz , U. Stein , and H. M. El Tayebi , “Small Molecule Inhibitors for Hepatocellular Carcinoma: Advances and Challenges,” Mol 27, no. 17 (2022): 5537. 10.3390/molecules27175537 PMC9457820 36080304

[230] S. Manoharan , A. Balakrishnan , V. Hemamalini , and E. Perumal , “Screening of potent STAT3‐SH2 domain inhibitors From JAK/STAT compound library Through molecular dynamics simulation,” Molecular Diversity 27, no. 3 (2023): 1297–1308. 35831728 10.1007/s11030-022-10490-w

[231] C. C. Zhang , T. Wu , L. Guan , et al., “Effects of STAT3 Inhibitor BP‐1‐102 on The Proliferation, Invasiveness, Apoptosis and Neurosphere Formation of Glioma Cells in Vitro,” Cell Biochemistry and Biophysics 80, no. 4 (2022): 723–735. 35994220 10.1007/s12013-022-01088-y

[232] H. Guo , Y. Xiao , Z. Yuan , et al., “Inhibition of STAT3Y705 phosphorylation by Stattic suppresses proliferation and induces mitochondrial‐dependent apoptosis in pancreatic cancer cells,” Cell Death Discovery 8, no. 1 (2022): 1–12. 35288541 10.1038/s41420-022-00922-9 PMC8921333

[233] S. Imbaby and Y. Hattori , “Stattic ameliorates the cecal ligation and puncture‐induced cardiac injury in septic mice via IL‐6‐gp130‐STAT3 signaling pathway,” Life Sciences 330 (2023): 122008. 37549828 10.1016/j.lfs.2023.122008

[234] L. Pan , X. Chen , F. V. Rassool , C. Li , and J. Lin , “LLL12B, a Novel Small‐Molecule STAT3 Inhibitor, Induces Apoptosis and Suppresses Cell Migration and Tumor Growth in Triple‐Negative Breast Cancer Cells,” Biomed 10, no. 8 (2022): 2003. 10.3390/biomedicines10082003 PMC9405793 36009550

[235] S. M. Zarezadeh , A. M. Sharafi , G. Erabi , et al., “Natural STAT3 Inhibitors for Cancer Treatment: A Comprehensive Literature Review,” Recent Patents on Anti‐Cancer Drug Discovery 19, no. 4 (2023): 403–502. 10.2174/1574892818666230803100554 37534488

[236] X. Zhang , T. Pang , H. Zhang , et al., “The natural compound periplogenin suppresses the growth of prostate carcinoma cells by directly targeting ATP1A1,” Science Reports 14, no. 1 (2024): 1–8. 10.1038/s41598-024-71722-7 PMC11372130 39227746

[237] P. He , Y. Miao , Y. Sun , et al., “Discovery of a Novel Potent STAT3 Inhibitor HP590 With Dual p‐Tyr705/Ser727Inhibitory Activity for Gastric Cancer Treatment,” Journal of Medicinal Chemistry 65, no. 19 (2022): 12650–12674. 36103247 10.1021/acs.jmedchem.2c00413

[238] B. Biersack and M. Höpfner , “Emerging role of MYB transcription factors in cancer drug resistance,” Cancer Drug Resistance 7 (2024): 15. 38835346 10.20517/cdr.2023.158 PMC11149108

[239] X. Gu , T. Zhang , T. Liu , H. Cong , and W. Wu , “Cirsilineol inhibits the proliferation and migration of endometriotic cells,” Tropical Journal of Pharmaceutical Research 22, no. 12 (2023): 2427–2432.

[240] V. R. Silva , L. S. de Santos , R. B. Dias , C. A. Quadros , and D. P. Bezerra , “Emerging agents that target signaling pathways to eradicate colorectal cancer stem cells,” Cancer Communications 41, no. 12 (2021): 1275–1313. 34791817 10.1002/cac2.12235 PMC8696218

[241] Q. Feng and K. Xiao , “Nanoparticle‐Mediated Delivery of STAT3 Inhibitors in the Treatment of Lung Cancer,” Pharmaceutics 14, no. 12 (2022): 2787. 36559280 10.3390/pharmaceutics14122787 PMC9781630

[242] C. Hervieu , N. Christou , S. Battu , and M. Mathonnet , “The Role of Cancer Stem Cells in Colorectal Cancer: From the Basics to Novel Clinical Trials,” Cancers 13, no. 5 (2021): 1092. 33806312 10.3390/cancers13051092 PMC7961892

[243] M. Zhu , S. Li , X. Cao , K. Rashid , and T. Liu , “The STAT family: Key transcription factors mediating crosstalk Between cancer stem cells and tumor immune microenvironment,” Seminars in Cancer Biology 88 (2023): 18–31. 36410636 10.1016/j.semcancer.2022.11.011

[244] H. Flebbe , M. Spitzner , P. E. Marquet , et al., “Targeting STAT3 Signaling Facilitates Responsiveness of Pancreatic Cancer Cells to Chemoradiotherapy,” Cancers (Basel) 14, no. 5 (2022): 1301. 35267609 10.3390/cancers14051301 PMC8908974

[245] D. Pádua , P. Figueira , I. Ribeiro , R. Almeida , and P. Mesquita , “The Relevance of Transcription Factors in Gastric and Colorectal Cancer Stem Cells Identification and Eradication,” Frontiers in Cell and Developmental Biology 8 (2020): 544473. 10.3389/fcell.2020.00442 PMC7314965 32626705

[246] S. Y. Ye , J. Y. Li , T. H. Li , et al., “The Efficacy and Safety of Celecoxib in Addition to Standard Cancer Therapy: A Systematic Review and Meta‐Analysis of Randomized Controlled Trials,” Current Oncology Reports 29, no. 9 (2022): 6137–6153. 10.3390/curroncol29090482 PMC9497539 36135051

[247] T. Hu , C. J. Liu , X. Yin , et al., “Selective COX‐2 inhibitors do not increase gastrointestinal reactions After colorectal cancer surgery: A systematic review and meta‐analysis,” BMC Gastroenterology [Electronic Resource] 23, no. 1 (2023): 1–9. 37580670 10.1186/s12876-023-02918-w PMC10426080

[248] L. Chi , L. Huan , C. Zhang , H. Wang , and J. Lu , “Adenosine receptor A2b confers ovarian cancer survival and PARP inhibitor resistance Through IL‐6‐STAT3 signalling,” Journal of Cellular and Molecular Medicine 27, no. 15 (2023): 2150–2164. 37278400 10.1111/jcmm.17802 PMC10399543

[249] M. Santoni , F. Miccini , A. Cimadamore , et al., “An update on investigational therapies that target STAT3 for the treatment of cancer,” Expert Opinion on Investigational Drugs 30, no. 3 (2021): 245–251. 33599169 10.1080/13543784.2021.1891222

[250] I. Tošić and D. A. Frank , “STAT3 as a mediator of oncogenic cellular metabolism: Pathogenic and therapeutic implications,” Neoplasia 23, no. 12 (2021): 1167–1178. 34731785 10.1016/j.neo.2021.10.003 PMC8569436

[251] Z. Hamel , S. Sanchez , D. Standing , and S. Anant , “Role of STAT3 in pancreatic cancer,” Exploration of Targeted Anti‐tumor Therapy 5, no. 5 (2024): 20. 38464736 10.37349/etat.2024.00202 PMC10918236

[252] Z. X. Niu , Y. T. Wang , J. F. Sun , P. Nie , and P. Herdewijn , “Recent advance of clinically approved small‐molecule drugs for the treatment of myeloid leukemia,” European Journal of Medicinal Chemistry 261 (2023): 115827. 37757658 10.1016/j.ejmech.2023.115827

[253] J. Greene , A. Segaran , and S. Lord , “Targeting OXPHOS and the electron transport chain in cancer; Molecular and therapeutic implications,” Seminars in Cancer Biology 86 (2022): 851–859. 35122973 10.1016/j.semcancer.2022.02.002

[254] D. Liang , Q. Wang , W. Zhang , et al., “JAK/STAT in leukemia: A clinical update,” Molecular Cancer 23, no. 1 (2024): 1–12. 38273387 10.1186/s12943-023-01929-1 PMC10811937

[255] A. Virzì , A. A. R. Suarez , T. F. Baumert , and J. Lupberger , “Rewiring Host Signaling: Hepatitis C Virus in Liver Pathogenesis,” Cold Spring Harbor Perspectives in Medicine 10, no. 1 (2020): a037366. 31501266 10.1101/cshperspect.a037366 PMC6938657

[256] H. Li , S. Ouyang , Y. Zhang , et al., “Structural optimization of Imidazo[1, 2‐a]pyridine derivatives for the treatment of gastric cancer via STAT3 signaling pathway,” European Journal of Medicinal Chemistry 244 (2022): 114858. 36283181 10.1016/j.ejmech.2022.114858

[257] Y. Li and Y. Dong , “TTI‐101 targets STAT3/c‐Myc signaling pathway to suppress cervical cancer progression: An integrated experimental and computational analysis,” Cancer Cell International 24, no. 1 (2024): 1–14. 39135042 10.1186/s12935-024-03463-6 PMC11320917

[258] D. J. Feith , J. Ung , O. Elghawy , P. Yue , J. Turkson , and T. P. Loughran , “Strategies for Targeting the JAK—STAT Pathway in Lymphoid Malignancies,” Precis Cancer Ther 1 (2023): 381–401.

[259] S. Ramchandani , C. D. Mohan , J. R. Mistry , et al., “The multifaceted antineoplastic role of pyrimethamine Against human malignancies,” Iubmb Life 74, no. 3 (2022): 198–212. 34921584 10.1002/iub.2590

[260] S. Molenda , A. Sikorska , A. Florczak , and P. Lorenc , “Dams‐Kozlowska H. Oligonucleotide‐Based Therapeutics for STAT3 Targeting in Cancer—Drug Carriers Matter,” Cancers 15, no. 23 (2023): 5647. 38067351 10.3390/cancers15235647 PMC10705165

[261] S. Song , H. Tang , T. Ran , et al., “Application of deep generative model for design of Pyrrolo[2,3‐d] pyrimidine derivatives as new selective TANK binding kinase 1 (TBK1) inhibitors,” European Journal of Medicinal Chemistry 247 (2023): 115034. 36603506 10.1016/j.ejmech.2022.115034

[262] J. Liu ;, F.; Wang , F. Luo , J. Liu , F. Wang , and F. Luo , “The Role of JAK/STAT Pathway in Fibrotic Diseases: Molecular and Cellular Mechanisms,” Biomol 13, no. 1 (2023): 119. 10.3390/biom13010119 PMC9855819 36671504

[263] Z. E. Walton , M. J. Frigault , and M. V. Maus , “Current and emerging pharmacotherapies for cytokine release syndrome, neurotoxicity, and hemophagocytic lymphohistiocytosis‐Like syndrome due to CAR T cell therapy,” Expert Opinion on Pharmacotherapy 25, no. 3 (2024): 263–279. 38588525 10.1080/14656566.2024.2340738

[264] C. Keenan , S. Albeituni , K. E. Nichols , and M. Hines , “JAK Inhibitors in Cytokine Storm Syndromes,” Advances in Experimental Medicine and Biology 1448 (2024): 583–600. 39117841 10.1007/978-3-031-59815-9_39

[265] R. Roskoski , “Janus kinase (JAK) inhibitors in the treatment of neoplastic and inflammatory disorders,” Pharmacological Research 183 (2022): 106362. 35878738 10.1016/j.phrs.2022.106362

[266] H. Zhang , F. He , G. Gao , et al., “Approved Small‐Molecule ATP‐Competitive Kinases Drugs Containing Indole/Azaindole/Oxindole Scaffolds: R&D and Binding Patterns Profiling,” Mol 28, no. 3 (2023): 943. 10.3390/molecules28030943 PMC9920796 36770611

[267] P. Shen , Y. Wang , X. Jia , et al., “Dual‐target Janus kinase (JAK) inhibitors: Comprehensive review on the JAK‐based strategies for treating solid or hematological malignancies and immune‐related diseases,” European Journal of Medicinal Chemistry 239 (2022): 114551. 35749986 10.1016/j.ejmech.2022.114551

[268] X. H. Wei and Y. Y. Liu , “Potential applications of JAK inhibitors, clinically approved drugs Against autoimmune diseases, in cancer therapy,” Frontiers in Pharmacology 14 (2023): 1326281. 38235120 10.3389/fphar.2023.1326281 PMC10792058

[269] K. Gerlach , K. Lechner , V. Popp , et al., “The JAK1/3 Inhibitor to Tofacitinib Suppresses T Cell Homing and Activation in Chronic Intestinal Inflammation,” J Crohn's Colitis 15, no. 2 (2021): 244–257. 10.1093/ecco-jcc/jjaa162 32808031

[270] M. Lensing and A. Jabbari , “An overview of JAK/STAT pathways and JAK inhibition in alopecia areata,” Frontiers in Immunology 13 (2022): 955035. 36110853 10.3389/fimmu.2022.955035 PMC9470217

[271] P. Beinhoff , L. Sabharwal , V. Udhane , et al., “Second‐generation jak2 inhibitors for advanced prostate cancer: Are we ready for clinical development?,” Cancers (Basel) 13, no. 20 (2021): 5204. 34680353 10.3390/cancers13205204 PMC8533841

[272] Y. Zhao , X. Zhang , X. Ding , et al., “Efficacy and safety of FLT3 inhibitors in monotherapy of hematological and solid malignancies: A systemic analysis of clinical trials,” Frontiers in Pharmacology 15 (2024): 1294668. 38828446 10.3389/fphar.2024.1294668 PMC11140126

[273] A. B. Griso , L. Acero‐Riaguas , and B. Castelo , “Cebrián‐Carretero JL, Sastre‐Perona A. Mechanisms of Cisplatin Resistance in HPV Negative Head and Neck Squamous Cell Carcinomas,” Cells 11, no. 3 (2022): 561. 35159370 10.3390/cells11030561 PMC8834318

[274] K. Okuyama , T. Naruse , and S. Yanamoto , “Tumor microenvironmental modification by the current target therapy for head and neck squamous cell carcinoma,” Journal of Experimental & Clinical Cancer Research 42, no. 1 (2023): 1–14. 37143088 10.1186/s13046-023-02691-4 PMC10161653

[275] S. K. Padda , K. L. Reckamp , M. Koczywas , et al., “A phase 1b study of erlotinib and momelotinib for the treatment of EGFR‐mutated, tyrosine kinase inhibitor‐naive metastatic non‐small cell lung cancer,” Cancer Chemotheraphy and Pharmacology 89, no. 1 (2022): 105–115. 10.1007/s00280-021-04369-0 PMC8739290 34773474

[276] N. Mahadik , D. Bhattacharya , A. Padmanabhan , K. Sakhare , K. P. Narayan , and R. Banerjee , “Targeting steroid hormone receptors for anti‐cancer therapy—A review on small molecules and nanotherapeutic approaches,” Wiley Interdiscip Rev Nanomedicine Nanobiotechnology 14, no. 2 (2022): e1755. 34541822 10.1002/wnan.1755

[277] V. АZolottsev , АS. Latysheva , V. S. Pokrovsky , I. I. Khan , and A. Y. Misharin , “Promising applications of steroid сonjugates for cancer research and treatment,” European Journal of Medicinal Chemistry 210 (2021): 113089. 10.1016/j.ejmech.2020.113089 33321260

[278] Y. Yue , Y. Lou , X. Liu , and X. Peng , “Vasculogenic mimicry in head and neck tumors: A narrative review,” Translational Cancer Research 10, no. 6 (2021): 3044. 35116612 10.21037/tcr-21-34 PMC8798303

[279] Y. W. Guo , L. Zhu , Y. T. Duan , et al., “Ruxolitinib induces apoptosis and pyroptosis of anaplastic thyroid cancer via the transcriptional inhibition of DRP1‐mediated mitochondrial fission,” Cell Death & Disease 15, no. 2 (2024): 1–18. 38336839 10.1038/s41419-024-06511-1 PMC10858168

[280] K. H. T. Dao , J. Gotlib , M. M. N. Deininger , et al., “Efficacy of Ruxolitinib in Patients With Chronic Neutrophilic Leukemia and Atypical Chronic Myeloid Leukemia,” Journal of Clinical Oncology 38, no. 10 (2020): 1006. 31880950 10.1200/JCO.19.00895 PMC7106977

[281] J. Mascarenhas , “Pacritinib for the treatment of patients With myelofibrosis and thrombocytopenia,” Expert Review of Hematology 15, no. 8 (2022): 671–684. 35983661 10.1080/17474086.2022.2112565

[282] Y. Yang , Y. Mou , L. X. Wan , et al., “Rethinking therapeutic strategies of dual‐target drugs: An update on pharmacological small‐molecule compounds in cancer,” Medicinal Research Reviews 44, no. 6 (2024): 2600–2623. 38769656 10.1002/med.22057

[283] P. Wu , J. Zhou , Y. Wu , and L. Zhao , “The emerging role of Interleukin 37 in bone homeostasis and inflammatory bone diseases,” International Immunopharmacology 98 (2021): 107803. 34091255 10.1016/j.intimp.2021.107803

[284] J. E. Lopes , J. L. Fisher , H. L. Flick , et al., “ALKS 4230: A novel engineered IL‐2 fusion protein With an improved cellular selectivity profile for cancer immunotherapy,” Journal for ImmunoTherapy of Cancer 8, no. 1 (2020): e000673. 32317293 10.1136/jitc-2020-000673 PMC7204809

[285] M. L. T. Nguyen , K. C. Bui , T. Scholta , et al., “Targeting interleukin 6 signaling by monoclonal antibody siltuximab on cholangiocarcinoma,” Journal of Gastroenterology and Hepatology 36, no. 5 (2021): 1334–1345. 33091158 10.1111/jgh.15307

[286] L. Kruk , M. Mamtimin , A. Braun , et al., “Inflammatory Networks in Renal Cell Carcinoma,” Cancers 15, no. 8 (2023): 2212. 37190141 10.3390/cancers15082212 PMC10136567

[287] F. van Rhee , A. Rosenthal , K. Kanhai , et al., “Siltuximab is associated With improved progression‐free survival in idiopathic multicentric Castleman disease,” Blood Advances 6, no. 16 (2022): 4773–4781. 35793409 10.1182/bloodadvances.2022007112 PMC9631655

[288] R. Tamura , K. Yoshihara , and T. Enomoto , “Therapeutic Strategies Focused on Cancer‐Associated Hypercoagulation for Ovarian Clear Cell Carcinoma,” Cancers 14, no. 9 (2022): 2125. 35565252 10.3390/cancers14092125 PMC9099459

[289] H. M. Lee , H. J. Lee , and J. E. Chang , “Inflammatory Cytokine: An Attractive Target for Cancer Treatment,” Biomed 10, no. 9 (2022): 2116. 10.3390/biomedicines10092116 PMC9495935 36140220

[290] S. Parakh , M. Ernst , and A. R. Poh , “Multicellular Effects of STAT3 in Non‐small Cell Lung Cancer: Mechanistic Insights and Therapeutic Opportunities,” Cancers 13, no. 24 (2021): 6228. 34944848 10.3390/cancers13246228 PMC8699548

[291] C. Ebersbach , A. M. K. Beier , C. Thomas , and H. H. H. Erb , “Impact of STAT Proteins in Tumor Progress and Therapy Resistance in Advanced and Metastasized Prostate Cancer,” Cancers 13, no. 19 (2021): 4854. 34638338 10.3390/cancers13194854 PMC8508518

[292] Y. Wang and Y. Zhang , “Prognostic role of interleukin‐6 in renal cell carcinoma: A meta‐analysis,” Clinical & Translational Oncology 22, no. 6 (2020): 835–843. 31410730 10.1007/s12094-019-02192-x

[293] J. Kaur , P. Singh , T. Enzler , and V. Sahai , “Emerging antibody therapies for pancreatic adenocarcinoma: A review of recent phase 2 trials,” Expert Opinion on Emerging Drugs 26, no. 2 (2021): 103–129. 33734833 10.1080/14728214.2021.1905795

[294] M. Narazaki and T. Kishimoto , “Current status and prospects of IL‐6–targeting therapy,” Expert Review of Clinical Pharmacology 15, no. 5 (2022): 575–592. 35791866 10.1080/17512433.2022.2097905

[295] A. B. Avci , E. Feist , and G. R. Burmester , “Targeting IL‐6 or IL‐6 Receptor in Rheumatoid Arthritis: What Have We Learned?,” Biodrugs 38, no. 1 (2024): 61. 37989892 10.1007/s40259-023-00634-1 PMC10789669

[296] K. Panuciak , M. Margas , K. Makowska , and M. Lejman , “Insights Into Modern Therapeutic Approaches in Pediatric Acute Leukemias,” Cells 11, no. 1 (2022): 139. 35011701 10.3390/cells11010139 PMC8749975

[297] I. M. Chen , M. Donia , C. A. Chamberlain , et al., “Phase 2 study of ipilimumab, nivolumab, and tocilizumab combined With stereotactic body radiotherapy in patients With refractory pancreatic cancer (TRIPLE‐R),” European Journal of Cancer 180 (2023): 125–133. 36592507 10.1016/j.ejca.2022.11.035

[298] M. Li , S. Li , R. Zhao , et al., “CD318 is a target of chimeric antigen receptor T cells for the treatment of colorectal cancer,” Clinical and Experimental Medicine 23, no. 6 (2023): 2409–2419. 36495368 10.1007/s10238-022-00967-1

[299] M. Choi , J. Shin , C. E. Lee , et al., “Immunogenic cell death in cancer immunotherapy,” BMB Reports 56, no. 5 (2023): 275–286. 37081756 10.5483/BMBRep.2023-0024 PMC10230015

[300] C. Liu , J. Zhou , S. Zhang , et al., “Mesenchymal stem cells‐derived IL‐6 promotes invasion and metastasis of oral squamous cell carcinoma via JAK‐STAT3 signalling,” Oral Diseases 30, no. 4 (2024): 2097–2109. 37249062 10.1111/odi.14617

[301] M. Markouli , F. Ullah , N. Omar , et al., “Recent Advances in Adult Post‐Transplant Lymphoproliferative Disorder,” Cancers 14, no. 23 (2022): 5949. 36497432 10.3390/cancers14235949 PMC9740763

[302] P. Ercilla‐Rodríguez , M. Sánchez‐Díez , N. Alegría‐Aravena , et al., “CAR‐T lymphocyte‐based cell therapies; mechanistic substantiation, applications and biosafety enhancement With suicide genes: New opportunities to melt side effects,” Frontiers in Immunology 15 (2024): 1333150. 39091493 10.3389/fimmu.2024.1333150 PMC11291200

[303] A. Das , K. J. Lavanya , Nandini , K. Kaur , and V. Jaitak , “Effectiveness of Selective Estrogen Receptor Modulators in Breast Cancer Therapy: An Update,” Current Medicinal Chemistry 30, no. 29 (2022): 3287–3314. 10.2174/0929867329666221006110528 36201273

[304] E. Zafar , M. F. Maqbool , A. Iqbal , et al., “A comprehensive review on anticancer mechanism of bazedoxifene,” Biotechnology and Applied Biochemistry 69, no. 2 (2022): 767–782. 33759222 10.1002/bab.2150

[305] C. Shi , T. Bopp , H. W. Lo , K. Tkaczuk , and J. Lin , “Bazedoxifene as a Potential Cancer Therapeutic Agent Targeting IL‐6/GP130 Signaling,” Current Oncology 31, no. 10 (2024): 5737–5751. 39451730 10.3390/curroncol31100426 PMC11505662

[306] Q. Huang , Y. Zhong , H. Dong , et al., “Revisiting signal transducer and activator of transcription 3 (STAT3) as an anticancer target and its inhibitor discovery: Where are we and where should we go?,” European Journal of Medicinal Chemistry 187 (2020): 111922. 31810784 10.1016/j.ejmech.2019.111922

[307] K. Taniguchi , M. Tsugane , and A. Asai , “A Brief Update on STAT3 Signaling: Current Challenges and Future Directions in Cancer Treatment,” Journal of Cell Communication and Signaling 2, no. 3 (2021): 181–194.

[308] K. N. Wang , K. Zhou , N. N. Zhong , et al., “Enhancing cancer therapy: The role of drug delivery systems in STAT3 inhibitor efficacy and safety,” Life Sciences 346 (2024): 122635. 38615745 10.1016/j.lfs.2024.122635

[309] R. Mikyskova , O. Sapega , M. Psotka , et al., “STAT3 inhibitor Stattic and its analogues inhibit STAT3 phosphorylation and modulate cytokine secretion in senescent tumour cells,” Molecular Medicine Reports 27, no. 4 (2023). 10.3892/mmr.2023.12968 PMC10018236 36825563

[310] G. D. Qian , J. Xu , X. X. Shen , et al., “BP‐1‐102 and silencing of Fascin‐1 by RNA interference inhibits the proliferation of mouse pituitary adenoma AtT20 cells via the signal transducer and activator of transcription 3/fascin‐1 pathway,” International Journal of Neuroscience 131, no. 8 (2021): 810–827. 32326790 10.1080/00207454.2020.1758088

[311] W. Liu , Z. Chu , C. Yang , et al., “Discovery of potent STAT3 inhibitors using structure‐based virtual screening, molecular dynamic simulation, and biological evaluation,” Frontiers in Oncology 13 (2023): 1287797. 38023173 10.3389/fonc.2023.1287797 PMC10652556

[312] X. Tan , X. Ma , Y. Dai , et al., “A large‐scale transcriptional analysis reveals herb‐derived ginsenoside F2 suppressing hepatocellular carcinoma via inhibiting STAT3,” Phytomedicine 120 (2023): 155031. 37666060 10.1016/j.phymed.2023.155031

[313] A. Ahamed , M. Hasan , A. Samanta , et al., “Prospective pharmacological potential of cryptotanshinone in cancer therapy,” Pharmacological Research ‐ Modern Chinese Medicine 9 (2023): 100308.

[314] K. Zhao , Q. Zhao , X. Dai , et al., “Alantolactone enhances the sensitivity of melanoma to MAPK pathway inhibitors by targeting inhibition of STAT3 activation and Down‐regulating stem cell markers,” Cancer Cell International 24, no. 1 (2024): 1–14. 38822350 10.1186/s12935-024-03371-9 PMC11143683

[315] L. Long , X. Fei , L. Chen , L. Yao , and X. Lei , “Potential therapeutic targets of the JAK2/STAT3 signaling pathway in triple‐negative breast cancer,” Frontiers in Oncology 14 (2024): 1381251. 38699644 10.3389/fonc.2024.1381251 PMC11063389

[316] S. Verdura , E. Cuyàs , V. Ruiz‐Torres , et al., “Lung cancer management With silibinin: A historical and translational perspective,” Pharmaceuticals 14, no. 6 (2021): 559. 34208282 10.3390/ph14060559 PMC8230811

[317] V. Damerell , M. S. Pepper , and S. Prince , “Molecular mechanisms underpinning sarcomas and implications for current and future therapy,” Signal Transduction and Targeted Therapy 6, no. 1 (2021): 1–19. 34188019 10.1038/s41392-021-00647-8 PMC8241855

[318] P. S. Thilakasiri , R. S. Dmello , T. L. Nero , M. W. Parker , M. Ernst , and A. L. Chand , “Repurposing of drugs as STAT3 inhibitors for cancer therapy,” Seminars in Cancer Biology 68 (2021): 31–46. 31711994 10.1016/j.semcancer.2019.09.022

[319] Y. Chen , N. Zhai , Y. Zhu , et al., “Azetidine ring, salicylic acid, and salicylic acid bioisosteres as determinants of the binding characteristics of novel potent compounds to Stat3,” Bioorganic & Medicinal Chemistry Letters 97 (2024): 129565. 38008341 10.1016/j.bmcl.2023.129565

[320] A. Caruso , A. Barbarossa , A. Carocci , G. Salzano , M. S. Sinicropi , and C. Saturnino , “Carbazole Derivatives as STAT Inhibitors: An Overview,” Applied Science Letters 11, no. 13 (2021): 6192.

[321] N. Deravi and N. Rezaei . Signal Transducer and Activator of Transcription as a Potential Therapeutic Target in Breast Cancer, published online 2023: 1–26, 10.1007/16833_2022_107.

[322] F. Shao , X. Pang , and G. H. Baeg , “Targeting the JAK/STAT Signaling Pathway for Breast Cancer,” Current Medicinal Chemistry 28, no. 25 (2020): 5137–5151. 10.2174/0929867328666201207202012 33290193

[323] P. Yue , Y. Zhu , C. Brotherton‐Pleiss , et al., “Novel potent azetidine‐based compounds irreversibly inhibit Stat3 activation and induce antitumor response Against human breast tumor growth in vivo,” Cancer Letters 534 (2022): 215613. 35276290 10.1016/j.canlet.2022.215613 PMC9867837

[324] M. A. Thalappil , Targeting STAT3 signaling with essential oils: a potential strategy for adjuvant cancer therapy, published online 2023, accessed November 18, 2024. https://iris.univr.it/handle/11562/1099367.

[325] E. Khatoon , M. Hegde , A. Kumar , et al., “The multifaceted role of STAT3 pathway and its implication as a potential therapeutic target in oral cancer,” Archives of Pharmacal Research 45, no. 8 (2022): 507–534. 35987863 10.1007/s12272-022-01398-y

[326] E. L. Morgan and A. Macdonald , “Manipulation of JAK/STAT Signalling by High‐Risk HPVs: Potential Therapeutic Targets for HPV‐Associated Malignancies,” Viruses. 12, no. 9 (2020): 977. 32899142 10.3390/v12090977 PMC7552066

[327] J. Xu , J. Zhang , Q. F. Mao , J. Wu , and Y. Wang , “The Interaction Between Autophagy and JAK/STAT3 Signaling Pathway in Tumors,” Frontiers in Genetics 13 (2022). 10.3389/fgene.2022.880359 PMC9086235 35559037

[328] D. P. McLornan , J. E. Pope , J. Gotlib , and C. N. Harrison , “Current and future status of JAK inhibitors,” Lancet 398, no. 10302 (2021): 803–816. 34454676 10.1016/S0140-6736(21)00438-4

[329] B. Rah , R. A. Rather , G. R. Bhat , et al., “JAK/STAT Signaling: Molecular Targets, Therapeutic Opportunities, and Limitations of Targeted Inhibitions in Solid Malignancies,” Frontiers in Pharmacology 13 (2022): 821344. 35401182 10.3389/fphar.2022.821344 PMC8987160

[330] N. R. Jabir , C. K. Firoz , M. A. Kamal , et al., “Assessment of genetic diversity in IL‐6 and RANTES promoters and their level in Saudi coronary artery disease patients,” Journal of Clinical Laboratory Analysis 31, no. 5 (2017): e22092. 27862306 10.1002/jcla.22092 PMC6816867

[331] D. Aletaha , A. Kerschbaumer , K. Kastrati , et al., “Consensus statement on blocking interleukin‐6 receptor and interleukin‐6 in inflammatory conditions: An update,” Annals of the Rheumatic Diseases 82, no. 6 (2023): 773–787. 35953263 10.1136/ard-2022-222784

[332] P. L. Yang , L. X. Liu , E. M. Li , and L. Y. Xu , “STAT3, the Challenge for Chemotherapeutic and Radiotherapeutic Efficacy,” Cancers 12, no. 9 (2020): 2459. 32872659 10.3390/cancers12092459 PMC7564975

[333] Y. Jiang , L. Liu , Y. Geng , et al., “Feasibility of the inhibitor development for cancer: A systematic approach for drug design,” PLoS ONE 19, no. 8 (2024): e0306632. 39173044 10.1371/journal.pone.0306632 PMC11341021

[334] X. Yang , L. Xu , L. Yang , and S. Xu , “Research progress of STAT3‐based dual inhibitors for cancer therapy,” Bioorganic & Medicinal Chemistry 91 (2023): 117382. 37369169 10.1016/j.bmc.2023.117382

[335] T. W. Kim , Y. Kim , H. Keum , W. Jung , M. Kang , and S. Jon , “Combination of a STAT3 inhibitor With anti‐PD‐1 immunotherapy is an effective treatment regimen for a vemurafenib‐resistant melanoma,” Molecular Therapy Oncolytics 26 (2022): 1–14. 35784401 10.1016/j.omto.2022.06.001 PMC9218293

[336] Y. Zhu , M. Chen , D. Xu , et al., “The combination of PD‐1 blockade With interferon‐α has a synergistic effect on hepatocellular carcinoma,” Cellular & Molecular Immunology 19, no. 6 (2022): 726–737. 35459855 10.1038/s41423-022-00848-3 PMC9151669

[337] M. Markouli , D. Strepkos , and C. Piperi , “RETRACTED: Impact of Histone Modifications and Their Therapeutic Targeting in Hematological Malignancies,” International Journal of Molecular Sciences 23, no. 21 (2022): 13657. 36362442 10.3390/ijms232113657 PMC9654260

[338] X. Chen , L. Pan , J. Wei , et al., “LLL12B, a small molecule STAT3 inhibitor, induces growth arrest, apoptosis, and enhances cisplatin‐mediated cytotoxicity in medulloblastoma cells,” Science Reports 11, no. 1 (2021): 1–14. 10.1038/s41598-021-85888-x PMC7985203 33753770

[339] S. H. Lee , C. X. Ng , S. R. Wong , and P. P. Chong , “MiRNAs Overexpression and Their Role in Breast Cancer: Implications for Cancer Therapeutics,” Current Drug Targets 24, no. 6 (2023): 484–508. 36999414 10.2174/1389450124666230329123409

[340] A. J. Oweida , L. Darragh , A. Phan , et al., “STAT3 Modulation of Regulatory T Cells in Response to Radiation Therapy in Head and Neck Cancer,” Journal of the National Cancer Institute 111, no. 12 (2019): 1339–1349. 30863843 10.1093/jnci/djz036 PMC6910208

[341] Y. Ni , J. T. Low , J. Silke , and L. A. O'Reilly , “Digesting the Role of JAK‐STAT and Cytokine Signaling in Oral and Gastric Cancers,” Frontiers in Immunology 13 (2022): 835997. 35844493 10.3389/fimmu.2022.835997 PMC9277720

[342] W. Wang , M. C. Lopez McDonald , R. Hariprasad , T. Hamilton , and D. A. Frank , “Oncogenic STAT Transcription Factors as Targets for Cancer Therapy: Innovative Strategies and Clinical Translation,” Cancers 16, no. 7 (2024): 1387. 38611065 10.3390/cancers16071387 PMC11011165

[343] T. Hu , R. Shi , Y. Gu , et al., “Cancer‐derived non‐coding RNAs endow tumor microenvironment With immunosuppressive properties,” Wiley Interdisciplinary Reviews RNA 15, no. 1 (2024): e1822. 10.1002/wrna.1822 37817381

[344] C. J. Chen , H. C. Wang , Y. Y. Wu , C. C. Shieh , and Y. S. Shan , A New Gene Therapy for Pancreatic Cancer: STAT6‐CYBB Decoy Oligodeoxynucleotides Focusing on Inhibiting M2 Macrophages, published online May 31, 2022, 10.21203/RS.3.RS-1690410/V1.

[345] M. Jiang and B. Li , “STAT3 and Its Targeting Inhibitors in Oral Squamous Cell Carcinoma,” Cells 11, no. 19 (2022): 3131. 36231093 10.3390/cells11193131 PMC9563058

[346] Y. Pan , J. Cheng , Y. Zhu , J. Zhang , W. Fan , and X. Chen , “Immunological nanomaterials to combat cancer metastasis,” Chemical Society Reviews 53, no. 12 (2024): 6399–6444. 38745455 10.1039/d2cs00968d

[347] M. Corte‐Real , F. Veiga , A. C. Paiva‐Santos , and P. C. Pires , “Improving Skin Cancer Treatment by Dual Drug Co‐Encapsulation Into Liposomal Systems—An Integrated Approach towards Anticancer Synergism and Targeted Delivery,” Pharmaceutics 16, no. 9 (2024): 1200. 39339235 10.3390/pharmaceutics16091200 PMC11434718

[348] A. S. Widjaya , Y. Liu , Y. Yang , W. Yin , J. Liang , and Y. Jiang , “Tumor‐permeable smart liposomes by modulating the tumor microenvironment to improve the chemotherapy,” Journal of Controlled Release 344 (2022): 62–79. 35182612 10.1016/j.jconrel.2022.02.020

[349] G. Basirinia , M. Ali , A. Comelli , et al., “Theranostic Approaches for Gastric Cancer: An Overview of In Vitro and In Vivo Investigations,” Cancers 16, no. 19 (2024): 3323. 39409942 10.3390/cancers16193323 PMC11476023

[350] S. Kumari , P. K. Choudhary , R. Shukla , A. Sahebkar , and P. Kesharwani , “Recent advances in nanotechnology based combination drug therapy for skin cancer,” Journal of Biomaterials Science, Polymer Edition 33, no. 11 (2022): 1435–1468. 35294334 10.1080/09205063.2022.2054399

[351] Y. Jiang , C. Yan , M. Li , et al., “Delivery of natural products via polysaccharide‐based nanocarriers for cancer therapy: A review on recent advances and future challenges,” International Journal of Biological Macromolecules 278 (2024): 135072. 39191341 10.1016/j.ijbiomac.2024.135072

[352] S. Abdullah , F. Goher , and A. N. Awan , Nanoparticles: A Treatment Modality for Lung Cancer, published online 2024: 139–159, 10.1007/16833_2024_306.

[353] Y. Dong , J. Chen , Y. Chen , and S. Liu , “Targeting the STAT3 oncogenic pathway: Cancer immunotherapy and drug repurposing,” Biomedicine & Pharmacotherapy 167 (2023): 115513. 37741251 10.1016/j.biopha.2023.115513

[354] R. Bellavita , S. Braccia , A. Falanga , and S. Galdiero , “An Overview of Supramolecular Platforms Boosting Drug Delivery,” Bioinorganic Chemistry and Applications 2023, no. 1 (2023): 8608428. 38028018 10.1155/2023/8608428 PMC10661875

[355] M. R. Tavares , K. Hrabánková , R. Konefał , et al., “HPMA‐Based Copolymers Carrying STAT3 Inhibitor Cucurbitacin‐D as Stimulus‐Sensitive Nanomedicines for Oncotherapy,” Pharmaceutics 13, no. 2 (2021): 179. 33525658 10.3390/pharmaceutics13020179 PMC7911143

[356] T. Hu , H. Gong , J. Xu , Y. Huang , F. Wu , and Z. He , “Nanomedicines for Overcoming Cancer Drug Resistance,” Pharmaceutics 14, no. 8 (2022): 1606. 36015232 10.3390/pharmaceutics14081606 PMC9412887

[357] Y. Sun , M. Li , M. Zheng , Y. Zou , and B. Shi , “Blood‐brain barrier penetrating nanosystems enable synergistic therapy of glioblastoma,” Nano Today 56 (2024): 102310.

[358] N. Bie , T. Yong , Z. Wei , L. Gan , and X. Yang , “Extracellular vesicles for improved tumor accumulation and penetration,” Advanced Drug Delivery Reviews 188 (2022): 114450. 35841955 10.1016/j.addr.2022.114450

[359] W. Ngamcherdtrakul , M. Reda , M. A. Nelson , et al., “In Situ Tumor Vaccination With Nanoparticle Co‐Delivering CpG and STAT3 siRNA to Effectively Induce Whole‐Body Antitumor Immune Response,” Advanced Materials 33, no. 31 (2021): 2100628. 10.1002/adma.202100628 PMC8424660 34118167

[360] L. Yang , Q. Hu , and T. Huang , “Breast Cancer Treatment Strategies Targeting the Tumor Microenvironment: How to Convert “Cold” Tumors to “Hot” Tumors,” International Journal of Molecular Sciences 25, no. 13 (2024): 7208. 39000314 10.3390/ijms25137208 PMC11241188

[361] F. Della Sala , A. Fabozzi , M. di Gennaro , et al., “Advances in Hyaluronic‐Acid‐Based (Nano)Devices for Cancer Therapy,” Macromolecular Bioscience 22, no. 1 (2022): 2100304. 10.1002/mabi.202100304 34657388

[362] S. Karthik , S. Mohan , I. Magesh , et al., “Chitosan nanocarriers for non‐coding RNA therapeutics: A review,” International Journal of Biological Macromolecules 263 (2024): 130361. 38395284 10.1016/j.ijbiomac.2024.130361

[363] A. Ahmad , S. Rashid , A. A. Chaudhary , et al., “Nanomedicine as potential cancer therapy via targeting dysregulated transcription factors,” Seminars in Cancer Biology 89 (2023): 38–60. 36669712 10.1016/j.semcancer.2023.01.002

[364] K. Thapa Magar , G. F. Boafo , X. Li , Z. Chen , and W. He , “Liposome‐based delivery of biological drugs,” Chinese Chemical Letters 33, no. 2 (2022): 587–596.

[365] J. Zhao , J. Yang , J. Jiao , X. Wang , Y. Zhao , and L. Zhang , “Biomedical applications of artificial exosomes for intranasal drug delivery,” Frontiers in Bioengineering and Biotechnology 11 (2023): 1271489. 37744256 10.3389/fbioe.2023.1271489 PMC10513441

[366] B. You , C. Jin , J. Zhang , et al., “MSC‐Derived Extracellular Vesicle‐Delivered L‐PGDS Inhibit Gastric Cancer Progression by Suppressing Cancer Cell Stemness and STAT3 Phosphorylation,” Stem Cells International 2022 (2022). 10.1155/2022/9668239 PMC8789473 35087591

[367] S. F. Liang , F. F. Zuo , B. C. Yin , and B. C. Ye , “Delivery of siRNA based on engineered exosomes for glioblastoma therapy by targeting STAT3,” Biomaterials Science 10, no. 6 (2022): 1582–1590. 35179533 10.1039/d1bm01723c

[368] T. Chen , B. Ma , S. Lu , et al., “Cucumber‐Derived Nanovesicles Containing Cucurbitacin B for Non‐Small Cell Lung Cancer Therapy,” International Journal of Nanomedicine 17 (2022): 3583–3599. 35974872 10.2147/IJN.S362244 PMC9376005

[369] I. Ahmad , S. Ahmad , A. Ahmad , T. A. Zughaibi , M. Alhosin , and S. Tabrez , “Curcumin, its derivatives, and their nanoformulations: Revolutionizing cancer treatment,” Cell Biochemistry and Function 42, no. 1 (2024). 10.1002/cbf.3911 38269517

[370] D. Guimarães , A. Cavaco‐Paulo , and E. Nogueira , “Design of liposomes as drug delivery system for therapeutic applications,” International Journal of Pharmaceutics 601 (2021): 120571. 33812967 10.1016/j.ijpharm.2021.120571

[371] P. Liu , G. Chen , and J. Zhang , “A Review of Liposomes as a Drug Delivery System: Current Status of Approved Products, Regulatory Environments, and Future Perspectives,” Mol 27, no. 4 (2022): 1372. 10.3390/molecules27041372 PMC8879473 35209162

[372] K. Shi , J. Xue , Y. Fang , et al., “Inorganic Kernel‐Reconstituted Lipoprotein Biomimetic Nanovehicles Enable Efficient Targeting “Trojan Horse” Delivery of STAT3‐Decoy Oligonucleotide for Overcoming TRAIL Resistance,” Theranostics 7, no. 18 (2017): 4480–4497. 29158840 10.7150/thno.21707 PMC5695144

[373] K. Veselá , Z. Kejík , M. Masařík , et al., “Curcumin: A Potential Weapon in the Prevention and Treatment of Head and Neck Cancer,” ACS Pharmacology & Translational Science 7, no. 11 (2024): 3394–3418. 39539276 10.1021/acsptsci.4c00518 PMC11555516

[374] F. Zahedipour , M. Bolourinezhad , Y. Teng , and A. Sahebkar , “The Multifaceted Therapeutic Mechanisms of Curcumin in Osteosarcoma: State‐of‐the‐Art,” Journal of Oncology 2021, no. 1 (2021): 3006853. 34671398 10.1155/2021/3006853 PMC8523229

[375] T. Ikeda , M. Kawabori , Y. Zheng , et al., “Intranasal Administration of Mesenchymal Stem Cell‐Derived Exosome Alleviates Hypoxic‐Ischemic Brain Injury,” Pharmaceutics 16, no. 4 (2024): 446. 38675108 10.3390/pharmaceutics16040446 PMC11053690

[376] H. Zheng , Z. Chen , A. Cai , et al., “Nanoparticle mediated codelivery of nifuratel and doxorubicin for synergistic anticancer therapy Through STAT3 inhibition,” Colloids Surfaces B Biointerfaces 193 (2020): 111109. 32416521 10.1016/j.colsurfb.2020.111109

[377] Z. Li , G. Chen , L. Ding , et al., “Increased Survival by Pulmonary Treatment of Established Lung Metastases With Dual STAT3/CXCR4 Inhibition by siRNA Nanoemulsions,” Molecular Therapy 27, no. 12 (2019): 2100–2110. 31481310 10.1016/j.ymthe.2019.08.008 PMC6904825

[378] H. Tabasi , S. Mollazadeh , E. Fazeli , et al., “Transitional Insight Into the RNA‐Based Oligonucleotides in Cancer Treatment,” Applied Biochemistry and Biotechnology 196, no. 3 (2023): 1685–1711. 37402038 10.1007/s12010-023-04597-5

[379] N. O. Alafaleq , T. A. Zughaibi , N. R. Jabir , A. U. Khan , M. S. Khan , and S. Tabrez , “Biogenic Synthesis of Cu‐Mn Bimetallic Nanoparticles Using Pumpkin Seeds Extract and Their Characterization and Anticancer Efficacy,” Nanomater (Basel, Switzerland) 13, no. 7 (2023). 10.3390/nano13071201 PMC10096695 37049295

[380] T. A. Zughaibi , N. R. Jabir , A. U. Khan , M. S. Khan , and S. Tabrez , “Screening of Cu4O3 NPs efficacy and its anticancer potential Against cervical cancer,” Cell Biochemistry and Function 41, no. 8 (2023): 1174–1187. 37691077 10.1002/cbf.3850

[381] M. Rashid Khan , N. Omar Alafaleq , A. Kumar Ramu , et al., “Evaluation of biogenically synthesized MgO NPs anticancer activity Against breast cancer cells,” Saudi Journal of Biological Sciences 31, no. 1 (2024). 10.1016/j.sjbs.2023.103874 PMC10711182 38090134

[382] L. Sun , Z. Li , J. Lan , Y. Wu , T. Zhang , and Y. Ding , “Better together: Nanoscale co‐delivery systems of therapeutic agents for high‐performance cancer therapy,” Frontiers in Pharmacology 15 (2024): 1389922. 38831883 10.3389/fphar.2024.1389922 PMC11144913

[383] N. Pore , S. Wu , N. Standifer , et al., “Resistance to durvalumab and durvalumab plus tremelimumab is associated With functional STK11 mutations in patients With non–small cell lung cancer and is reversed by STAT3 knockdown,” Cancer Discovery 11, no. 11 (2021): 2828–2845. 34230008 10.1158/2159-8290.CD-20-1543

[384] D. Mukherjee and S. Raikwar , “Recent Update on Nanocarrier(s) as the Targeted Therapy for Breast Cancer,” Aaps Pharmscitech [Electronic Resource] 25, no. 6 (2024): 1–25. 10.1208/s12249-024-02867-x 38961013

[385] M. I. Khan , M. I. Hossain , M. K. Hossain , et al., “Recent Progress in Nanostructured Smart Drug Delivery Systems for Cancer Therapy: A Review,” ACS Applied Bio Materials 5, no. 3 (2022): 971–1012. 10.1021/acsabm.2c00002 35226465

[386] Y. Zhang , Y. Wu , H. Du , et al., “Nano‐Drug Delivery Systems in Oral Cancer Therapy: Recent Developments and Prospective,” Pharmaceutics 16, no. 1 (2023): 7. 38276483 10.3390/pharmaceutics16010007 PMC10820767

[387] K. K. Chenab , H. Malektaj , A. A. R. Nadinlooie , S. Mohammadi , and M. R. Zamani‐Meymian , “Intertumoral and intratumoral barriers as approaches for drug delivery and theranostics to solid tumors using stimuli‐responsive materials,” Microchimica Acta 191, no. 9 (2024): 1–51. 10.1007/s00604-024-06583-y 39150483

[388] B. Yang , F. Meng , J. Zhang , et al., “Engineered drug delivery nanosystems for tumor microenvironment normalization therapy,” Nano Today 49 (2023): 101766.

[389] C. Pacheco , A. Baião , T. Ding , W. Cui , and B. Sarmento , “Recent advances in long‐acting drug delivery systems for anticancer drug,” Advanced Drug Delivery Reviews 194 (2023): 114724. 36746307 10.1016/j.addr.2023.114724

[390] H. Xie , Z. Chen , N. Zhang , et al., “A review of recent advances in the stability, efficacy, and biosafety of black phosphorus‐based drug delivery,” Journal of Materials Science 59, no. 27 (2024): 12129–12153.

